# A New Family of Ternary Intermetallic Compounds with Dualistic Atomic Ordering – The ZIP Phases

**DOI:** 10.1002/adma.202308168

**Published:** 2025-09-10

**Authors:** Matheus A. Tunes, Sean M. Drewry, Franziska Schmidt, James A. Valdez, Matthew M. Schneider, Caitlin A. Kohnert, Tarik A. Saleh, Saryu Fensin, Stuart A. Maloy, Cláudio G. Schön, Sylvain Dubois, Omri Tabo, Anna Eyal, Amit Keren, Asaf Pesach, Ganesh K. Nayak, Stavros‐Richard G. Christopoulos, Marco Molinari, Marcus Hans, Nick Goossens, Shuigen Huang, Jochen M. Schneider, Per O. Å. Persson, Jozef Vleugels, Konstantina Lambrinou

**Affiliations:** ^1^ Department Metallurgy Chair of Nonferrous Metallurgy Montanuniversität Leoben Franz‐Josef‐Strasse 18 Leoben 8700 Austria; ^2^ Department of Engineering School of Computing and Engineering University of Huddersfield Queensgate Huddersfield HD1 3DH UK; ^3^ Department of Computer Science School of Computing and Engineering University of Huddersfield Queensgate Huddersfield HD1 3DH UK; ^4^ Department of Physical and Life Sciences School of Applied Sciences University of Huddersfield Queensgate Huddersfield HD1 3DH UK; ^5^ Materials Science and Technology Division Los Alamos National Laboratory (LANL) Bikini Atoll Rd Los Alamos NM 87544 USA; ^6^ Pacific Northwest National Laboratory (PNNL) 902 Battelle Blvd Richland WA 99352 USA; ^7^ Department of Metallurgical and Materials Engineering Universidade de São Paulo Escola Politécnica Av. Prof. Mello Moraes 2463 São Paulo CEP 05508‐030 Brazil; ^8^ Institut PPRIME Département de Physique et Mécanique des Matériaux CNRS Université de Poitiers ENSMA UPR 3346, SP2MI, Téléport 2, Boulevard Marie et Pierre Curie, Futuroscope, Chasseneuil Cedex, 30179 Poitiers Nouvelle‐Aquitaine 86962 France; ^9^ Faculty of Physics Technion – Israel Institute of Technology Haifa 32000 Israel; ^10^ Department of Physics Nuclear Research Centre – Negev P.O. Box 9001 Beer Sheva 84190 Israel; ^11^ Materials Chemistry RWTH Aachen University Kopernikusstr. 10 D‐52074 Aachen Germany; ^12^ Thin Film Physics Department of Physics Chemistry and Biology Linköping University Linköping SE‐581 83 Sweden; ^13^ Department of Materials Engineering KU Leuven Kasteelpark Arenberg 44 Leuven 3001 Belgium; ^14^ Italian Institute of Technology (IIT) Via Morego 30 Genova 16163 Italy; ^15^ Department of Materials Science and Engineering University of Tennessee 414 Ferris Hall, 1508 Middle Drive Knoxville TN 37996‐2100 USA; ^16^ Department of Nuclear Engineering University of California Berkeley 4153 Etcheverry Hall, 1730 Berkeley CA 94720‐1730 USA; ^17^ Laboratory of High‐Performance Ceramics Swiss Federal Laboratories for Materials Science Technology EMPA Überlandstrasse 129 Dübendorf CH‐8600 Switzerland

**Keywords:** Intermetallic compounds (IMCs), MAX phases, MXenes, nanolaminated hexagonal solids & 2D derivatives, nanostructured solids, ZIP phases

## Abstract

A new family of nanostructured ternary intermetallic compounds − named the ZIP phases − is introduced in this work. The ZIP phases exhibit dualistic atomic ordering, i.e., they form two structural variants: one with the *fcc* diamond cubic structure (space group *Fd* $\bar{3}$ *m*) and one with the hexagonal structure (space group *P6_3_/mmc*). They are also characterized by metallic behavior, ionic bonding, and atomic zigzagging. Powder metallurgical routes involving pressure‐assisted densification are adopted to demonstrate ZIP phase synthesis in the Nb‐Si‐Ni, Nb‐Si‐Co, Ta‐Si‐Ni, V‐Si‐Ni, and Nb‐Si‐Fe ternary systems. Crucially, reactive hot pressing is capable of producing high‐purity ZIP phase materials after the judicious, elemental system‐specific optimization of the processing route. Synthesis of phase‐pure materials – demonstrated in the Nb‐Si‐Ni ternary system by the synthesis of quasi phase‐pure Nb_3_SiNi_2_ and Ni_3_SiNb_2_ ZIP phase‐based materials – is a steppingstone to the prospective exploitation of the ZIP phases. Characterization of Nb_3_SiNi_2_ and Ni_3_SiNb_2_ involves crystal structure determination, spatially resolved chemical analysis, and determination of select thermal, electrical, magnetic, mechanical, and physical properties. Density functional theory is used to assess the stability of Nb_3_SiNi_2_ & Ni_3_SiNb_2_ and derivative binary compounds at different temperatures, also exploring the exfoliation of these two ZIP phases along specific surfaces to produce 2D derivatives.

## Graphical abstract

The ZIP phases are ternary intermetallic compounds with dualistic atomic ordering, i.e., they exhibit one face‐centered cubic (*fcc*; space group *Fd* $\bar{3}$ *m*) variant and one hexagonal (space group *P6_3_/mmc*) variant. The ZIP phases in the Nb‐Si‐Ni system are the Nb_3_SiNi_2_ (*fcc*) and Ni_3_SiNb_2_ (hexagonal) ternary IMCs, crystal structure schematics of which are pasted on the yin‐yang Chinese cosmological symbol of duality.

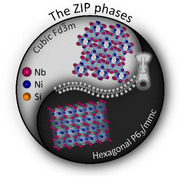

## 1 Introduction

A revolution in materials science was undoubtedly achieved through the microstructural manipulation of materials on the nanoscale.^[^ ^1^, ^2^ ^]^ When the dimensions of the basic building blocks of an engineering material are confined in the 1–100 nm range, the material can be defined as nanostructured.^[^ ^3^ ^]^ The nanoscale manipulation of matter via the (application‐driven) tailoring of structural “modules”, such as ordered atomic clusters, precipitates and grains, engineered grain boundaries, complex molecules, and atomically thin layers is an effective approach to achieve a set of exceptional properties in advanced materials, well beyond what is possible by conventional processing routes that aim at modifications on the microscale, mesoscale, and/or macroscale.^[^ ^1^, ^2^, ^3^, ^4^ ^]^ Transferring the superior properties of nanostructured materials to the macroscale relies on judicious multiscale materials engineering and remains one of the key challenges of contemporary materials science.^[^ ^4^ ^]^

Gleiter, the scientist who coined the term “nanostructured materials” in 1992, predicted that this novel research field would be greatly impacted by the emergence of two classes of nanostructured materials, i.e., ceramics and intermetallic phases.^[^ ^2^ ^]^ Gleiter's prediction came partly true in 1996, when Barsoum and his group made significant strides in the development and characterization of a new class of nanolaminated ceramics with the M_n+1_AX_n_ general stoichiometry, where M is an early transition metal, A is an element mainly from groups 13–15 in the periodic table, X is C or N, and n is an integer (commonly, 1, 2, or 3); these ceramics became known as the MAX phases.^[^ ^5^, ^6^ ^]^ The 1996 breakthrough by Barsoum and El‐Raghy^[^ ^5^ ^]^ consisted in the key synthesis of quasi phase‐pure Ti_3_SiC_2_ via reactive hot pressing; notably, the existence of the Ti_3_SiC_2_ compound, which is regarded as the H‐phase of the ternary Ti‐Si‐C system,^[^ ^7^ ^]^ had first been reported in 1967 by Jeitschko and Nowotny.^[^ ^8^ ^]^ Starting by the successful experimental synthesis and characterization of Ti_3_SiC_2_, i.e., the 312 MAX phase of the Ti‐Si‐C system,^[^ ^7^ ^]^ Barsoum realized in the early 2000s that Ti_3_SiC_2_ and other (similar in nature) nanolaminated ternary carbides/nitrides form an entire class of hexagonal solids (space group *P6_3_/mmc*) obeying the M_n+1_AX_n_ stoichiometric rule and in possession of exceptional physicochemical and mechanical properties.^[^ ^6^ ^]^ To date, about 155 ternary carbides and nitrides of early transition metals, as well as numerous higher order (chemically complex) solid solutions with the M_n+1_AX_n_ overarching stoichiometry and precisely tailored properties have been synthesized.^[^ ^9^, ^10^ ^]^ The exceptional properties of the MAX phases are attributed to their nanolaminated hexagonal crystal structure, where n M_6_X (ceramic‐like) octahedra are interleaved with a single A (metallic‐like) atomic layer, and the M‐atoms form a trigonal prism with the A‐element in its center.^[^ ^10^ ^]^ Therefore, the MAX phase structure can be perceived as a combination of two building blocks, i.e., M_6_X octahedra with the rock‐salt (NaCl‐type) structure and M_6_A‐trigonal prisms with the hexagonal closed packed (*hcp*) structure.^[^ ^10^ ^]^ Since their inception, the MAX phases^[^ ^11^, ^12^ ^]^ and their 2D derivatives known as MXenes^[^ ^13^, ^14^, ^15^, ^16^, ^17^, ^18^ ^]^ have been proposed for promising niche applications in diverse fields, including energy storage, fuel cells, biomedical applications, photonics/phononics,^[^ ^19^, ^20^, ^21^, ^22^, ^23^, ^24^, ^25^, ^26^, ^27^, ^28^, ^29^, ^30^, ^31^ ^]^ as well as advanced fusion and fission nuclear systems.^[^ ^32^, ^33^, ^34^, ^35^, ^36^, ^37^, ^38^, ^39^, ^40^, ^41^, ^42^, ^43^ ^]^

This work introduces a new family of nanostructured ternary intermetallic compounds (IMCs), herein aptly named “zigzag intermetallic compounds”, or simply the ZIP phases, due to their distinctive “zigzag” atomic arrangement. Each member of the ZIP phase family has two structural variants, i.e., one with the *fcc* diamond cubic structure (space group *Fd* $\bar{3}$ *m*) and one with the hexagonal structure (space group *P6_3_/mmc*). Transitioning between ZIP phase variants requires elemental redistribution in the “312” stoichiometry describing these materials. For example, the two ZIP phases in the Nb‐Si‐Ni system are the *fcc* Nb_3_SiNi_2_ IMC, also regarded as the H‐phase of the ternary Nb‐Si‐Ni system, and the hexagonal Ni_3_SiNb_2_ IMC.^[^ ^44^, ^45^, ^46^, ^47^ ^]^ The hexagonal variants of the ZIP phases exhibit striking similarities with the MAX phases, including crystal symmetry (i.e., they share space group *P6_3_/mmc*), crystal structure nanolamination, and the 312 MAX phase stoichiometry; however, the extent of this resemblance, especially in terms of potential applications, cannot be properly assessed until materials with high phase purity have been synthesized. This work reports, for the first time, the experimental synthesis of quasi phase‐pure Nb_3_SiNi_2_ and Ni_3_SiNb_2_ via reactive hot pressing, enabling the detailed characterization of ZIP phases in the Nb‐Si‐Ni ternary system. ZIP phases have also been synthesized in the Nb‐Si‐Co, Ta‐Si‐Ni, V‐Si‐Ni, and Nb‐Si‐Fe ternary systems by means of reactive hot pressing and spark plasma sintering. Moreover, basic aspects of the potential formation of 2D derivatives of the ZIP phases – similar to the 2D derivatives of the MAX phases broadly known as MXenes – have been explored using density functional theory in conjunction with the temperature‐dependent effective potential method. The introduction of the ZIP phase family of ternary IMCs in this work validates Gleiter's 1992 prediction regarding the emergence and diverse potential benefits of “nanostructured intermetallics”.^[^ ^1^, ^2^ ^]^

## 2 Results and Discussion

### 2.1 The Gateway to the Discovery of the ZIP Phases – The Nb‐Si‐Ni System

#### 2.1.1 Synthesis of Nb_3_SiNi_2_ by Arc Melting

**Figure 1:**
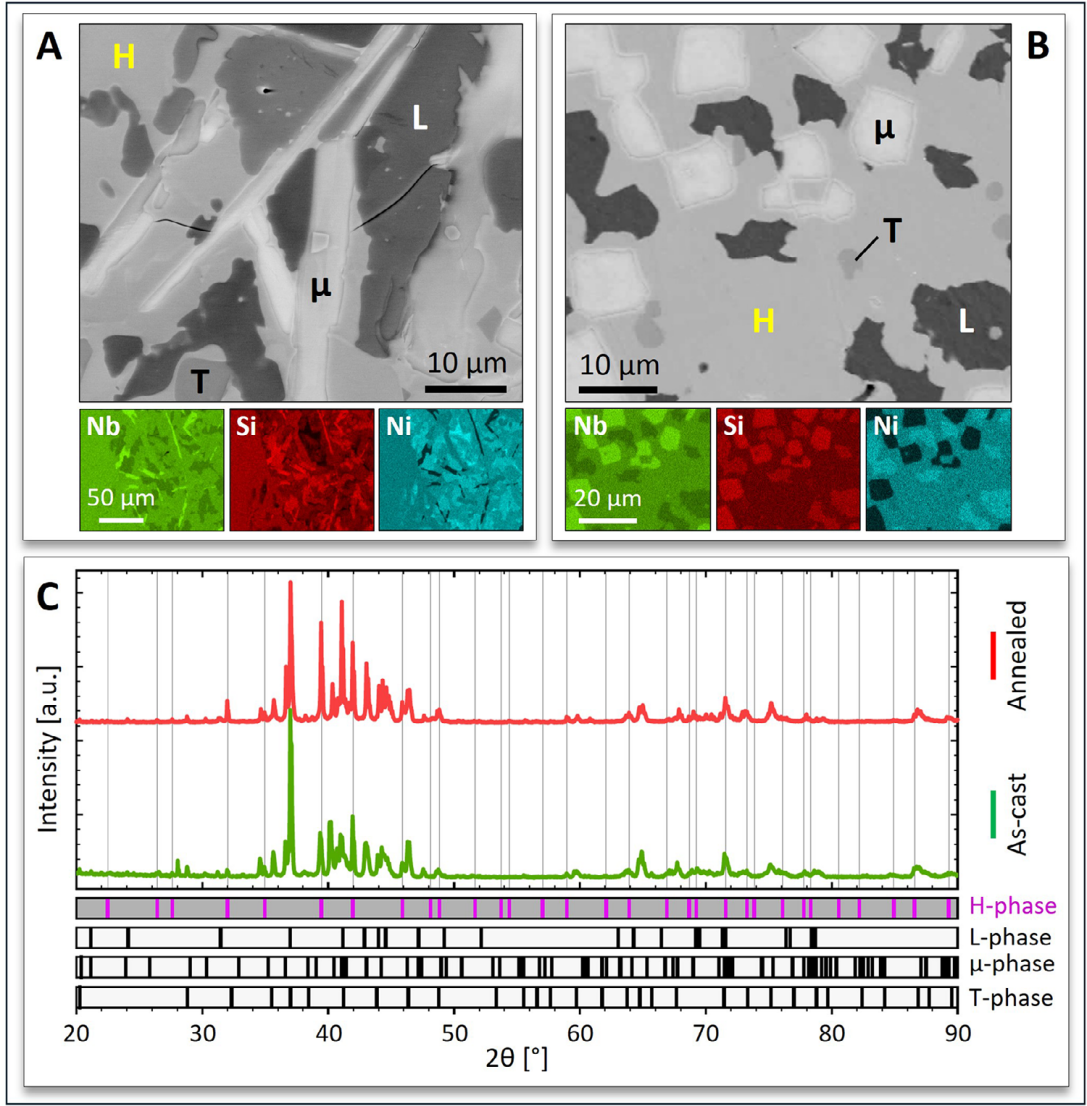
Characterization of arc‐melted Nb‐Si‐Ni alloy samples. SEM/EDS analysis of the A) as‐cast and B) annealed (1421 K, 336 h) samples shows four phases, i.e., H‐Nb_3_SiNi_2_, L‐Ni_3_SiNb_2_, µ‐Nb_7_Ni_6_, and T‐Nb_4_SiNi. C) XRD analysis of the as‐cast and annealed samples confirms the presence of the same four phases. The phase of interest, i.e., the Nb_3_SiNi_2_ ternary IMC (H‐phase), is highlighted in both SEM and XRD data using distinctive colors.

**Figure 2:**
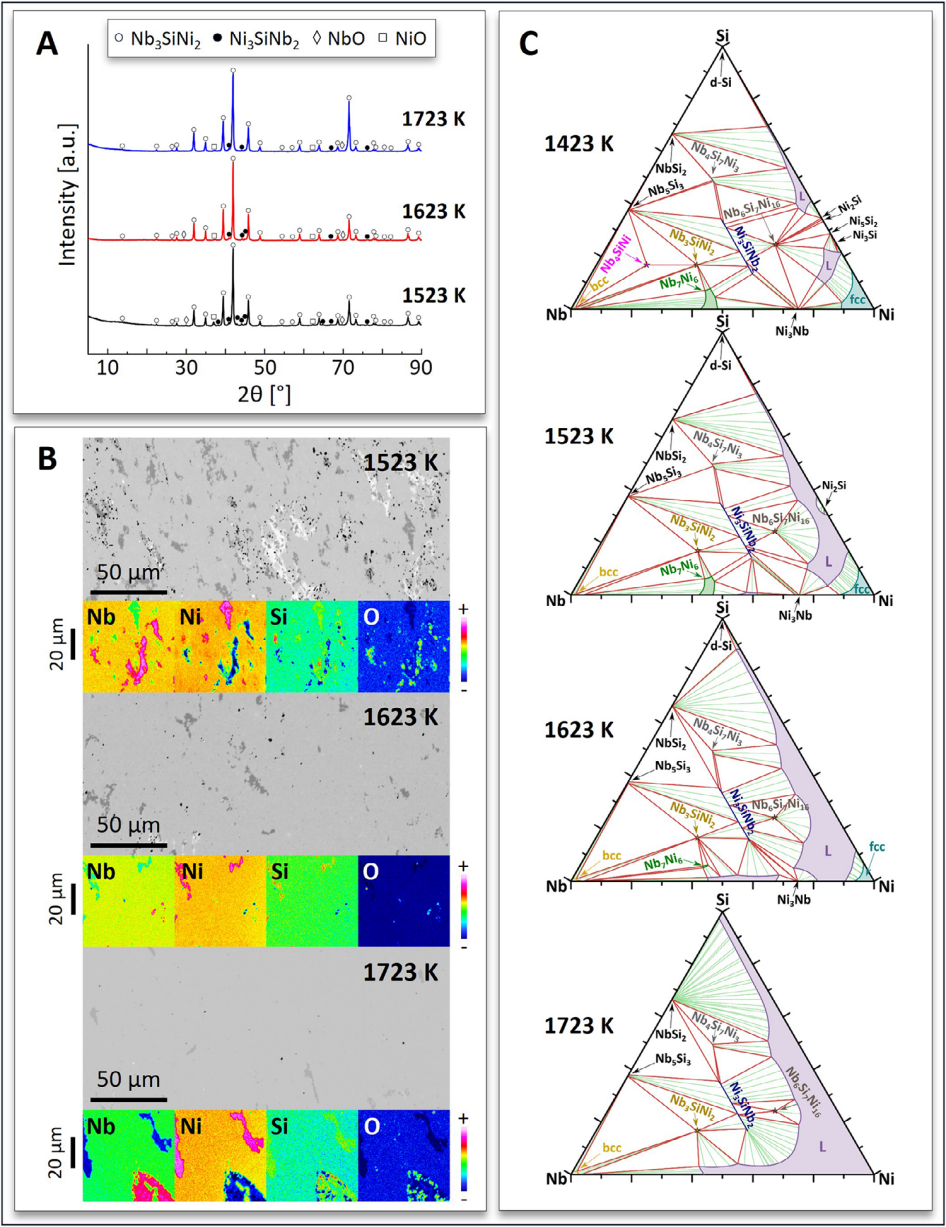
Characterization of the RHP Nb‐Si‐Ni alloy samples by means of A) XRD and B) EPMA/WDS shows the presence of mainly the two ZIP phases, i.e., H‐Nb_3_SiNi_2_ and L‐Ni_3_SiNb_2_, along with small amounts of oxidic inclusions (NbO_2_, NiO). Microstructural homogeneity and phase purity (in terms of the total ZIP phase content) increased with the sintering temperature. C) CALPHAD calculations of phase equilibria in the ternary Nb‐Si‐Ni system on the 1423, 1523, 1623, and 1723 K isotherms.

Arc melting was initially selected to experimentally synthesize bulk materials with the Nb_3_SiNi_2_ stoichiometry. The microstructural inspection of the as‐cast sample (**Figure** **1**A) by means of scanning electron microscopy (SEM) revealed the presence of four distinct phases, a finding supported by both energy‐dispersive X‐ray spectroscopy (EDS) and X‐ray diffraction (XRD). Phase identification by XRD (Figure 1C) revealed the co‐existence of the Nb_3_SiNi_2_ H‐phase (first candidate ZIP phase; *fcc* variant), the Nb_7_Ni_6_ µ‐phase, the Ni_3_SiNb_2_ ternary Laves (L) phase (first candidate ZIP phase; hexagonal variant), and the Nb_4_NiSi T‐phase. These four phases were peak‐indexed in accordance with data found in the ICSD database,^[^ ^48^ ^]^ whilst they were also identified as candidate phases during the experimental investigation of phase equilibria in the ternary Nb‐Si‐Ni system of refractory alloys.^[^ ^47^ ^]^ The nominal stoichiometries and measured EDS elemental compositions of the four phases in the as‐cast sample are provided in Table S1 (Supporting Information), which shows that all phases formed as off‐stoichiometric solid solutions during melt solidification rather than as strictly stoichiometric compounds. The formation of solid solutions in the Nb‐Si‐Ni system has been previously reported by dos Santos et al.,^[^ ^47^ ^]^ who provided strong evidence of solid solution formation associated with the dissolution of significant amounts of silicon (Si) in the Nb_7_Ni_6_ µ‐phase,^[^ ^47^ ^]^ as also indicated in Table S1 (Supporting Information). Even though arc melting aimed at the production of samples primarily made of the Nb_3_SiNi_2_ IMC, the as‐cast alloy was a mixture of four phases, i.e., the maximum number of phases in equilibrium for a ternary alloy at constant pressure according to Gibbs’ phase rule.^[^ ^49^ ^]^ In particular, the formation of the Nb‐rich Nb_4_NiSi T‐phase suggests the loss of substantial amounts of nickel (Ni) during arc melting, as Ni is volatile in vacuum above its melting point (1728 K). Considering the CALPHAD phase equilibria calculated in this work (**Figure** **2**C) in conjunction with the microstructure of the as‐cast alloy (Figure 1A), it is reasonable to assume that (rapid) melt solidification formed solid solutions of three principal phases, i.e., the Nb_3_SiNi_2_ H‐phase, the Nb_7_Ni_6_ µ‐phase, and the Ni_3_SiNb_2_ L‐phase, whereas the Ni‐impoverished residual melt yielded the Nb_4_NiSi T‐phase (see the 1423 K isotherm in Figure 2C). This hypothesis is supported by the small fraction of the Nb_4_NiSi T‐phase in the as‐cast alloy (Figure 1A).

A homogenization annealing of the as‐cast sample was performed at 1421 K to investigate whether the four‐phase assembly could change into an alloy with a higher Nb_3_SiNi_2_ IMC content. Annealing was also aimed at assessing the thermal stability of the Nb_3_SiNi_2_ phase of interest. The SEM/EDS/XRD analysis of the annealed alloy (Figure 1B,C) showed that it consisted of the same four phases, with a slightly increased fraction of the Nb_3_SiNi_2_ H‐phase that appears stable at these conditions; however, the marginal increase in Nb_3_SiNi_2_ fraction showed that the particular annealing treatment could not transform the as‐cast alloy into a material primarily made of Nb_3_SiNi_2_. This suggests that solid‐state elemental diffusion is not adequately fast at 1421 K to achieve alloy homogenization within 336 h, indicating the need for prolonged annealing treatments at higher temperatures. Moreover, the loss of Ni during arc melting – as confirmed by the presence of the Nb_4_NiSi T‐phase – is bound to prevent the formation of phase‐pure Nb_3_SiNi_2_, irrespective of the temperature and/or duration of the annealing treatment. Figure 1B shows only slight modifications in grain morphology due to annealing; for example, L‐Ni_3_SiNb_2_ and µ‐Nb_7_Ni_6_ grains appear more equiaxed than in the as‐cast sample. The elemental compositions of the four phases in the annealed sample were also determined by EDS (see Table S1, Supporting Information). Additional SEM micrographs of the arc‐melted (as‐cast & annealed) Nb‐Si‐Ni alloys are provided in Figure S1 (Supporting Information). The unsuccessful attempt to synthesize phase‐pure Nb_3_SiNi_2_ via arc melting showed that this processing route is difficult to master, mainly due to the poorly controlled losses of constituting elements above their melting points, T_m_ (i.e., T_m_(Ni) = 1728 K, T_m_(Nb) = 2750 K, T_m_(Si) = 1683 K). Moreover, calculations of the equilibrium phase mixtures in the Nb‐Si‐Ni ternary system at various temperatures are needed to better understand melt solidification, as prior studies examined only the 1073, 1323, and 1473 K isotherms.^[^ ^44^, ^45^, ^46^, ^47^ ^]^

The results of this work contradict the early work of Gladyshevskii et al.,^[^ ^44^ ^]^ who reported that melting a feedstock of elemental powders (Nb, Si, Ni) under an inert gas atmosphere (presumably, argon) produced quasi phase‐pure Nb_3_SiNi_2_ with only a small amount of a ternary Laves phase (possibly, the Ni_3_SiNb_2_ IMC also detected in this work). These authors used light optical microscopy to confirm the phase purity of their arc‐melted alloys, and powder XRD to propose the existence of a group of ternary IMCs with the H‐phase structure (space group *Fd* $\bar{3}$ *m*) and the R_3_SiNi_2_ general stoichiometry, with R being the early transition metals Mn, Cr, V, Nb, and Ta.^[^ ^44^ ^]^ Apart from demonstrating a much better command of the arc‐melting processing route, the 1964 pioneering work by Gladyshevskii et al.^[^ ^44^ ^]^ paved the way for the study of the ZIP phases introduced in this work. Similarly, the 1967 work by Jeitschko et al.^[^ ^7^ ^]^ opened the way for the discovery of the MAX phases family by Barsoum et al. almost 40 years later.^[^ ^5^, ^6^ ^]^

A thorough thermodynamic assessment of the ternary Nb‐Si‐Ni system was only recently conducted – 50 years after Gladyshevskii's pioneering work – in a set of works published by dos Santos et al.^[^ ^45^, ^46^, ^47^ ^]^ In an attempt to reproduce the results of Gladyshevskii et al.,^[^ ^44^ ^]^ dos Santos et al.^[^ ^46^ ^]^ synthesized twelve different Nb‐Si‐Ni alloy compositions via arc melting and subsequently homogenized the as‐cast samples at 1073 K for up to 1000 h. Curiously, the Nb_3_SiNi_2_ phase was not detected in any of the twelve homogenized samples characterized by means of SEM/EDS and XRD. The reason for this discrepancy between the results of Gladyshevskii et al.^[^ ^44^ ^]^ and dos Santos et al.^[^ ^46^ ^]^ is presently not understood. Based on their data, dos Santos et al. suggested a homogenization isothermal annealing at higher temperatures to explore the thermodynamic equilibria in this system.^[^ ^46^ ^]^ In a more extensive follow‐up study, dos Santos et al.^[^ ^47^ ^]^ investigated 48 alloy compositions in the Nb‐Si‐Ni system, implementing two different heat treatment approaches: (a) 1323 K for 336 h, and (b) 1473 K for 504 h. Using SEM/EDS and XRD after homogenization annealing, Nb_3_SiNi_2_ was only observed in seven samples, but none of these were phase‐pure Nb_3_SiNi_2_. All seven samples exhibited triphasic microstructures, each comprising two or three of the four principal phases also detected in this work, i.e., the ternary Laves, µ‐, T‐, and H‐phases; small fractions of the NbNi_3_ IMC or the unknown Z phase were also reported in three of the seven samples. The Z phase is one of three phases – tentatively designated X, Y, and Z – with unresolved crystal structures found by dos Santos et al.^[^ ^47^ ^]^ after annealing certain Nb‐Si‐Ni alloy compositions at 1473 K for 504 h.

#### 2.1.2 Synthesis of High Phase Purity Nb_3_SiNi_2_ by RHP

Careful consideration of the prior attempts by dos Santos et al.^[^ ^46^, ^47^ ^]^ and the findings of this work demonstrated that the synthesis of phase‐pure Nb_3_SiNi_2_ via arc melting is challenging. Even though Gladyshevskii et al.^[^ ^44^ ^]^ reported that the production of quasi phase‐pure Nb_3_SiNi_2_ is possible via the melting of the corresponding mixture of elemental powders in an inert gas atmosphere, high‐vacuum plasma arc melting methods have been empirically known to face challenges during the synthesis of compounds with strict stoichiometries,^[^ ^50^ ^]^ such as the ternary IMCs designated as the ZIP phases in this work, or the early transition metal ternary carbides/nitrides known as the MAX phases. As already mentioned, one important challenge is the evaporation of alloying elements in the vacuum chamber.^[^ ^51^ ^]^ Given the strict stoichiometry of the ZIP phases and the 312 MAX phases, the processing route and the starting powder feedstock must always be carefully adjusted to produce materials with high phase purity. For example, Goossens et al.^[^ ^52^ ^]^ explained only recently why using early transition metal hydride powders produces MAX phase‐based ceramics with higher phase purities than using elemental powders. These authors proved that the use of metal hydride powders lowers the formation temperature of MAX phase precursor phases (e.g., binary IMCs), increases the formation temperature of competing phases (e.g., binary carbides), and suppresses the oxidation of easily oxidizable powders (e.g., zirconium, Zr) by providing a reducing (H_2_‐rich) atmosphere created by powder dehydrogenation, thus allowing the use of finer powder feedstocks. Based on the above, it was decided in this work to employ reactive hot pressing (RHP) of carefully adjusted powder feedstocks consisting of hydride (NbH_0.89_) and elemental powders (Ni and Si) to produce phase‐pure Nb_3_SiNi_2_. The synthesis efforts were successful at three different sintering temperatures (i.e., 1523, 1623, and 1723 K), producing quasi phase‐pure Nb_3_SiNi_2_ with a small fraction of the ternary Ni_3_SiNb_2_ Laves phase, and traces of oxidic inclusions (NbO_2_ & NiO) that presumably stemmed from the limited oxidation of the raw powders. Electron probe microanalyzer (EPMA) images and wavelength‐dispersive X‐ray spectroscopy (WDS) elemental maps of all Nb_3_SiNi_2_‐based materials are shown in Figure 2B, together with their XRD patterns (Figure 2A). Additional EPMA/WDS data from all RHP quasi phase‐pure Nb_3_SiNi_2_ materials are shown in Figure S2 (Supporting Information). The XRD pattern of the pulverized RHP sample sintered at 1723 K was Rietveld‐refined (Figure S3 and Table S2, Supporting Information).

Figure 2C shows CALPHAD calculations of phase equilibria in the ternary Nb‐Si‐Ni system at 1423 K (i.e., close to the temperature of isothermal annealing, 1421 K, of the arc‐melted alloy), 1523, 1623, and 1723 K. These calculations suggest that melt solidification during vacuum arc melting yielded a four‐phase mixture comprising the L‐Ni_3_SiNb_2_ line compound, the H‐Nb_3_SiNi_2_ point compound (i.e., the targeted IMC), and the µ‐Nb_7_Ni_6_ phase‐field compound with enhanced Si solid solubility. Due to the anticipated loss of Ni during vacuum arc melting, a small fraction of the melt was Ni‐poor and solidified as the T‐Nb_4_SiNi point compound, which appears only in the 1423 K isotherm (Figure 2C, top). On the other hand, all three alloys synthesized via RHP at 1523/1623/1723 K resulted in quasi binary phase mixtures made of the H‐Nb_3_SiNi_2_ and L‐Ni_3_SiNb_2_ IMCs. The NbO_2_ and NiO oxide impurities present in the RHP alloys were not encountered in the arc‐melted alloys due to the much higher processing temperatures associated with the complete melting of all raw elemental powders and their oxide films during arc melting synthesis.

### 2.2 Characterization of ZIP Phases in the Nb‐Si‐Ni System

#### 2.2.1 Crystal Structure Determination by TEM – The ZIP Phase Variants

Even though the arc‐melted sample had a small Nb_3_SiNi_2_ fraction, focused ion beam (FIB) was used to lift out thin foils from a large Nb_3_SiNi_2_ grain in the annealed sample (Figure S4A, Supporting Information) to study its crystal structure by means of scanning transmission electron microscopy (STEM) and selected area diffraction (SAED). STEM/EDS analysis of this grain revealed a composition close to the Nb_3_SiNi_2_ stoichiometry, with limited deviations from the nominal Ni (5 at.% excess) and Si (5 at.% deficiency) contents (Figure S4B, Supporting Information). Atomically resolved BF‐STEM images of the same grain revealed a “zigzag” atomic arrangement, observed in low‐index crystallographic orientations (Figure S5A,B,C, Supporting Information). Using published crystallographic data (CIF file ICSD‐2044323)^[^ ^44^ ^]^ and the SingleCrystal software package,^[^ ^53^ ^]^ a kinematic simulation of a selected area diffraction pattern (SAEDP) in the low‐index [$\bar{1}$12] zone axis (Z.A.) was compared with the recorded SAEDP (Figure S5B,D, Supporting Information). The excellent agreement between experimental and simulated SAEDPs confirmed that the crystal structure of Nb_3_SiNi_2_ is diamond cubic (*Fd* $\bar{3}$ *m*, space group, SG, 227), as reported by Gladyshevskii et al.^[^ ^44^ ^]^

**Figure 3:**
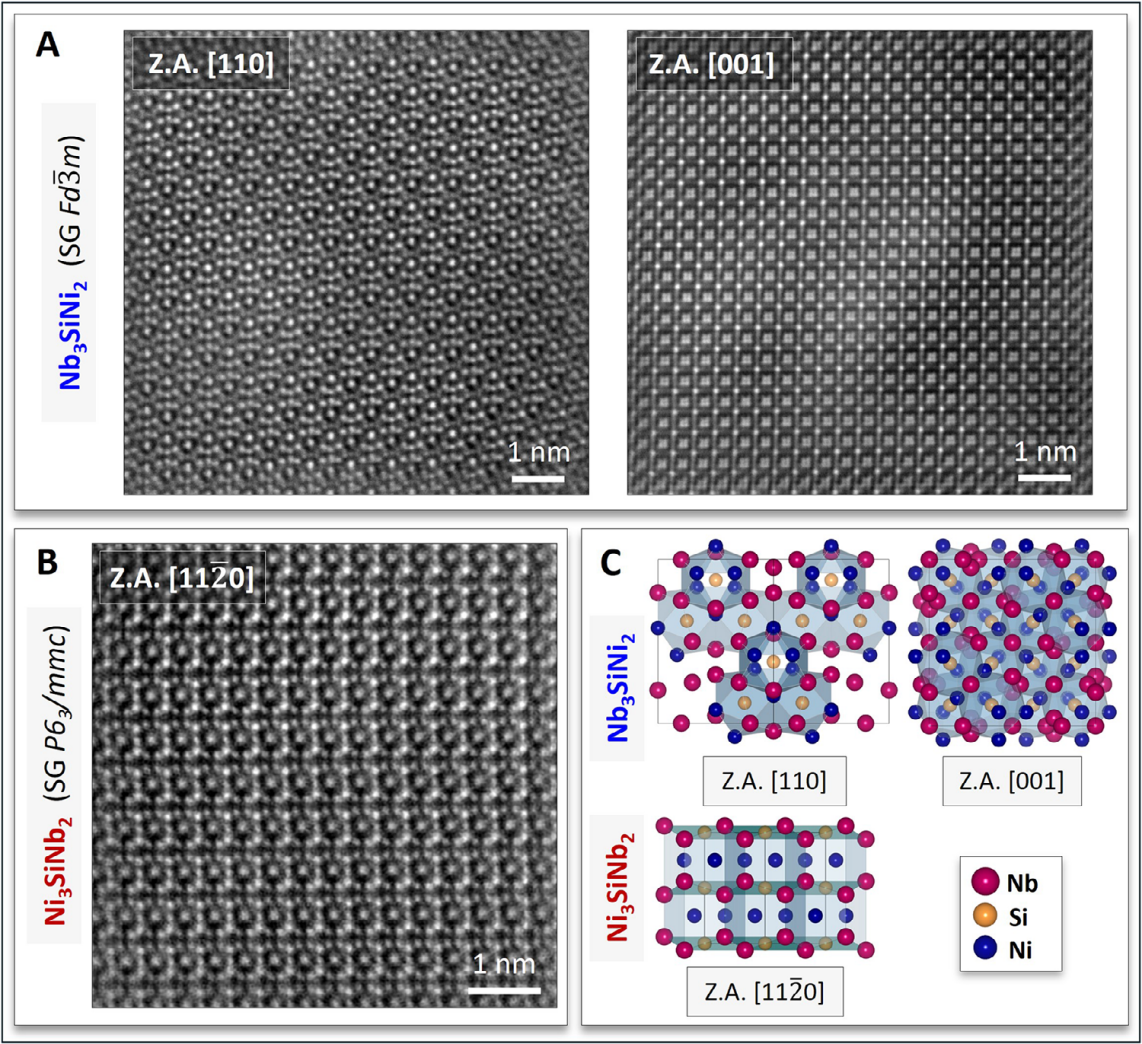
Atomically resolved HAADF STEM images of A) Nb_3_SiNi_2_ (*fcc*, SG *Fd* $\bar{3}$ *m*) and B) Ni_3_SiNb_2_ (hexagonal, SG *P6_3_/mmc*). A) Z.A. [110] (left) and [001] (right) of Nb_3_SiNi_2_. B) Z.A. [11$\bar{2}$0] of Ni_3_SiNb_2_. C) Schematics depicting the [110] and [001] Z.A. of Nb_3_SiNi_2_ and the [11$\bar{2}$0] Z.A. of Ni_3_SiNb_2_; these schematics were made with the VESTA software.^[^ ^54^ ^]^

The successful RHP synthesis of quasi phase‐pure Nb‐Si‐Ni alloy samples allowed the study of FIB foils lifted out from large grains of Nb_3_SiNi_2_ and Ni_3_SiNb_2_, i.e., the two main phases (Figure 2A,B), by means of high‐angle annular dark‐field (HAADF) STEM, using a double Cs‐corrected FEI Titan^3^ 60–300. Atomically resolved HAADF STEM images of both phases are shown in **Figure** **3**. The crystal structure of Nb_3_SiNi_2_ (Figure 3A) was again identified as *fcc* (*Fd* $\bar{3}$ *m*, SG 227), whereas that of Ni_3_SiNb_2_ (Figure 3B) was found to be hexagonal (*P6_3_/mmc*, SG 194). Figure 3C shows the schematic representations of the same Z.A. of both phases depicted in Figure 3A,B. The two variants of the ternary Nb‐Si‐Ni IMCs, i.e., the *fcc* Nb_3_SiNi_2_ and the hexagonal Ni_3_SiNb_2_, are considered from this point onward the two variants of the Nb‐Si‐Ni ZIP phases. It will be further demonstrated that the ZIP phase concept of ternary IMCs extends beyond the Nb‐Si‐Ni system, thus constituting a new family of “nanostructured materials”, in alignment with the prediction of Gleiter.^[^ ^2^ ^]^

**Figure 4:**
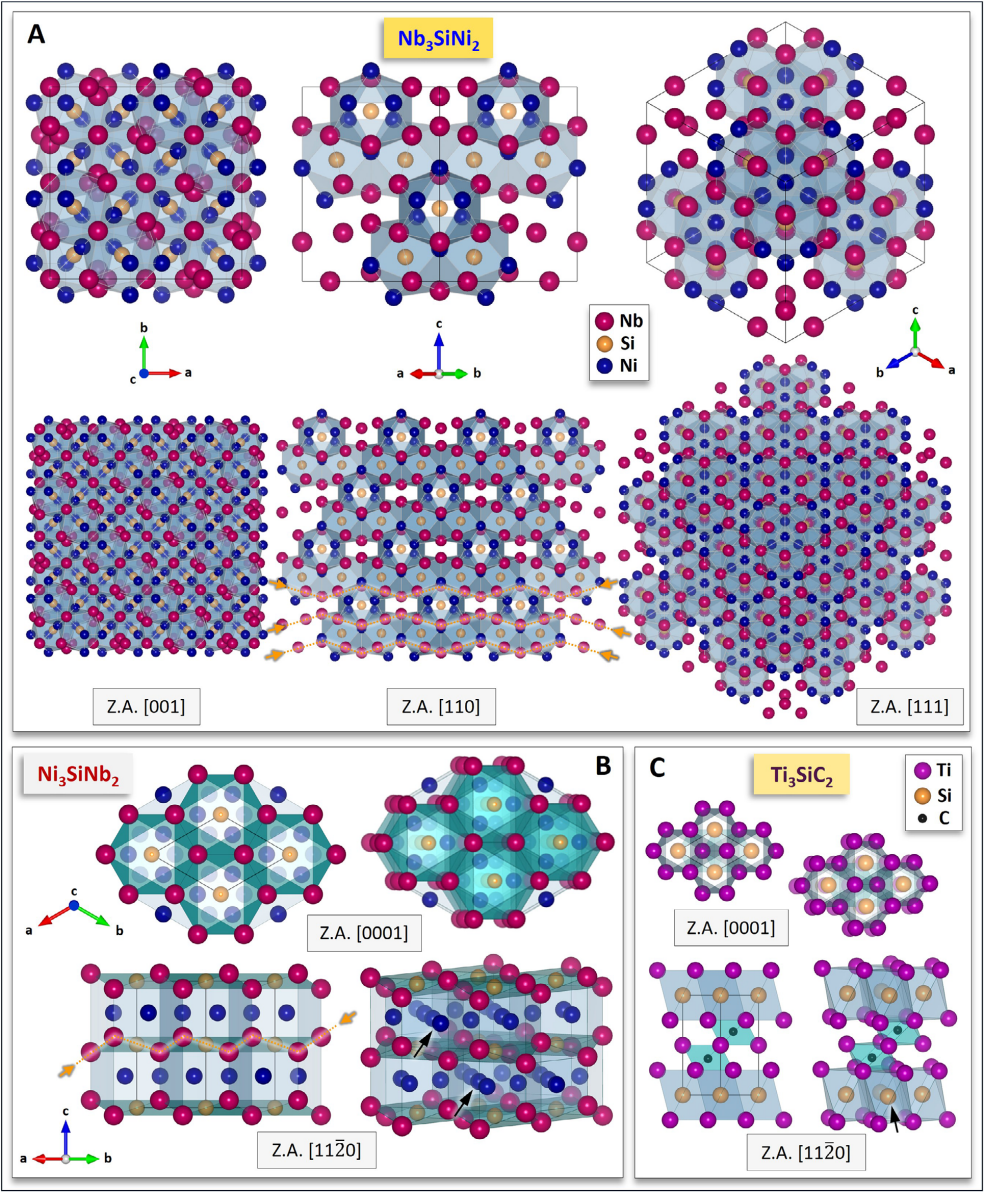
A) Schematics of three Z.A. of the *fcc* Nb_3_SiNi_2_ ZIP phase: [001], [110], and [111]. The confines of one unit cell (96 atoms) are outlined using thicker black lines (top row). Representation of the Nb_3_SiNi_2_ crystal structure resulting from the stacking of 2 × 2 × 2 unit cells along the [001], [110], and [111] Z.A. (bottom row). B) Schematics of two Z.A. of the hexagonal Ni_3_SiNb_2_ ZIP phase: [0001] and [11$\bar{2}$0] (left half of the image); the right half of the image shows perspectives of the same Z.A. after a minor counterclockwise rotation. The confines of the unit cell (12 atoms) are outlined by thicker black lines. C) Schematics of two Z.A. of the hexagonal Ti_3_SiC_2_ MAX phase: [0001] and [11$\bar{2}$0] (left half of the image) and 3D perspectives thereof after a minor counterclockwise rotation (right half of the image). The confines of one unit cell (6 atoms) are outlined using thicker black lines. In the 3D perspectives of the [11$\bar{2}$0] Z.A. of the Ni_3_SiNb_2_ ZIP phase and the Ti_3_SiC_2_ MAX phase, black arrows pinpoint the atomic layers, the removal of which is likely to yield 2D derivatives. Dashed orange lines and arrows are combined to highlight atomic zigzagging in both *fcc* Nb_3_SiNi_2_ (Z.A. [110]) and hexagonal Ni_3_SiNb_2_ (Z.A. [11$\bar{2}$0]) ZIP phases.

Version 3.5.8 of the VESTA software^[^ ^54^ ^]^ was utilized to depict different Z.A. in the Nb_3_SiNi_2_ and Ni_3_SiNb_2_ ZIP phase variants, thus illustrating the complexity and size of their unit cells, as well as the atomic “zigzagging” inferred by the herein introduced term “ZIP phases”. **Figure** **4** shows the [001], [110], and [111] Z.A. of Nb_3_SiNi_2_ and the [0001] and [11$\bar{2}$0] Z.A. of Ni_3_SiNb_2_. It also depicts the [0001] and [11$\bar{2}$0] Z.A. of the well‐known Ti_3_SiC_2_ MAX phase to compare its crystal structure complexity with that of the hexagonal Ni_3_SiNb_2_ ZIP phase variant; direct size comparison is possible as both Ti_3_SiC_2_ and Ni_3_SiNb_2_ contain Si.

Figure 4 illustrates the remarkable differences in crystal structure complexity and size of unit cell for the *fcc* Nb_3_SiNi_2_ ZIP phase (96 atoms per unit cell; Figure 4A), the hexagonal Ni_3_SiNb_2_ ZIP phase (12 atoms per unit cell; Figure 4B), and the hexagonal Ti_3_SiC_2_ MAX phase (6 atoms per unit cell; Figure 4C). Unit cell volume, theoretical density, and Wyckoff parameters for Ti_3_SiC_2_, Nb_3_SiNi_2_, and Ni_3_SiNb_2_ are compared in Table S3 (Supporting Information). All three nanostructured solids are characterized by centrosymmetric Bravais lattices, i.e., the Nb_3_SiNi_2_ ZIP phase is *fcc* (*Fd* $\bar{3}$ *m*, SG 227), whereas the Ni_3_SiNb_2_ ZIP phase and the Ti_3_SiC_2_ MAX phase are both hexagonal (*P6_3_/mmc*, SG 194); it appears that these particular Bravais lattices favor the formation of nanostructured solids (i.e., ternary ceramics & ternary IMCs) with characteristic atomic zigzagging.

#### 2.2.2 Compositional Reconstruction of the Nb‐Si‐Ni ZIP Phase Variants by APT

**Figure 5:**
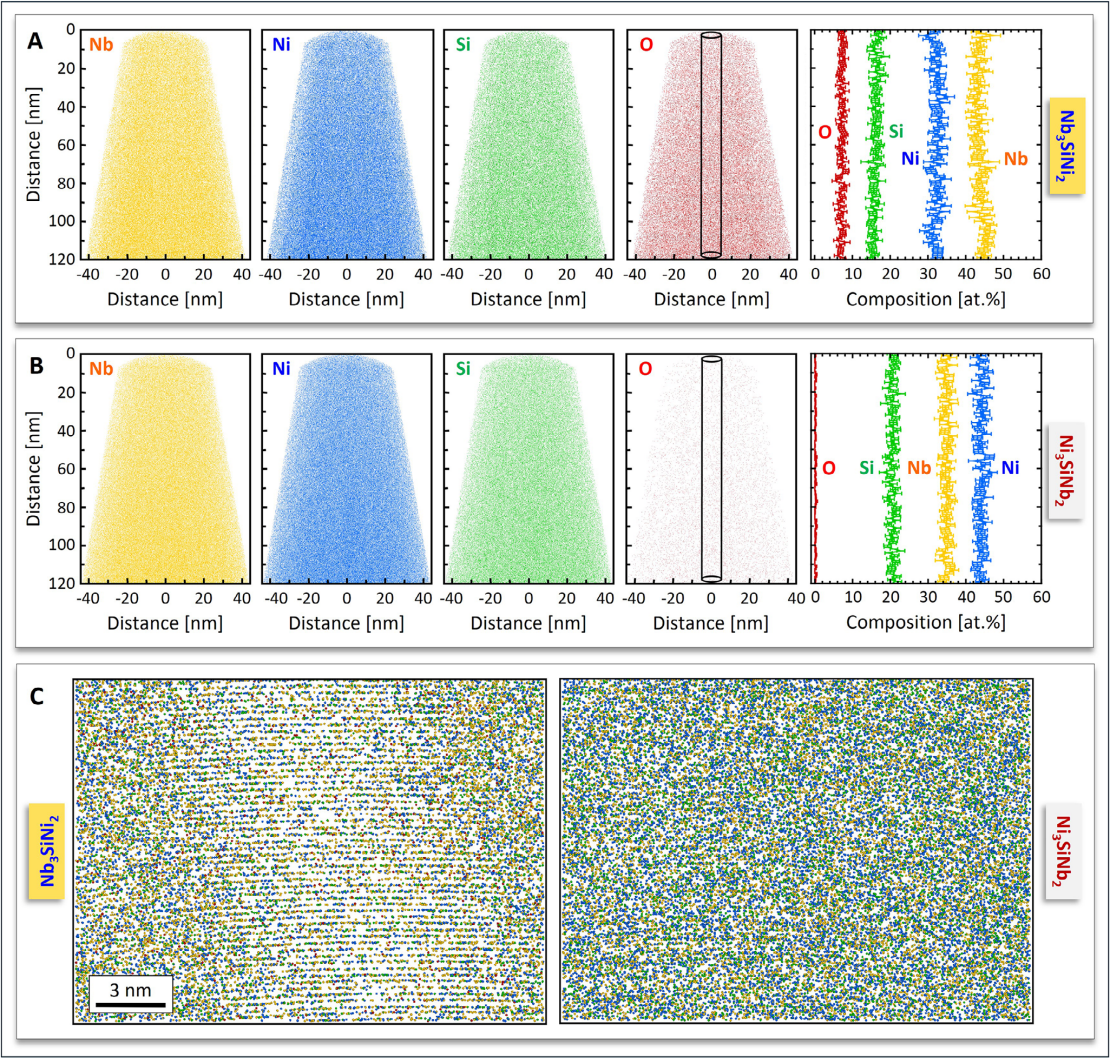
Spatially resolved composition of the two variants of the Nb‐Si‐Ni ZIP phases, as determined by APT. Reconstructions of the Nb, Ni, Si, and O atomic positions and elemental profiles for Nb_3_SiNi_2_ A) and Ni_3_SiNb_2_ B). The elemental profiles stem from cylinders (∅ 10 nm) as shown in the maps of the O atomic positions. C) Atomic positions within a rectangle (20 × 20 × 15 nm^3^) for Nb_3_SiNi_2_ and Ni_3_SiNb_2_. The mass spectra of Nb_3_SiNi_2_ and Ni_3_SiNb_2_ are found in Figures S7 and S8 (Supporting Information), respectively.

The compositions of the Nb_3_SiNi_2_ and Ni_3_SiNb_2_ ZIP phase variants were spatially resolved by means of atom probe tomography (APT). Details of the site‐specific FIB lift‐outs can be found in Figure S6 (Supporting Information). Reconstructions of the Nb, Ni, Si, and O atomic positions, as well as the composition profiles of Nb_3_SiNi_2_ and Ni_3_SiNb_2_ are presented in **Figure** **5**A,B. Apart from the principal Nb, Ni, and Si elements, O, C, Ta, and W impurities were also identified, and the mass spectra are shown in Figure S7 (Supporting Information) for Nb_3_SiNi_2_ and Figure S8 (Supporting Information) for Ni_3_SiNb_2_. The sum of C, Ta, and W impurities was <1 at.% in both phases. The average composition of Nb_3_SiNi_2_ was 45 ± 1 at.% Nb, 16 ± 1 at.% Si, 32 ± 1 at.% Ni and 7 ± 1 at.% O (Figure 5A). The O impurities were tentatively associated with Nb, as a significant fraction of molecular NbO ions is evident in the mass spectrum of Figure S7 (Supporting Information). Neglecting the O impurities, a Nb_2.8_SiNi_2.0_ stoichiometry was obtained, in excellent agreement with the nominal composition of Nb_3_SiNi_2_. The average composition of Ni_3_SiNb_2_ was 35±1 at.% Nb, 21 ± 1 at.% Si and 44 ± 1 at.% Ni, whilst the O content was <1 at.% (Figure 5B), corresponding to a stoichiometry of Ni_2.1_SiNb_1.7_. Magnifications of the atomic positions in the Nb_3_SiNi_2_ and Ni_3_SiNi_2_ ZIP phases in a rectangular volume of 20 × 20 × 15 nm^3^ are shown in Figure 5C. These data provide evidence of atomic “layering” in Nb_3_SiNi_2_, whereas in the case of Ni_3_SiNb_2_, the experimental proof of nanolamination (see Figure 4B) is probably at the resolution limit of APT. The O content data shown in Figure 5A,B are supported by data collected from a third sample that contained both Ni_3_SiNb_2_ and Nb_3_SiNi_2_ grains separated by a slanted grain boundary, GB (Figure S9, Supporting Information). The APT data in Figure S9 (Supporting Information) show the presence of O in the Nb_3_SiNi_2_ grain and its absence from the neighboring Ni_3_SiNb_2_ grain. The better accommodation of O in Nb_3_SiNi_2_ suggests that the O solid solubility in the *fcc* ZIP phase variant is higher than that in the Ni_3_SiNb_2_ hexagonal ZIP phase variant. The O impurities measured by APT cannot, thus, be only attributed to the oxidation of Nb, as Nb is strongly present in both ZIP phase variants. The reason for the difference in O content might be sought in the size and “open spaces” available in the unit cells of the two ZIP phase variants (Figure 4). The hypothesis regarding O solubility has been tested with ZIP phases in other ternary systems explored in this work, i.e., Nb‐Si‐Co, Ta‐Si‐Ni, V‐Si‐Ni, and Nb‐Si‐Fe.

#### 2.2.3 Thermal, Electrical, and Magnetic Properties

**Figure 6:**
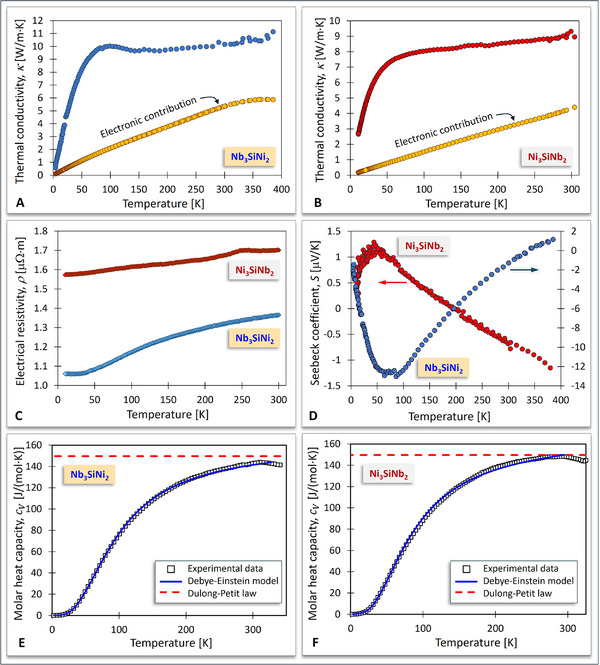
Temperature dependence of the thermal conductivity, *κ*, for Nb_3_SiNi_2_ A) and Ni_3_SiNb_2_ B). The electronic contribution to the thermal conductivity for both ZIP phases has been calculated using the Wiedemann‐Franz law. C) Temperature dependence of the electrical resistivity, *ρ*, for Nb_3_SiNi_2_ and Ni_3_SiNb_2_. D) Temperature dependence of the Seebeck coefficient, *S*, for Nb_3_SiNi_2_ and Ni_3_SiNb_2_. Temperature dependence of the molar heat capacity, *c_V_*, for Nb_3_SiNi_2_ E) and Ni_3_SiNb_2_ F). A Debye‐Einstein model (Equation 1 in Section "Thermal, Electrical, and Magnetic Properties") shown as solid blue lines, was fitted to the experimental *c_V_* data (squares). The dashed red lines correspond to the upper *c_V_* limit, dictated by the Dulong‐Petit law.

Thermal, electrical, and magnetic properties of the Nb_3_SiNi_2_ and Ni_3_SiNb_2_ ZIP phases have been determined according to the experimental methodologies described in Section “Thermal, Electrical, and Magnetic Properties”. **Figure** **6**A,B shows the variation of the thermal conductivity, *κ*, of Nb_3_SiNi_2_ (Figure 6A) and Ni_3_SiNb_2_ (Figure 6B) as a function of temperature. The electronic contribution to the total thermal conductivity was estimated based on the Wiedemann‐Franz law to be approximately 50% of the total thermal conductivity of Nb_3_SiNi_2_ and Ni_3_SiNb_2_ at room temperature (RT); this is much higher than the electronic contribution to the total thermal conductivities of the MAX phases, which has been calculated to vary in the 10–20% range.

Figure 6C shows the variation of the electrical resistivity, *ρ*, of Nb_3_SiNi_2_ and Ni_3_SiNb_2_ as a function of temperature. Both Nb_3_SiNi_2_ and Ni_3_SiNb_2_ exhibit metallic behavior, showing a reduction in electrical resistivity with decreasing temperature. The residual resistivity (≈1 µΩ cm) is reached at around 35 K for Nb_3_SiNi_2_; this resistivity is associated with the scattering of the charge carrier by defects and/or impurities in this sample. On the other hand, the residual resistivity of Ni_3_SiNb_2_ does not seem to be reached at 10 K, which suggests that less defects were present in this sample.

Figure 6D shows the variation of the Seebeck coefficient, *S*, of Nb_3_SiNi_2_ and Ni_3_SiNb_2_ as a function of temperature. The Seebeck coefficients are radically different for these two ZIP phases. First, their signs are opposite, i.e., positive for Nb_3_SiNi_2_ and negative for Ni_3_SiNb_2_ at high temperatures. Second, the sign of the Seebeck coefficient changes at ≈340 K and ≈190 K for Nb_3_SiNi_2_ and Ni_3_SiNb_2_, respectively. A sign change in the Seebeck coefficient implies that the band structures are probably not parabolic, and that a two‐band model is likely necessary to account for the transport properties of the Nb_3_SiNi_2_ and Ni_3_SiNb_2_ ZIP phases. Magnetoresistance initially measured at 10 K and 9 T was less than 0.15%.

Molar heat capacity, *c_V_*, measured as a function of temperature (2‐325 K range) showed that the recorded curves for both Nb_3_SiNi_2_ and Ni_3_SiNb_2_ exhibited an asymptotic behavior, i.e., the *c_V_* values approached asymptotically the value of ∼146 ± 3 J/(mol K) (Figure 6E,F). This agrees with the Dulong‐Petit law, which states that at T → ∞, *c_V_* = 3NR ≈ 149.7 J/(mol K), where N is the number of atoms per formula unit (N = 6) and R = 8.314 J/(mol K). Fitting a Debye‐Einstein model^[^ ^55^, ^56^ ^]^ (Equation 1 in Section “Thermal, Electrical, and Magnetic Properties”) to these profiles yields the Debye, θ_*D*_, and Einstein, θ_*E*_, temperatures, as well as the Sommerfeld coefficient, *γ*, for each sample (**Table** **1**). The measured molar heat capacities and fitted model for the Ni_3_SiNb_2_ and Nb_3_SiNi_2_ samples are shown in Figure 6E,F.

**Table 1:** Fitted Debye (*θ_D_*) and Einstein (*θ_E_*) temperatures for Nb_3_SiNi_2_ and Ni_3_SiNb_2_, using the Debye‐Einstein model (Equation 1 in Section "Thermal, Electrical, and Magnetic Properties"). The Sommerfeld coefficient, *γ*, and the number of Einstein nodes, *α*, are also given. HC is the molar heat capacity in J/(mol K).

ZIP phase	HC at RT [J/mol K]	*γ* [J/mol K^2^]	θ_*E*_ [K]	θ_*D*_ [K]	*α*
Nb_3_SiNi_2_	143	15 ± 1	192 ± 2	426 ± 2	0.19 ± 0.02
Ni_3_SiNb_2_	148	35 ± 1	183 ± 3	377 ± 5	0.14 ± 0.01

**Figure 7:**
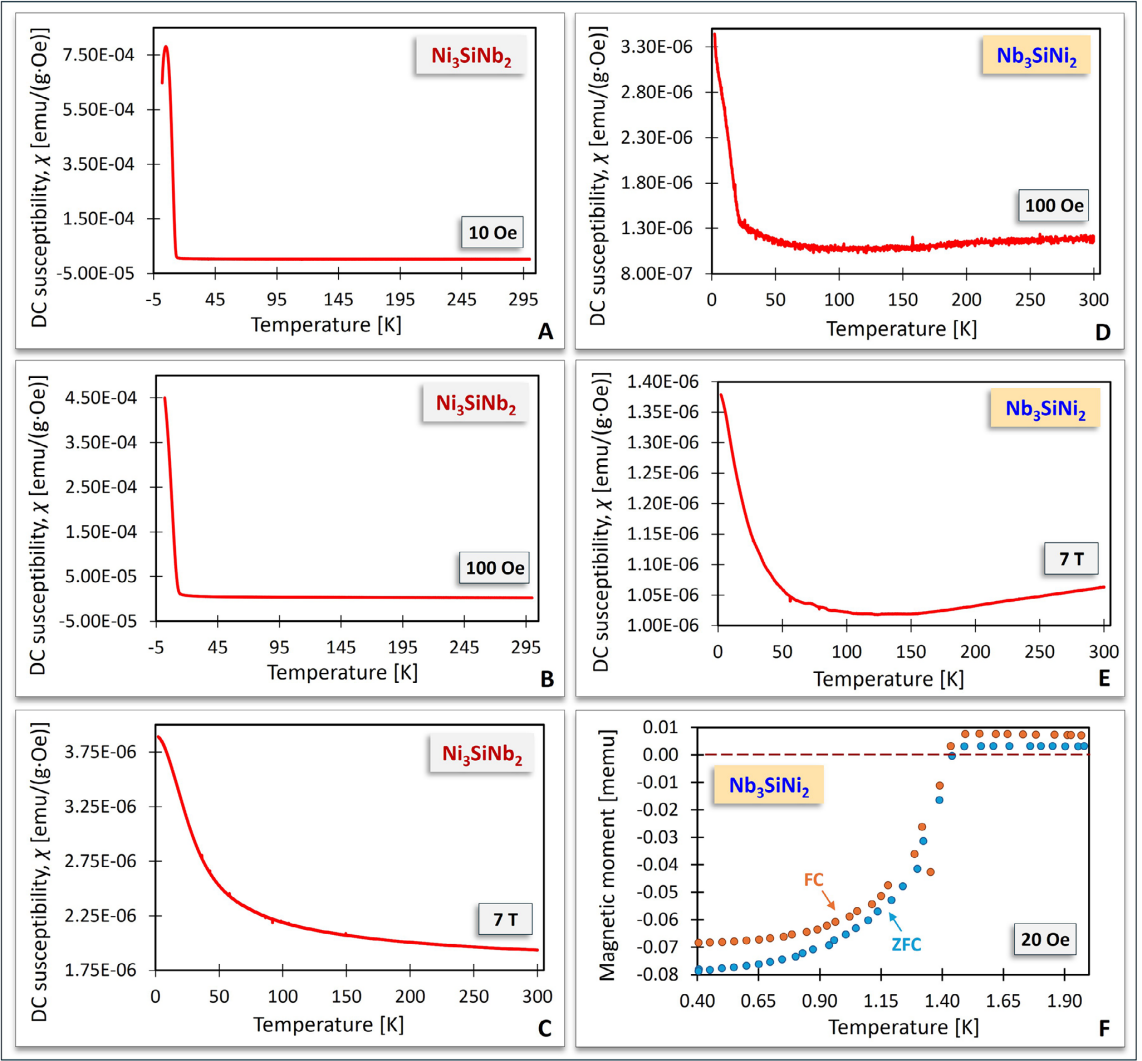
Temperature‐dependent DC magnetization susceptibility, *χ*(*T*), at applied magnetic fields of A) 10 Oe, B) 100 Oe, and C) 7 T for Ni_3_SiNb_2_; and D) 100 Oe and E) 7 T for Nb_3_SiNi_2_. F) At an applied magnetic field of 20 Oe, the negative magnetization of Nb_3_SiNi_2_ below 1.4 K is a proof of superconductivity; (Z)FC = (zero)‐field cooling.

Temperature‐dependent DC magnetization susceptibility, *χ*(*T*), curves were recorded as a function of the applied magnetic field for Ni_3_SiNb_2_ and Nb_3_SiNi_2_ (**Figure** **7**). As may be seen, Ni_3_SiNb_2_ was tested in the low magnetic fields of 10 Oe (Figure 7A) and 100 Oe (Figure 7B) as well as in the high magnetic field of 7 T (Figure 7C), whereas Nb_3_SiNi_2_ was tested in the low magnetic field of 100 Oe (Figure 7D) and the high magnetic field of 7 T (Figure 7E). Figure 7A,B shows a sudden change in the measured *χ*(*T*) of Ni_3_SiNb_2_ at low magnetic fields, indicating a transition from ferromagnetic (FM) to paramagnetic (PM) behavior with a Curie temperature, *T_C_*, of ≈14 K. At the weak magnetic field of 10 Oe, *χ*(*T*) decreases sharply below ≈5 K, whilst at the strong magnetic field of 7 T (Figure 7C), Ni_3_SiNb_2_ remains paramagnetic within the explored temperature range. Similarly, the measured *χ*(*T*) of Nb_3_SiNi_2_ at the weak magnetic field of 100 Oe (Figure 7D) indicates that the FM‐PM transition occurs at *T_C_* ≈ 22 K, whereas at the strong magnetic field of 7 T, the sample remains paramagnetic within the measured temperature range.

**Figure 8:**
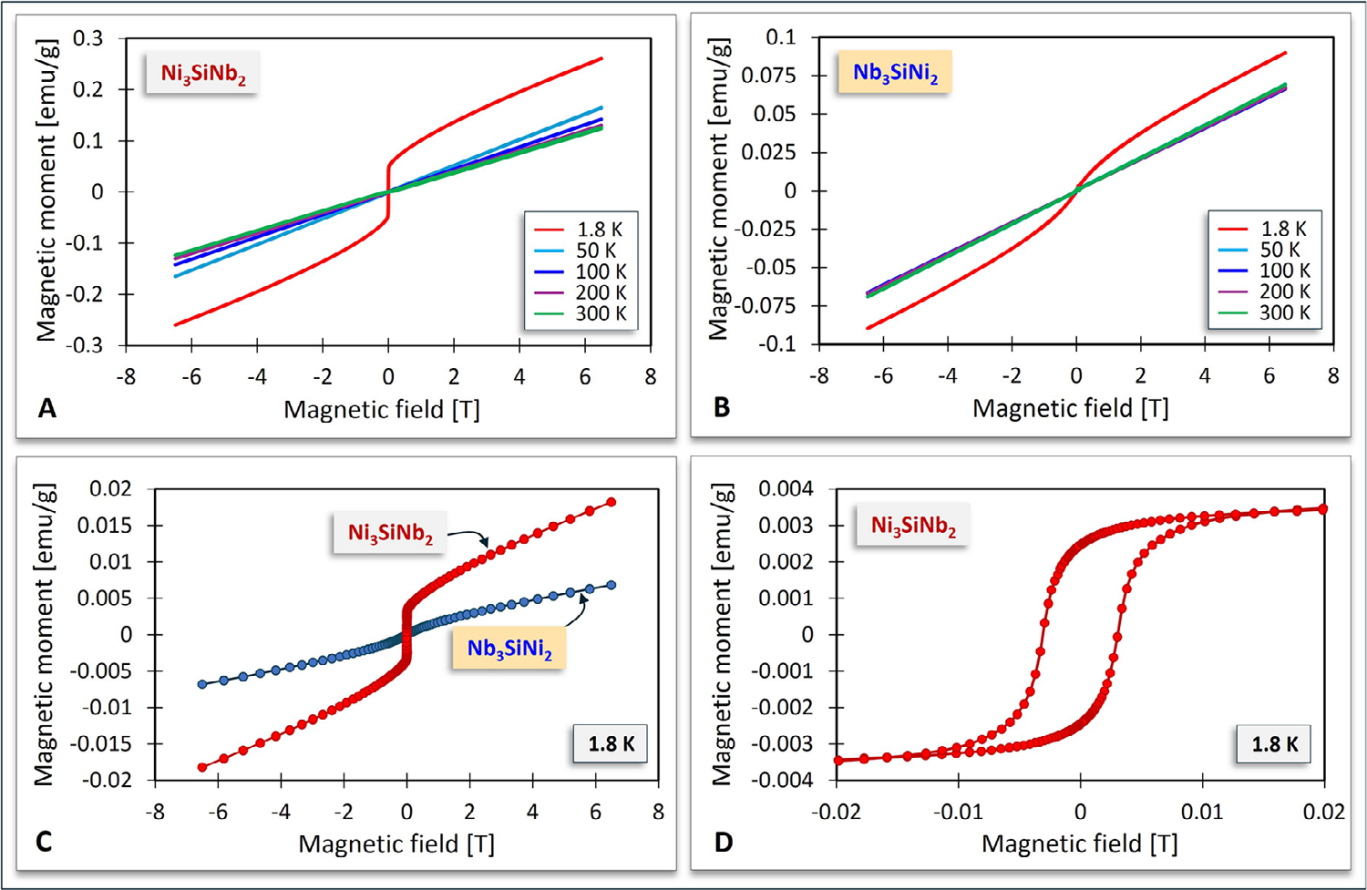
Magnetic field‐dependent magnetization of Ni_3_SiNb_2_ A) and Nb_3_SiNi_2_ B) at 1.8, 50, 100, 200, and 300 K. The *m*(*H*) curves of both Ni_3_SiNb_2_ and Nb_3_SiNi_2_ at 1.8 K show ferromagnetic behavior C) with a pronounced hysteresis loop for Ni_3_SiNb_2_ D).

As expected, magnetic field‐dependent magnetization curves, *m*(*H*), measured below the Curie temperature, *T_C_*, for both Ni_3_SiNb_2_ (**Figure** **8**A) and Nb_3_SiNi_2_ (Figure 8B) showed clear ferromagnetic behavior. As shown in Figure 8D, the Ni_3_SiNb_2_ ZIP phase exhibits a clear hysteresis loop at 1.8 K, whereas the coercive field in the *m*(*H*) curve of the Nb_3_SiNi_2_ ZIP phase at 1.8 K is negligible (Figure 8C).

Interestingly, magnetization measurements performed on the Nb_3_SiNi_2_ ZIP phase using a He3 refrigerator under both FC (field cooling) and ZFC (zero‐field cooling) conditions (Figure 7F) captured clear evidence of superconductivity with a critical temperature, *T_c_*, of 1.4 K. Even though it is difficult to estimate the volume fraction of superconductivity in powder samples similar to the ones used in this study, the near overlap of the FC and ZFC curves in Figure 7F suggests that it is close to 100%.

#### 2.2.4 Other Properties

The Archimedes density of the Nb_3_SiNi_2_‐based material was measured to be 7.630 g cm^−3^, whilst that of the Ni_3_SiNb_2_‐based material was measured to be 7.787 g cm^−3^.

The Vickers hardness, *H_V_*, of the quasi phase‐pure Nb_3_SiNi_2_ material was measured by means of indentation to be *H_V_* = 12.6 ± 0.1 GPa.

The flexural strength, σ_*f*_, of the quasi phase‐pure Nb_3_SiNi_2_ material was measured by means of four‐point bending (4 PB) tests to be σ_*f*_ = 251 ± 10 MPa.

The dynamic Young's modulus, *E*, of the quasi phase‐pure Nb_3_SiNi_2_ material was determined by the impulse excitation technique (IET) to be *E* = 253.3 ± 0.4 GPa. Moreover, the damping or internal friction, *Q* ^−1^, of the same material was determined by means of IET to be *Q* ^−1^ = 0.0033 ± 0.0002%, which corresponds to a characteristic resonance frequency of 7224 ± 6 Hz.

#### 2.2.5 Stability of Nb‐Si‐Ni ZIP Phases and their Binary/2D Derivatives by DFT

**Figure 9:**
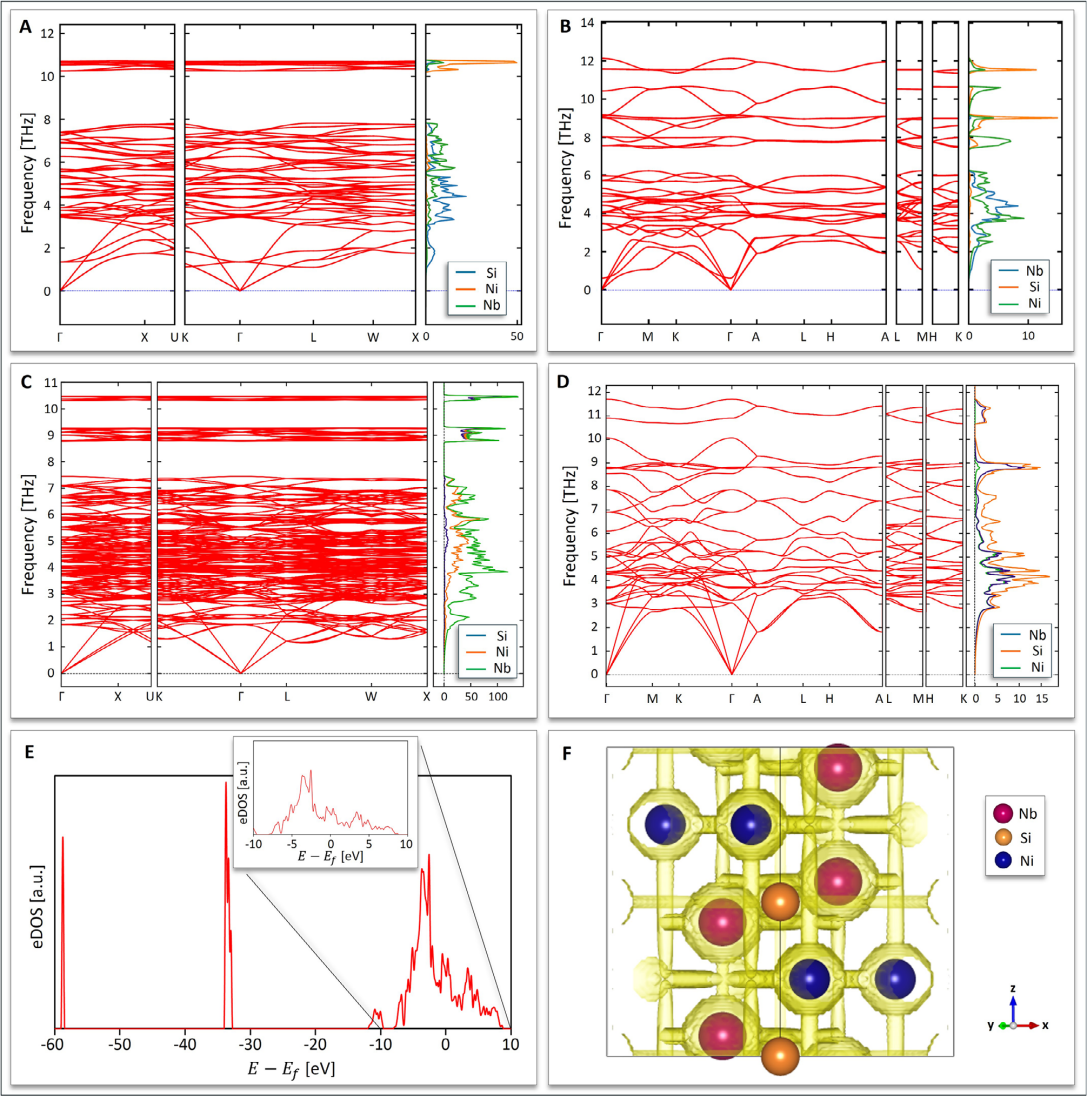
Predicted phonon dispersion and atom‐projected phonon density of states (PDOS) without the inclusion of temperature effects (i.e., at 0 K) A,B) and at 1723 K C,D) for the stoichiometric *fcc* Nb_3_SiNi_2_ A,C) and hexagonal Ni_3_SiNb_2_ B,D) ZIP phase variants. E) The electronic density of states (eDOS) in hexagonal Ni_3_SiNb_2_, using HSE06 functional (the Fermi energy is at 0 eV). F) Reduced density gradient map of hexagonal Ni_3_SiNb_2_.

The phonon band structures of Nb_3_SiNi_2_ (**Figure** **9**A) and Ni_3_SiNb_2_ (Figure 9B) at 0 K, as calculated from density functional theory (DFT) simulations, show no imaginary modes at the Gamma (Γ) point or at any high‐symmetry point, indicating thermodynamic and dynamic stability. Moreover, the lack of imaginary modes at any high‐symmetry point in the phonon band structures of Nb_3_SiNi_2_ (Figure 9C) and Ni_3_SiNb_2_ (Figure 9D) at 1723 K suggests thermodynamic and dynamic stability up to this rather elevated temperature. Additional computational results on the phase stability of Nb_3_SiNi_2_ & Ni_3_SiNb_2_ and of derivative binary compounds produced without altering the elemental ratios in their stoichiometries are presented in Section S3.1 (Supporting Information). Assessing the stability of candidate binary compounds derived from “parent” ternary IMCs was a first step in the tentative exploration of the possible production of ZIP phase 2D derivatives. A second step explored the thermodynamic stability of surfaces formed via the etching of Nb_3_SiNi_2_ and Ni_3_SiNb_2_ along specific planes (Section S3.3, Supporting Information). Despite the scientific interest of a protracted discussion on the formation and stability of 2D derivatives, this topic is only addressed in the Supporting Information, as this work focuses on the ZIP phase ternary IMCs and not their 2D derivatives.

The electronic density of states (eDOS) in the hexagonal Ni_3_SiNb_2_ ZIP phase (Figure 9E) suggests metallic behavior, as calculated using hybrid DFT (HSE06 functional). Figure 9F depicts the reduced charge density in the hexagonal Ni_3_SiNb_2_ ZIP phase variant, offering a visualization of the interactions between atoms. As there is no charge density between atoms, the bonding network is considered ionic. Additional information on bond analysis is provided in Section S3.2 (Supporting Information).

### 2.3 Expanding the ZIP Phase Concept to Other Ternary Systems

#### 2.3.1 Synthesis of ZIP Phases in the Nb‐Si‐Co, Ta‐Si‐Ni, and V‐Si‐Ni Systems

As this work proposes the existence of a whole new family of nanostructured ternary IMCs, each member of which exhibits “dualistic atomic ordering”, thus forming one *fcc* and one hexagonal variant, it was deemed imperative to synthesize ZIP phases in other ternary systems. Due to the sheer magnitude of this quest, especially in the absence of equilibrium phase diagrams, it has been decided to use spark plasma sintering (SPS) to make selected ternary IMCs previously reported in literature. For example, apart from the *fcc* Nb_3_SiNi_2_ ternary IMC considered as the “ternary H‐phase” of the Nb‐Si‐Ni system,^[^ ^44^, ^46^, ^47^ ^]^ other ternary IMCs with similar “312” stoichiometry and the same crystal structure (*Fd* $\bar{3}$ *m*, SG 227) have been reported in the Mn‐Si‐Ni (Mn_3_SiNi_2_),^[^ ^44^, ^57^, ^58^ ^]^ V‐Si‐Ni (V_3_SiNi_2_),^[^ ^44^ ^]^ Cr‐Si‐Ni (Cr_3_SiNi_2_),^[^ ^44^ ^]^ Ta‐Si‐Ni (Ta_3_SiNi_2_),^[^ ^44^, ^59^ ^]^ Nb‐Si‐Co (Nb_3_SiCo_2_),^[^ ^60^ ^]^ Na‐Au‐In (Na_3_AuIn_2_),^[^ ^12^ ^]^ Na‐Ag‐In (Na_3_AgIn_2_),^[^ ^12^ ^]^ and Mg‐Ga‐Ni (Mg_3_GaNi_2_)^[^ ^61^ ^]^ systems. Few ternary IMCs with the “312” stoichiometry and the hexagonal crystal structure (*P6_3_/mmc*, SG 194) have also been sporadically reported in prior studies. Examples include hexagonal ternary IMCs in the Co‐Si‐Nb (Co_3_SiNb_2_),^[^ ^60^ ^]^ Ni‐Si‐Nb (Ni_3_SiNb_2_),^[^ ^45^, ^46^, ^47^ ^]^ and Cu‐Si‐Mg (Cu_3_SiMg_2_)^[^ ^60^ ^]^ systems.

**Figure 10:**
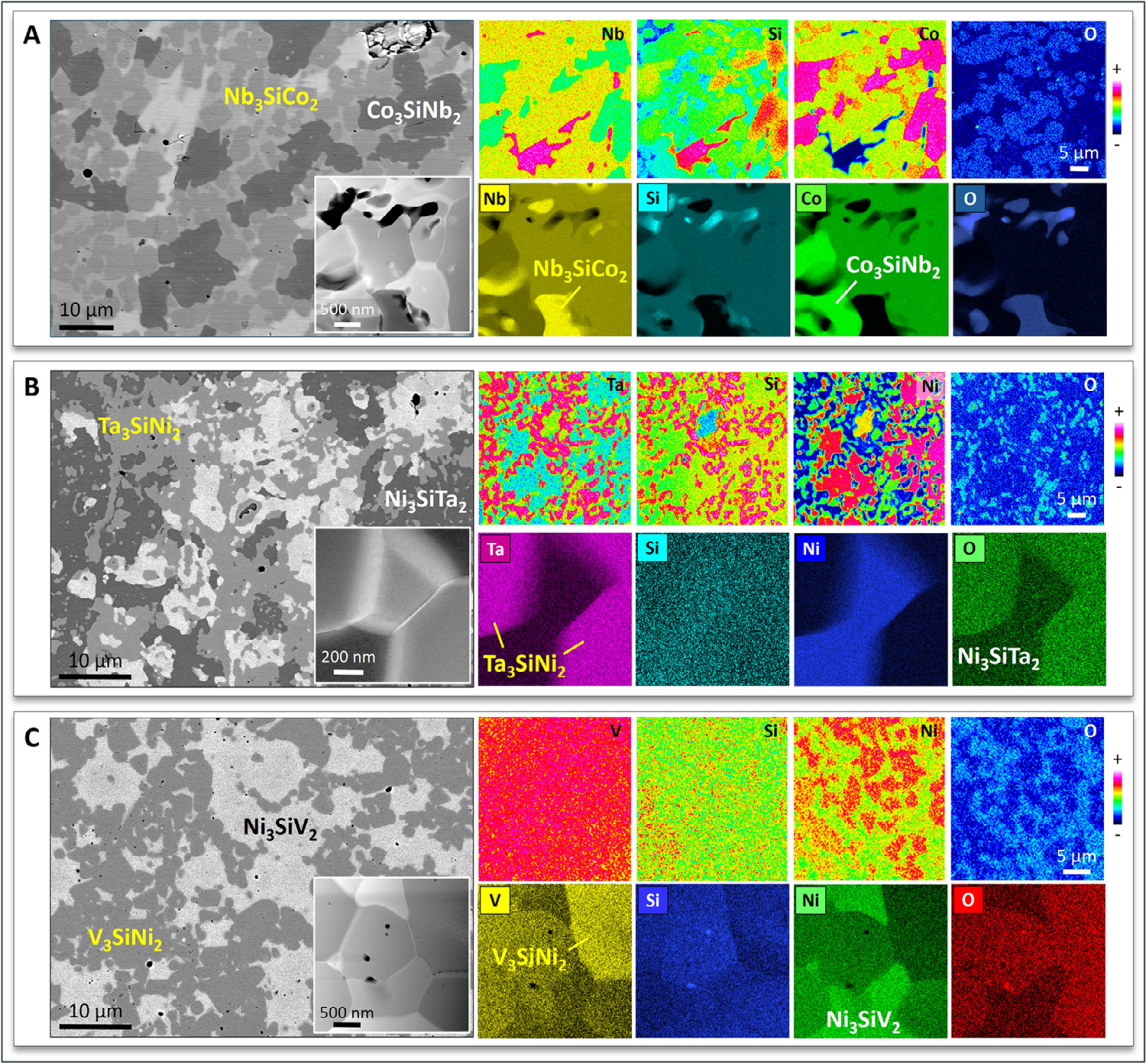
Microstructural characterization of SPS intermetallic alloys in the Nb‐Si‐Co A), Ta‐Si‐Ni B), and V‐Si‐Ni C) systems. Despite the low phase purity of these three alloys, EPMA/WDS (top rows) and HAADF STEM/EDS (bottom rows) data confirm the formation of the Nb_3_SiCo_2_ and Co_3_SiNb_2_ ZIP phases in the Nb‐Si‐Co alloy A); the Ta_3_SiNi_2_ and Ni_3_SiTa_2_ ZIP phases in the Ta‐Si‐Ni alloy B); and the V_3_SiNi_2_ and Ni_3_SiV_2_ ZIP phases in the V‐Si‐Ni alloy C). The HAADF STEM insets in the bottom right‐hand corner of the SEM micrographs of the three alloys correspond to the areas analyzed by HAADF STEM/EDS.

In this work, SPS was employed to synthesize intermetallic alloys in the Nb‐Si‐Co (**Figure** **10**A), Ta‐Si‐Ni (Figure 10B), and V‐Si‐Ni (Figure 10C) systems; the main aim of these trial synthesis efforts was to demonstrate the formation of ZIP phases in these three ternary systems. Characterization of all SPS intermetallic alloy samples by means of EPMA/WDS showed that despite the low phase purity (expected, in view of the limited synthesis trials), the two ZIP phase variants formed in each ternary system, usually appearing intimately linked with each other (also expected, as they comprise the same elements in different ratios). HAADF STEM/EDS analysis of FIB foils from all intermetallic alloy samples, combined with Rietveld‐refined XRD analysis of the SPS samples (Figures S16,S17, and S18, Supporting Information), confirmed the formation of the two ZIP phase variants in these ternary systems; moreover, the *fcc* ZIP phase variants showed invariably much higher O contents than their hexagonal counterparts, in agreement with the ZIP phases in the Nb‐Si‐Ni system (see Figures 2B and 5A,B; Figure S9, Supporting Information). Producing phase‐pure materials in the Nb‐Si‐Co, Ta‐Si‐Ni, V‐Si‐Ni or other ternary systems falls outside the scope of this work; however, high phase purity is vital for the accurate determination of material properties. The systematic calculation of equilibrium phase diagrams in ZIP phase‐forming ternary systems will surely assist and guide the synthesis of phase‐pure ZIP phase materials.

#### 2.3.2 Synthesis of ZIP Phases in the Nb‐Si‐Fe System

It is worthwhile noting that in all hitherto reported *fcc* ternary IMCs now considered as candidate ZIP phases, the electronegativity, χ, of the Z‐element is invariably smaller than the electronegativities of both I‐ and P‐elements (see Table S12, Supporting Information). Moreover, the atomic radius of the Z‐element is larger than the atomic radii of both I‐ and P‐elements, with the exception of Cr_3_SiNi_2_, where the atomic radius (125 pm) of the Z‐element, Cr, is equal to that of the P‐element, Ni (Table S12, Supporting Information). One interesting way to look at the ZIP phases is by considering that the hexagonal variant is the “compositionally inverted” *fcc* variant, in the sense that the two ZIP phase variants are produced by simply interchanging the Z‐element with the P‐element whilst continuing to respect the “312” stoichiometry that is also shared by the 312 MAX phases (i.e., M_n+1_AX_n_ where n = 2). Based on the above considerations, the electronegativity and atomic radii trends that govern the formation of the *fcc* ZIP phase variants will also be sort of “inverted” in the hexagonal ZIP phase counterparts. This means that the electronegativity of the P‐element will be smaller than the electronegativities of both Z‐ and I‐elements, whereas the atomic radius of the P‐element will be larger than the atomic radii of the Z‐ and I‐elements in the hexagonal ZIP phases. For example, for the Nb_3_SiNi_2_ and Ni_3_SiNb_2_ ZIP phases, *χ*(Nb) = 1.60 < *χ*(Si) = 1.90, *χ*(Ni) = 1.91 and *r*(Nb) = 143 pm > *r*(Si) = 118 pm, *r*(Ni) = 125 pm (Table S12, Supporting Information), irrespective of whether Nb occupies the Z‐site in the *fcc* variant or the P‐site in the hexagonal one. Comparing the hexagonal Ni_3_SiNb_2_ ZIP phase variant (Figure 4B) with the hexagonal Ti_3_SiC_2_ MAX phase (Figure 4C) – they both crystallize in SG *P6_3_/mmc*– shows that these nanolaminated solids follow opposite electronegativity and atomic radii trends, i.e., *χ*(Nb) < *χ*(Si), *χ*(Ni) and *r*(Nb) > *r*(Si), *r*(Ni) for Ni_3_SiNb_2_ versus *χ*(C) = 2.55 > *χ*(Si) = 1.90, *χ*(Ti) = 1.54 and *r*(C) = 77 pm < *r*(Si) = 118 pm, *r*(Ti) = 145 pm for Ti_3_SiC_2_ (Table S12, Supporting Information).

Based on the herein identified trends in electronegativity and atomic radii that govern ZIP phase formation, it was decided to attempt the synthesis of ZIP phases in the Nb‐Si‐Fe ternary system, as no ZIP phases have been previously synthesized and/or reported in that system. Succeeding in this effort would reinforce our claim for the existence of a whole new family of ternary IMCs with dualistic atomic ordering, named the ZIP phases.

**Figure 11:**
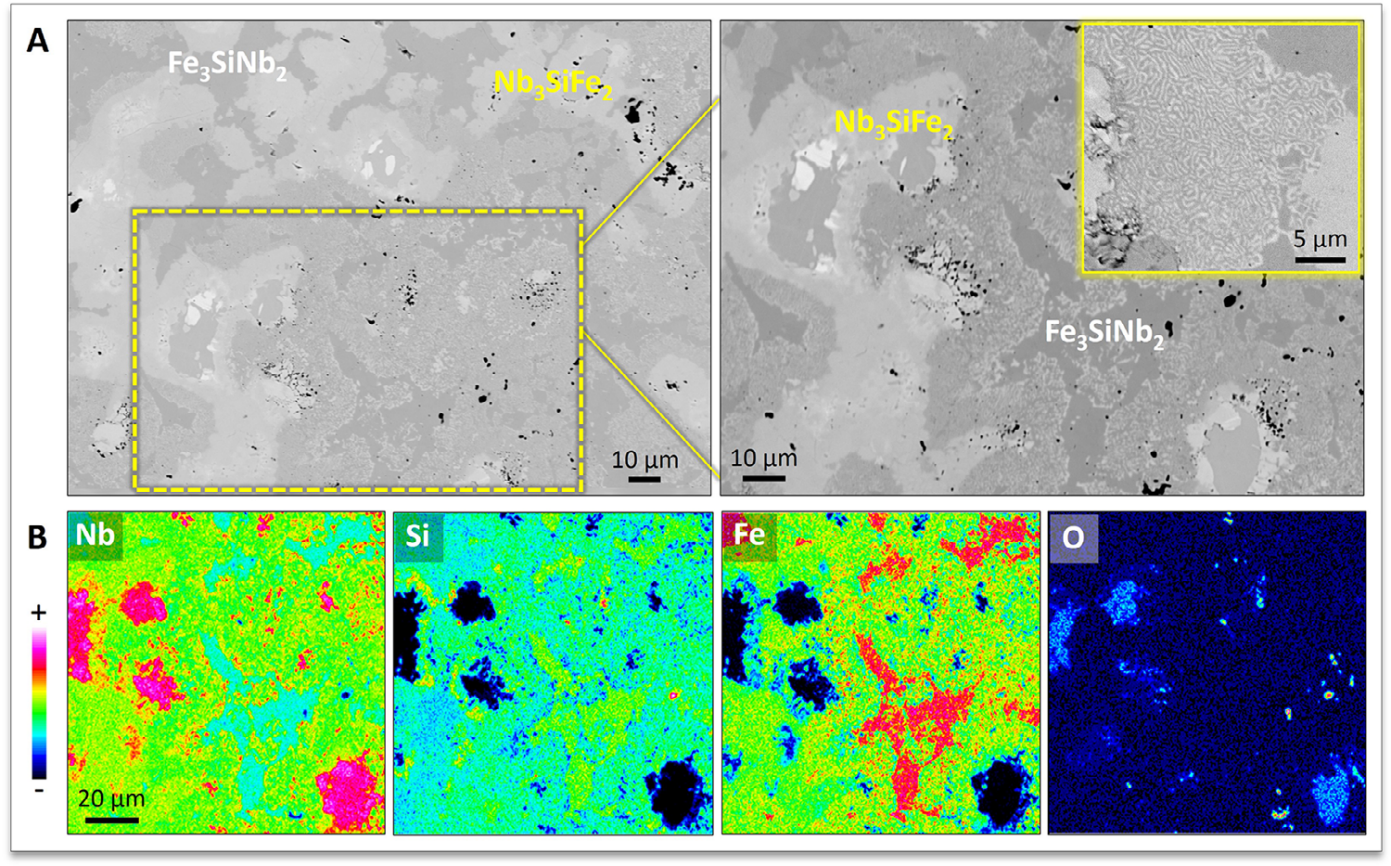
Characterization of an RHP Nb‐Si‐Fe intermetallic alloy sample by means of A) SEM and B) EPMA/WDS. WDS identified the Nb_3_SiFe_2_ and Fe_3_SiNb_2_ ZIP phases in the phase assembly. The O solubility in Nb_3_SiFe_2_ appears higher than in Fe_3_SiNb_2_. The inset SEM micrograph shows the formation of a eutectic structure in this system.

Characterization of the RHP Nb‐Si‐Fe intermetallic alloy samples sintered at 1723 K by means of EPMA/WDS (**Figure** **11**) and Rietveld‐refined XRD (Figure S19, Supporting Information) confirmed the formation of the two ZIP phase variants in the Nb‐Si‐Fe system, as predicted based on the herein identified electronegativity and atomic radii trends vis‐à‐vis ZIP phase formation. XRD analysis suggested that the formation of the hexagonal Fe_3_SiNb_2_ ZIP phase variant might have been enabled by the formation of a Fe‐rich solid solution of the pseudo‐binary Nb(Fe,Si)_2_ IMC. The catalytic role of “precursor” solid solution IMCs in the formation of ZIP phases – also observed by Goossens et al.^[^ ^52^ ^]^ during MAX phase formation − is likely to become a topic of future research. Former studies in support of this prediction include the work of dos Santos et al.,^[^ ^47^ ^]^ who mentioned appreciable Si solid solubility in the Nb_7_Ni_6_ binary IMC; the work of Gladyshevskii et al.,^[^ ^44^ ^]^ who reported the formation of Si solid solutions based on the Nb_3_Fe_2_ binary IMC; and the work of Schön and Tenório,^[^ ^62^ ^]^ who observed that the dissolution of small amounts of phosphorus (P) and sulfur (S) in Fe‐Nb alloys stabilized a pseudo‐binary Nb_3_Fe_2_ IMC with the *fcc* diamond cubic structure (SG *Fd* $\bar{3}$ *m*). Based on their electronegativity and atomic radius values, P (*χ* = 2.19, *r* = 110 pm) and S (*χ* = 2.58, *r* = 103 pm) are “allowed” to occupy the I‐site in ZIP phases containing Nb and Fe (possibly even forming solid solutions on this site). Demonstrating ZIP phase synthesis in the Nb‐(P,S)‐Fe system – a candidate topic of future research – would support the idea that “precursor” binary IMCs (e.g., Nb_3_Fe_2_) promote ZIP phase formation.

### 2.4 Comparing the ZIP Phases with the MAX Phases

In the search for nanostructured materials with exceptional properties, both the herein proposed ZIP phases and the MAX phases share the same historical roots, dating back to the advent of space exploration in the 1960s.^[^ ^7^, ^8^, ^44^, ^46^, ^47^, ^63^ ^]^ Despite the fact that they have been both discovered in the same period of time, there is very little, if any, knowledge of the properties and possible applications of the ternary IMCs named “the ZIP phases” and their potential 2D derivatives, as opposed to the already extensively studied ternary early transition metal carbides and nitrides named “the MAX phases”, their higher order solid solutions, and their 2D derivatives known as MXenes. Therefore, this section will attempt to discuss the rather mature MAX phases in association with the emerging ZIP phases, identifying potential new opportunities arising from the advent of the ZIP phases based on the experience gained through the MAX‐phase research over the past 30 years.

**Figure 12:**
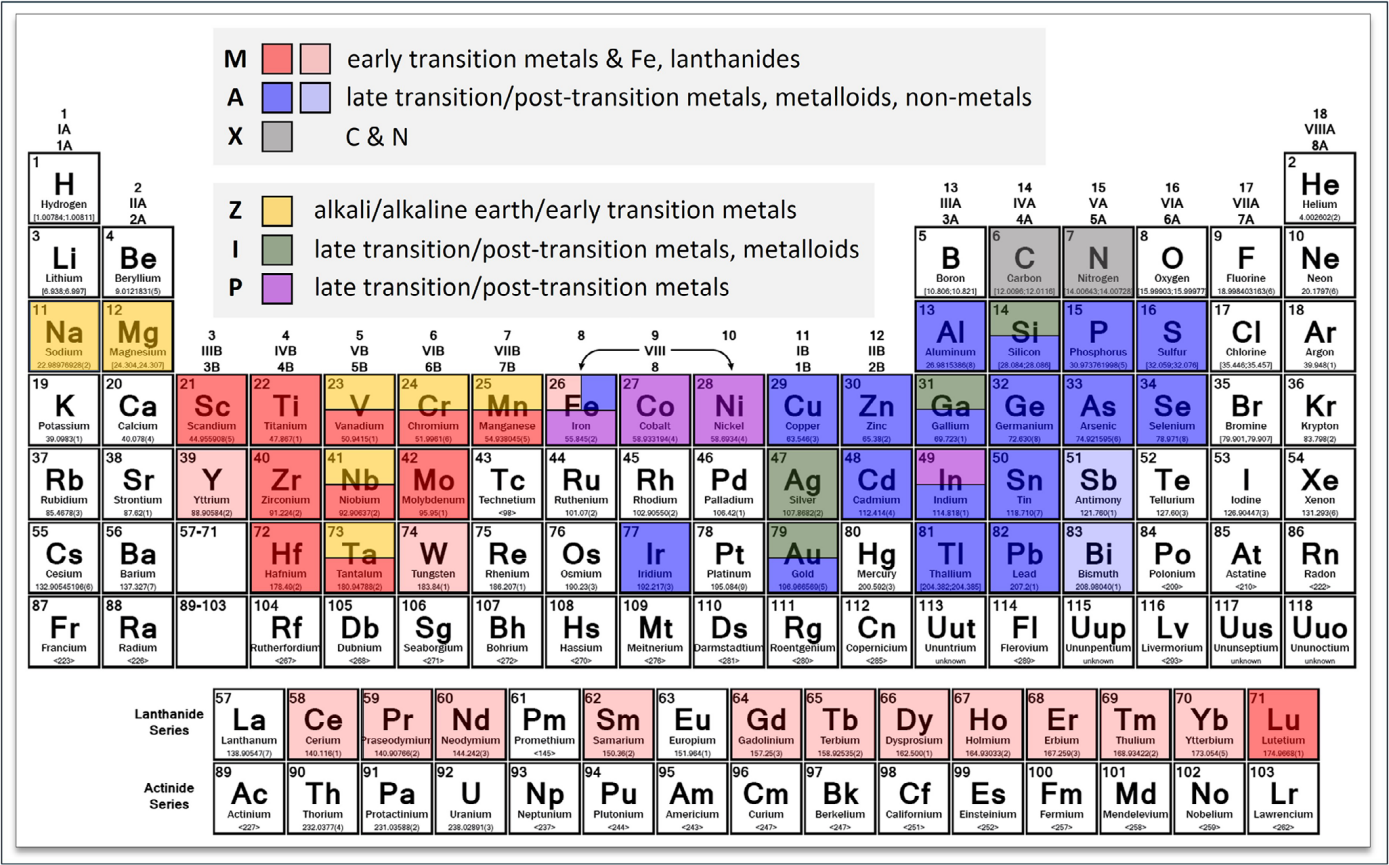
Distribution of the M, A, and X elements (found in reported MAX phases) and the Z, I, and P elements (as encountered in synthesized *fcc* ZIP phases) in the periodic table of elements. Together, the two classes of solids span almost the entire periodic table, comprising jointly elements from groups 1‐16. The M‐elements of the MAX phases appear in two shades of red: the darker indicates full M‐site occupancy, the lighter partial occupancy (solid solutions). The A‐elements also appear in two shades of blue, depending on the degree of A‐site occupancy.

The herein introduced family of ternary IMCs, named the ZIP phases, and the family of ternary early transition metal carbides/nitrides, known as the MAX phases, constitute a set of nanostructured solids with the same overarching stoichiometry that spans almost the entire periodic table of elements, comprising jointly constituent elements from groups 1–16. **Figure** **12** is a color‐coded periodic table of elements showing that the MAX phases are made of M‐elements from groups 3–7 (early transition metals); A‐elements that belong mainly to groups 13–15 (post‐transition metals, metalloids & non‐metals) with limited expansion to groups 8–12 (late transition metals) and group 16 (non‐metals); and X‐elements that are either C or N (non‐metals in groups 14 and 15, respectively). On the other hand, the so far synthesized *fcc* ZIP phase variants utilize Z‐elements from groups 1–2 (alkali metals & alkaline earth metals) and groups 5–7 (early transition metals); I‐elements from group 11 (late transition metals) and groups 13–14 (post‐transition metals & metalloids); and P‐elements from groups 8–10 (late transition metals) and group 13 (post‐transition metals). As discussed earlier, both ZIP and MAX phases share the “312” MAX phase stoichiometry, i.e., M_n+1_AX_n_ where n = 2. A review by Goossens et al.^[^ ^10^ ^]^ pointed out that the MAX phases typically belong either to one of the three well‐known orders 211, 312 and 413, or exhibit one of the following exceptional stoichiometries: i) M_n+1_AX_n_‐like structures with *n* > 3, such as Ta_6_AlC_5_ and Ti_7_SnC_6_, or ii) “hybrid” orders M_5_A_2_C_3_ (e.g., Hf_5_AlC_3_,^[^ ^64^ ^]^ or Ti_5_A_2_C_3_ with A = Si, Ge, and Al) and M_7_A_2_C_5_ (e.g., Ti_7_Si_2_C_5_). The “hybrid” orders M_5_A_2_C_3_ and M_7_A_2_C_5_ have been regarded as combinations of a 312 atomic stacking with a 211 and a 413 order, respectively, as they do not obey the M_n+1_AX_n_ general MAX phase stoichiometry. However, considering carefully the two “312” ZIP phase variants and all MAX phase orders, including the “hybrid” ones and the only so far identified member of the 514 order, i.e., the Mo_4_VAlC_4_ compound,^[^ ^65^ ^]^ suggests that it is possible to describe both classes of nanostructured solids by the overarching stoichiometric rule P_x+y_A_x_N_y_ (PAN), where x = 1 or 2, and y = 1, 2, 3, 4, 5, or 6, according to all “PAN phases” so far experimentally synthesized. The herein proposed overarching PAN stoichiometric rule reconciles the “hybrid” orders M_5_A_2_C_3_ and M_7_A_2_C_5_ with the common MAX phase orders 211, 312 & 413, answering affirmatively to the question whether it is conceptually correct to include the “hybrid” orders in the MAX phase family.

Another important aspect is that the two families of nanostructured solids have specific crystal structure similarities, i.e., both the MAX phases and one of the two ZIP variants are hexagonal nanolaminated solids with identical crystal structure (*P6_3_/mmc*, SG 194). In this sense, the hexagonal ZIP phase variants expand the MAX phases concept of crystal structure nanolamination beyond the early transition metals and into the late transition metals (e.g., Fe, Co, Ni) and post‐transition metals (e.g., In). This heralds the potential fabrication of higher‐order solid solutions and 2D derivatives of the hexagonal ZIP phases, similar to the complex MAX phase solid solutions and their 2D derivatives (MXenes). As MAX phase solid solution synthesis has proven a very potent tool, allowing the fabrication of ceramics tailored to the property requirements of the targeted application(s), it is reasonable to expect similar benefits from the further development of the ZIP phases. This expectation is based on the fact that the ZIP phases comprise elements that do not favor MAX phase formation (e.g., alkali & alkaline earth metals, late transition metals), thus paving the way for the synthesis of novel nanostructured solids with properties atypical for the MAX phases (e.g., magnetic properties; superconductivity; tailorable ductility; etc.). This hypothesis finds first support in the work of Kolenda et al.,^[^ ^58^ ^]^ who reported magnetic properties in the Mn_3_SiNi_2_ member of the ZIP phase family. Magnetic properties were also determined for the Nb_3_SiNi_2_ and Ni_3_SiNb_2_ ZIP phases produced in this work (see Figures 7 and 8), whereas superconductivity was discovered in the Nb_3_SiNi_2_ ZIP phase (Figure 7F). By contrast, magnetic properties and superconductivity have been particularly difficult to impart and/or measure in MAX phase compounds. In the plethora of studies on the properties of the MAX phases, only a handful of studies have measured magnetic properties in experimentally synthesized MAX phases, i.e., the (Cr_0.75_, Mn_0.25_)_2_GeC MAX phase solid solution,^[^ ^66^ ^]^ rare‐earth‐containing *i*‐MAX phase solid solutions (i.e., MAX phase compounds with in‐plane ordered M‐elements) of the (Mo_2/3_, RE_1/3_)_2_AlC stoichiometry, where RE = Ce, Pr, Nd, Sm, Gd, Tb, Dy, Ho, Er, Tm, and Lu,^[^ ^24^, ^67^ ^]^ and (Ti_1−x_,Fe_x_)_3_AlC_2_ MAX phase solid solutions, where x = 0, 0.025, 0.05, and 0.075.^[^ ^68^ ^]^ Superconductivity has only been reported for the Lu_2_SnC ternary MAX phase compound.^[^ ^69^ ^]^ It is reasonable to assume that the new range of properties achieved by the ZIP phases (e.g., magnetic properties, superconductivity) might be transferable to their 2D derivatives (assuming these can be synthesized), giving rise to the next generation of nano‐tailored advanced materials for use in flexible and wearable electronics, batteries, quantum computing, high‐sensitivity magnetic sensors, functionalized templates for biomedical applications, etc.

A unique feature of the ZIP phases is that the I‐ and P‐elements can belong to the same group of the periodic table. For example, the P‐element (In) in Na_3_AuIn_2_ and Na_3_AgIn_2_, as well as the I‐element (Ga) in Mg_3_GaNi_2_, belong to group 13. Such freedom in choosing the elements making up the ZIP phases does not exist in the MAX phases, where only two X‐elements are allowed (i.e., C or N), and these are not known to be replaced by any of the candidate M‐ or A‐elements. The only known element with flexibility to occupy either the M‐ or A‐site in specific MAX phases is iron (Fe), which belongs to group 8 of late transition metals. More specifically, Fe has been either reported to form the (Ti_1−x_,Fe_x_)_3_AlC_2_ solid solutions on the M‐site,^[^ ^68^ ^]^ or to occupy fully the A‐site of the ternary MAX phases Ta_2_FeC, Ti_2_FeN, and Nb_2_FeC.^[^ ^70^ ^]^ The allowable combinations of elemental constituents in the ZIP phases could tentatively be associated with relative differences in the electronegativity and atomic radius values of the Z/I/P‐elements; however, before strict rules governing ZIP phase formation are proposed, further systematic research is needed to better map, in terms of attainable chemical compositions, the ZIP phases' “landscape”.

A cornerstone in the prospective exploitation of the ZIP phases is the synthesis of phase‐pure materials, as high phase purity is a prerequisite for the determination of material properties, such as elastoplastic/fracture behavior, corrosion resistance, electromagnetic properties, radiation tolerance, etc., thereby exploring the possible applications of the ZIP phases and/or their 2D derivatives. Challenges in producing phase‐pure (bulk) materials have also been reported for the Zr/Hf‐based MAX phases.^[^ ^20^, ^21^, ^38^, ^40^, ^71^ ^]^ Overcoming these challenges involved either the synthesis of complex double solid solutions based on steric stability criteria^[^ ^72^, ^73^ ^]^ or the use of early transition metal hydride starting powders rather than elemental ones.^[^ ^52^ ^]^ Several processing methods, other than RHP, can be explored to synthesize phase‐pure ZIP phases in different forms (bulk, coating) to make progress with property determination and exploration of the application space for both ternary IMCs and their 2D derivatives. For example, physical vapor deposition (PVD) methods, such as magnetron sputtering, are most likely suitable for the deposition of ZIP phases (i.e., IMCs with very precise stoichiometries and appreciable structural complexity) on different substrates, suppressing the formation of competing phases, and producing strongly textured, phase‐pure coatings that might be desirable for various high‐tech applications.

## 3 Conclusion and Outlook

This work introduced a new family of nanostructured ternary intermetallic compounds (IMCs), aptly named the ZIP phases due to their distinctive “zigzag” atomic arrangements. Each member of the ZIP phase family manifests itself in two variants, one with the *fcc* diamond cubic structure (*Fd* $\bar{3}$ *m*, SG 227) and one with the hexagonal structure (*P6_3_/mmc*, SG 194); the latter is identical to the structure of the hexagonal MAX phases. All ZIP phases obey the 312 MAX phase stoichiometric rule, i.e., M_n+1_AX_n_ with n = 2, or, alternatively, the herein proposed overarching ZIP/MAX stoichiometric rule P_x+y_A_x_N_y_ (PAN) with x = 1 and y = 2. The hitherto synthesized ZIP phases (sixteen ternary IMCs, in total) comprise Z‐elements from groups 1‐2 (alkali metals & alkaline earth metals) and groups 5–7 (early transition metals); I‐elements from group 11 (late transition metals) and groups 13‐14 (post‐transition metals & metalloids); and P‐elements from groups 8‐10 (late transition metals) and group 13 (post‐transition metals). The MAX phases (ternary early transition metal carbides & nitrides) and the ZIP phases (ternary IMCs) constitute a set of nanostructured solids that spans almost the entire periodic table of elements, comprising jointly elements from groups 1–16. This implies that the hexagonal ZIP phase variants expand the MAX phases concept of crystal structure nanolamination beyond the early transition metals and well into the late transition and post‐transition metals, thus enabling the synthesis of new nanostructured solids and potential 2D derivatives with properties (e.g., magnetism) that continue to elude the MAX phases and their 2D derivatives known as MXenes.

In this work, ZIP phase‐based materials in the Nb‐Si‐Ni, Nb‐Si‐Co, Ta‐Si‐Ni, V‐Si‐Ni, and Nb‐Si‐Fe ternary systems were synthesized by means of reactive hot pressing (RHP) and spark plasma sintering (SPS), proving the formation of the two ZIP variants in all five ternary systems. Quite importantly, RHP was successfully and reproducibly able to synthesize quasi phase‐pure Nb_3_SiNi_2_ and Ni_3_SiNb_2_ materials, thus demonstrating (scalable) synthesis feasibility and paving the way for the further development and exploitation of the ZIP phases. The experimentally synthesized Nb_3_SiNi_2_ and Ni_3_SiNb_2_ ZIP phase variants were characterized in terms of crystal structure; spatially resolved composition and reconstructed atomic positions; thermal, electrical, and magnetic properties; and selected mechanical and physical properties. Moreover, density functional theory (DFT) and the temperature‐dependent effective potential (TDEP) method were employed to assess the stability of the ZIP ternary IMCs and candidate derivative binary compounds as a function temperature, also exploring the possible exfoliation of the ZIP phase variants along specific planes in order to produce 2D derivatives; the latter analysis showed that the hexagonal ZIP phase variant is more prone to exfoliation than its *fcc* counterpart. The ZIP phases are characterized by metallic overall behavior and ionic bonding.

The advent of the ZIP phases is expected to create a new ecosystem of nanostructured ternary IMCs with dualistic atomic ordering and potential 2D derivatives with properties (e.g., magnetism, tailorable ductility, superconductivity, etc.) that cannot be found in other nanostructured or nanolaminated solids, such as the MAX phases and their 2D derivatives (MXenes). This paves the way for the exploration of a host of deep tech applications in diverse technology sectors, including flexible/wearable electronics; batteries; quantum computing; highly sensitive magnetic sensors; biomedical applications aiming at smart drug delivery; etc. The structural complexity of the ZIP phases (e.g., the unit cell of Nb_3_SiNi_2_ has 96 atoms) renders them, after a fashion, comparable to high‐entropy alloys (HEAs), the chemical complexity of which imparts them with remarkable radiation tolerance. At this stage, it is impossible to make accurate predictions as to the full range of applications of the ZIP phases. The first step toward the further exploitation of this remarkable new family of materials is the synthesis and full characterization of phase‐pure materials in various ZIP phase‐forming ternary systems. A second step of great importance would undoubtedly be the experimental synthesis of proof‐of‐concept 2D derivatives of the ZIP phases.

## 4 Experimental Section

### Synthesis of Nb_3_SiNi_2_ via Arc Melting

Conventional vacuum arc melting of high‐purity (>99.99%) Nb, Ni, and Si elemental raw powders was initially employed to synthesize the Nb‐Si‐Ni intermetallic alloy with the targeted H‐phase Nb_3_SiNi_2_ stoichiometry, i.e., 50.0Nb‐16.7Si‐33.3Ni in at.%. Arc melting yielded a 200‐g disc of low phase purity, even though the ratio of elemental powders in the powder feedstock had been adjusted to match the Nb_3_SiNi_2_ stoichiometry. Additional information on the arc melting synthesis of alloys in the Nb‐Si‐Ni ternary system may be found elsewhere.^[^ ^45^, ^46^, ^47^ ^]^

The thermal and microstructural stability of the as‐cast Nb‐Si‐Ni intermetallic alloy was assessed via an isothermal annealing treatment at 1421 K for 2 weeks (336 h) in a vacuum furnace (ThermoLyne furnace, model 46 100). The as‐cast alloy samples were pre‐encapsulated in a quartz (SiO_2_) tube, which was pressurized with argon (Ar) gas to 1 atm; both heating and cooling rates were fixed at ≈14.2 K/min.

### Synthesis of Nb_3_SiNi_2_ via Reactive Hot Pressing (RHP)

The failure to produce a phase‐pure Nb‐Si‐Ni intermetallic alloy by arc melting redirected the synthesis efforts in this work toward a powder metallurgical processing route. Discs of the Nb‐Si‐Ni intermetallic alloy (∼26 g each) with high phase purity were successfully produced via reactive hot pressing (RHP), using hydride and elemental starting powders, i.e., NbH_0.89_ (<40 µm, CBMM, Brazil), Ni (Vale, T123, 3‐7 µm, UK), and Si (A10, 2.1 µm, HC Starck, Germany). To optimize the mixing of the starting powders and improve powder particle contact, the as‐received NbH_0.89_ powder was first refined by low‐energy ball milling in isopropanol for 24 h (Turbula T2), using WC‐6 wt.% Co (WC‐6Co) milling balls with diameters of 5 and 10 mm. The refined NbH_0.89_ powder was dried in a rotating evaporator (Heidolph 4010) and then mixed with the Ni and Si elemental powders in isopropanol for another 24 h, using WC‐6Co milling balls. The ratio of NbH_0.89_, Ni, and Si in the powder feedstock was adjusted to the targeted 3Nb:1Si:2Ni. After mixing, the feedstock powder suspension was dried in a rotating evaporator.

The powder feedstock was cold‐pressed into “green” pellets (∅ 30 mm) under a load of 30 MPa in a graphite die. Exploratory RHP runs were conducted in a hot press (W100/150‐2200‐50 LAX, FCT Systeme, Frankenblick, Germany). The powder compacts were heated in vacuum (0.4 mbar) at 10 K/min to different sintering temperatures (i.e., 1523, 1623, and 1723 K), where densification was achieved under a uniaxial pressure of 30 MPa for 60 min. The sintered discs (∅ 30 mm, 4.5 mm in thickness) were allowed to cool naturally by switching off the power supply of the hot press. RHP was also used to produce a larger disc of Nb_3_SiNi_2_ (∅ 56 mm, 4.5 mm in thickness) to assess mechanical properties and hardness; this disc was sintered at 1723 K for 60 min under a uniaxial pressure of 15 MPa.

### Synthesis of ZIP Phases in Other Ternary Systems—Synthesis of Previously Reported Ternary IMCs of the ZIP Phase Type

Spark plasma sintering (SPS) and RHP were employed to produce discs with ternary IMCs in the Nb‐Si‐Co, Ta‐Si‐Ni, V‐Si‐Ni, and Fe‐Si‐Nb systems, thus proving that the ZIP phases are indeed a family of ternary IMCs with two variants, i.e., an *fcc* (SG *Fd* $\bar{3}$ *m*) variant and a hexagonal (SG *P6_3_/mmc*) variant. The starting powders used to synthesize the Nb‐Si‐Co intermetallic alloy with the Nb_3_SiCo_2_ stoichiometry were NbH_0.89_ (<40 µm, CBMM, Brazil), Co (Co‐HMP, FSSS 0.55 µm, Umicore, Belgium), and Si (A10, 2.1 µm, HC Starck, Germany). The NbH_0.89_ powder was refined by low‐energy ball milling in isopropanol for 24 h (Turbula T2), using WC‐6Co milling balls (diameters of 5 mm and 10 mm). The NbH_0.89_, Co, and Si powders were mixed in isopropanol for another 24 h, using WC‐6Co milling balls. After mixing, the feedstock powders were dried in a rotating evaporator. The dried powders were densified by means of SPS (HP D 25, FCT Systeme, Frankenblick, Germany), in vacuum, at 1723 K, under a uniaxial pressure of 30 MPa for 2 min; the powder compact was heated to 1723 K at 100 K/min and cooled by switching off the power supply. The sintered disc was 30 mm in diameter and 4.5 mm in thickness.

The feedstock powders used to synthesize the Ta‐Si‐Ni intermetallic alloy of the Ta_3_SiNi_2_ stoichiometry were Ta (FTa‐1, 2 µm, Ningxia Orient Tantalum Industry Co., Ltd., China), Ni (Vale, T123, 3‐7 µm, UK), and Si (A10, 2.1 µm, HC Starck, Germany). The Ta, Ni, and Si powders were dry‐mixed for 3 min at 1800 rpm, using a high‐speed mixer (DAC 150.1 FWZ‐K, Flacktek, Germany). The powders were densified by SPS (HP D 25, FCT Systeme, Frankenblick, Germany), in vacuum, at 1623 K, under a uniaxial pressure of 30 MPa for 10 min; the powder compact was heated to 1623 K at 100 K/min and cooled by switching off the power supply. The sintered disc was 30 mm in diameter and 4.5 mm‐thick.

The starting powders used to synthesize the V‐Si‐Ni intermetallic alloy with the V_3_SiNi_2_ stoichiometry were V_2_H (<20 µm, lab grade), Ni (Vale, T123, 3‐7 µm, UK), and Si (A10, 2.1 µm, HC Starck, Germany). The V_2_H powder was synthesized via the hydrogenation of V powder (<44 µm, Alfa Aesar, Germany) in a horizontal tube furnace (Carbolite type CTF), under continuous flow of hydrogen (H_2_) gas (Air Liquide N40). Powder hydrogenation was achieved by slowly heating (5 K/min) the V powder to 1093 K, holding for 5 h, and slowly cooling (5 K/min) to room temperature (RT). Switching the gas flow to technical argon (Ar; 3‐5 ppm O_2_, Nippon Gases Ltd.) after cooling allowed the removal of any remaining H_2_ without risking the spontaneous oxidation of V_2_H due to its sudden exposure to oxygen (O_2_). Vibratory ring milling (Retsch RS200) for 30 s at 1100 rpm of the brittle V_2_H powder resulted in powder refinement; the powder was sieved and only its finer fraction (<20 µm) was used for further processing. The feedstock powders were mixed in isopropanol for 24 h using WC‐6Co milling balls and were subsequently dried in a rotating evaporator. The dried powders were densified by SPS (HP D 25, FCT Systeme, Frankenblick, Germany), in vacuum, at 1523 K, under a uniaxial pressure of 30 MPa for 10 min; the powder compact was heated to 1523 K at 100 K/min and cooled by switching off the power supply. The sintered disc diameter was 30 mm, and its thickness was 4.5 mm.

### Synthesis of ZIP Phases in Other Ternary Systems—Synthesis of Predicted ZIP Phases in the Nb‐Si‐Fe System

The starting powders used to synthesize the Nb‐Si‐Fe intermetallic alloy with the Nb_3_SiFe_2_ stoichiometry were NbH_0.89_ (<40 µm, CBMM, Brazil), Fe (Carbonyl Iron, 3.6 µm, BASF, Germany), and Si (A10, 2.1 µm, HC Starck, Germany). The NbH_0.89_, Fe, and Si powders were dry‐mixed for 24 h without milling balls (Turbula T2). The feedstock powders were densified in a hot press (W100/150‐2200‐50 LAX, FCT Systeme, Frankenblick, Germany), in vacuum, at 1723 K, under a uniaxial pressure of 30 MPa for 120 min; the powder compact was heated to 1723 K at 10 K/min and cooled by switching off the power supply. The sintered disc had a diameter of 30 mm and a thickness of 4.5 mm.

### Materials Characterization—X‐Ray Diffraction (XRD)

X‐ray diffraction (XRD) was used to identify the phases present in all intermetallic alloy samples at RT. The as‐cast and annealed Nb‐Si‐Ni alloy samples produced by arc melting were analyzed on a Bruker AXS D8 Advance X‐ray diffractometer, using Cu‐Kα radiation (40 kV, 40 mA) in a Bragg‐Brentano geometry; the XRD spectra were recorded at steps of 0.02° in the 20‐90° 2θ range. All RHP and SPS Nb‐Si‐Ni, Nb‐Si‐Co, Ta‐Si‐Ni, V‐Si‐Ni, and Nb‐Si‐Fe alloy samples were first analyzed in bulk form on a Rigaku SmartLab SE XRD system with 1D/teX Ultra 250 HE detector, using Cu‐Kα radiation (40 kV, 40 mA) in a Bragg‐Brentano geometry; the XRD spectra were recorded at steps of 0.05° in the 5‐90° 2θ range, at a scan speed of 0.1° per min. One of the quasi phase‐pure RHP Nb‐Si‐Ni alloy discs (∅ 30 mm) sintered at 1723 K was pulverized for XRD analysis, followed by full Rietveld refinement of the XRD pattern. The powder was obtained by ring milling (Retsch RS200; 30 s, 1100 rpm; stainless steel container), whilst the Rietveld refinement (weighted‐profile R‐factor, R_wp_ < 2.84%) was performed using the Profex version 5.4.1 software package. The Rietveld refinement results, including the sample constituent phases and their lattice parameters (also deduced for the “impurity phases” NbO_2_, SiO_2,_ and Si), the atomic site positions, occupancies & displacements, and the temperature factors, are summarized in Table S2 (Supporting Information). Due to the important insights gained by Rietveld‐refined XRD analysis of the quasi phase‐pure Nb‐Si‐Ni alloy, it was decided to do Rietveld refinement on powder XRD samples produced in a similar manner from the lower phase purity SPS Nb‐Si‐Co, Ta‐Si‐Ni, V‐Si‐Ni, and RHP Nb‐Si‐Fe alloy samples. Rietveld refinement was again performed using the Profex version 5.4.1 software package, and the results (constituent phases and lattice parameters, atomic site positions, and occupancies) are summarized in Table S8 (Supporting Information) for the Nb‐Si‐Co alloy, Table S9 (Supporting Information) for the Ta‐Si‐Ni alloy, Table S10 (Supporting Information) for the V‐Si‐Ni alloy, and Table S11 (Supporting Information) for the Nb‐Si‐Fe alloy.

### Materials Characterization—Scanning Electron Microscopy (SEM) & Electron Probe Microanalysis (EPMA)

Metallographic cross‐sections were prepared from all intermetallic alloy samples that were produced via arc melting, RHP, and SPS; all cross‐sections were polished to a mirror surface finish with a colloidal silica (SiO_2_) suspension (known as OPS) in the final step. The microstructure of as‐cast and annealed Nb‐Si‐Ni alloy samples made by arc melting was studied by means of scanning electron microscopy (SEM; Thermo Fisher Apreo SEM) equipped with energy‐dispersive X‐ray spectroscopy (EDS; EDAX Octane Elite Super detector). Elemental mapping of all RHP and SPS alloy samples was performed by means of electron probe microanalysis (EPMA; JXA‐8530F, JEOL Ltd, Japan) equipped with wavelength‐dispersive X‐ray spectroscopy (WDS; 1‐5 eV, 8 eV at Fe‐K_α_, full scanner type spectrometer). EDS elemental analysis (1 SDD/UTW, 129 eV at Mn‐K_α_) of the identified phases was also performed on the same device at fixed conditions (15 kV accelerating voltage, 100 nA beam current).

### Materials Characterization—Focused Ion Beam (FIB)

Focused ion beam (FIB; FEI Helios 600 dual‐beam FIB/SEM) was used to lift out thin foils (thickness < 100 nm) from as‐cast and annealed Nb‐Si‐Ni alloy samples for inspection by means of transmission electron microscopy (TEM), utilizing the classic lift‐out method.^[^ ^74^ ^]^ Combined platinum/carbon (Pt/C) protective capping layers were deposited on the sites of interest to protect the thin foils from ion beam damage during lift‐out. The thin foils were ion‐milled to electron transparency at 30 keV, with the final thinning/ion beam damage removal step at 2 keV.

Additional FIB foils were prepared from the Nb‐Si‐Co, Ta‐Si‐Ni, and V‐Si‐Ni alloy samples, using a FEI Helios Nanolab 660 dual‐beam FIB/SEM. The regions of interest were covered first with a thin (∼100 nm) Pt layer deposited by the electron beam, followed by a ∼2 µm Pt protective capping layer deposited using the ion beam. Final ion milling was carried out at 5 kV acceleration voltage, and the produced thin foils were thinner than 50 nm.

### Materials Characterization—Transmission Electron Microscopy (TEM)

TEM analysis of FIB foils lifted out of the arc‐melted and annealed Nb‐Si‐Ni alloy samples was performed with a FEI Tecnai TF30 and a FEI Titan 80/300, both operated at 300 kV. The latter was equipped with an EDAX Octane Elite T EDS detector, whilst quantitative EDS analysis was done based on the Cliff‐Lorimer method.^[^ ^75^, ^76^ ^]^ Selected area electron diffraction patterns (SAEDPs) of the Nb_3_SiNi_2_ ZIP phase were indexed using data from the Inorganic Crystal Structure Database (ICSD).^[^ ^48^ ^]^ The CrystalMaker, SingleCrystal, and CrystalDiffraction software packages were used to simulate electron diffraction patterns and aid the indexing of experimentally acquired SAEDPs.^[^ ^53^ ^]^

Atomically resolved TEM images of the Nb_3_SiNi_2_ and Ni_3_SiNb_2_ ZIP phases were acquired by means of high‐angle annular dark‐field scanning transmission electron microscopy (HAADF STEM) on a double Cs‐corrected FEI Titan^3^ 60‐300 operated at 300 kV and equipped with a Super‐X EDS system.

A Thermo Fisher Scientific Talos F200X S/TEM operating at 200 keV was used for HAADF STEM imaging of FIB foils from the RHP Nb‐Si‐Co, Ta‐Si‐Ni, and V‐Si‐Ni alloy samples. STEM/EDS elemental maps were acquired using the Super‐X EDS system in the same TEM.

### Materials Characterization—Atom Probe Tomography (APT)

FIB (FEI Helios Nanolab 660 dual‐beam FIB/SEM) was employed to make needle‐shaped samples from the Nb‐Si‐Ni intermetallic alloy for further inspection by means of atom probe tomography (APT). The APT samples were made following a standard protocol^[^ ^77^ ^]^ using gallium (Ga) ions at 30 kV on the FIB. The site‐specific sample lift‐out was guided by EDS (EDAX Octane Elect detector; 5 kV acceleration voltage, 1.6 nA current). Final annular milling of the needle‐shaped specimens was done at 5 kV and 40 pA for 9 min to achieve a radius at the apex of ∼40 nm and a shank angle of ∼30°. Individual steps of APT specimen preparation are presented in Figure S6 (Supporting Information).

The composition of the Nb‐Si‐Ni intermetallic alloy was spatially‐resolved with APT, using voltage pulsing in a CAMECA local electrode atom probe (LEAP) 4000X HR. The pulse fraction, pulse rate, base temperature, and detection rate were 20%, 125 kHz, 60 K, and 0.5%, respectively. At least 15 × 10^6^ ions were collected, data analysis was carried out with the AP Suite 6.1 software (CAMECA, Madison, WI, USA), and the shank angle protocol was used for reconstructions.

### Materials Characterization—Thermal, Electrical, and Magnetic Properties

The Nb‐Si‐Ni intermetallic alloy samples were cut into rectangular parallelepipeds of 2 × 2 × 8 mm^3^. Electrical resistivity, *ρ*, measurements were performed with a four‐probe technique, using the BRT (Bridge, Rotator, and Tq‐Mag) option of the physical properties measurement system (PPMS; Quantum Design, San Diego, CA, USA). A current of 8 mA was applied to reach a resistance of ≈1 mΩ at RT. The temperature was reduced slowly from RT to 5 K, thus measuring continuously the electrical resistivity. The thermal conductivity, *κ*, and the Seebeck coefficient, *S*, were measured using the TTO (Thermal Transport Option) system of the PPMS. The TTO system measures thermal conductivity by applying heat from the heater shoe, creating a user‐specified temperature differential between the two thermometer shoes. The TTO system models dynamically the thermal response of the sample to a low‐frequency, square‐wave heat pulse, thus expediting data acquisition. The TTO system can then calculate thermal conductivity directly from the applied heater power (∼50 mW at RT), the resulting ΔT (∼10% of the temperature of the sample), and the sample geometry. With respect to the Seebeck coefficient, the voltage drop created between the two thermometer shoes was also monitored.

Molar heat capacity, *c_V_*, measurements were conducted on Nb‐Si‐Ni intermetallic alloy samples as a function of temperature in the 2‐325 K range, using a Quantum Design PPMS Dyna Cool measurement system. Fitting a Debye‐Einstein model (Equation 1) to the molar heat capacity curves of Nb_3_SiNi_2_ and Ni_3_SiNb_2_ yielded the Debye (θ_*D*_) and Einstein (θ_*E*_) temperatures, as well as the Sommerfeld coefficient, *γ*, for each ZIP phase:

(1) $$c_{V}=\gamma T+9NR\left(1-\alpha \right){\left(\frac{T}{\theta_{D}}\right)}^{3}\int_{0}^{\frac{\theta_{D}}{T}}\frac{x^{4}e^{x}}{{\left(e^{x}-1\right)}^{2}}dx+3NR\alpha \frac{{\left(\frac{\theta_{E}}{T}\right)}^{2}e^{\frac{\theta_{E}}{T}}}{{\left(e^{\frac{\theta_{E}}{T}}-1\right)}^{2}}$$

where *T* is the temperature of the sample, *α* is the number of Einstein modes, *N* = 6 is the number of atoms per formula unit (i.e., Nb_3_SiNi_2_ and Ni_3_SiNb_2_), and *R* = 8.314 J/(mol K).

Temperature‐ and applied‐magnetic‐field‐dependent magnetization measurements were conducted on Nb‐Si‐Ni intermetallic alloy samples, using the Quantum Design MPMS3 measurement system equipped with a He3 refrigerator. Temperature‐dependent DC magnetic susceptibilities, *χ(T)*, were obtained for both Nb_3_SiNi_2_ and Ni_3_SiNb_2_ ZIP phases from *z*ero‐field‐cooled (ZFC) magnetization measurements at low magnetic fields, *H*, of 10 Oe and 100 Oe, as well as at a high magnetic field of 70 kOe (equal to 7 T). Field‐dependent magnetization measurements, *m(H)*, were carried out at 1.8 K, 50 K, 100 K, 200 K, and 300 K. Moreover, the temperature‐dependent magnetic moment, *m(T)*, was measured between 0.4 K and 2 K for the Nb_3_SiNi_2_ ZIP phase at an applied magnetic field of 20 Oe under ZFC and field‐cooled (FC) conditions.

### Materials Characterization—Other Properties

Density was determined on the smaller RHP discs (∅ 30 mm) of Nb_3_SiNi_2_ and Ni_3_SiNb_2_ based on Archimedes' principle. The larger RHP disc (∅ 56 mm) of Nb_3_SiNi_2_ was cut into rectangular bars (43 × 3 × 2.3 mm^3^) that were polished prior to testing for mechanical and physical property determination. Vickers hardness (*H_V_*) was determined using an indentation load of 3 kg for 15 s (Future Tech model FV700 indenter). Four‐point bending (4 PB) testing was employed to determine flexural strength (σ_*f*_); five rectangular bars (43 × 3 × 2.3 mm^3^) were tested on an Instron 4467 test machine with fixed cross‐head displacement speed (0.5 mm/min) and fixed span length (20 mm inner span, 40 mm outer span). Dynamic elastic properties (Young's modulus, damping) were determined with the help of the impulse excitation technique (IET; GrindoSonic MK).

### Thermodynamic and Crystal Structure Simulations

Thermodynamic simulations were performed to predict the equilibrium phases at the sintering temperatures of the RHP Nb‐Si‐Ni alloys (i.e., 1523 K, 1623 K, and 1723 K), as well as at 1423 K (i.e., close to the homogenization annealing temperature, 1421 K, of the arc‐melted alloy). These simulations were performed with version V2020a of the Thermo‐Calc software (Thermo‐Calc Software Inc., Solna, Sweden),^[^ ^78^ ^]^ based on the database published by dos Santos et al.^[^ ^45^ ^]^ The unit cells of the ZIP phases in the Nb‐Si‐Ni system and the unit cell of the Ti_3_SiC_2_ MAX phase were drawn using version 3.5.8 of the VESTA software^[^ ^54^ ^]^ and utilizing the Wyckoff parameters provided in Table S3 (Supporting Information) for precise crystal structure representation. The two ZIP phases (i.e., Nb_3_SiNi_2_ and Ni_3_SiNb_2_) in the Nb‐Si‐Ni intermetallic alloy were compared in terms of unit cell sizes and structural complexity, whilst the crystal structure of the hexagonal Ni_3_SiNb_2_ ZIP phase variant was further compared to the (hexagonal) Ti_3_SiC_2_ MAX phase.

### Computational Methodology—Energy Minimization Calculations of Bulk Phases

Non‐spin polarized calculations were run using the Vienna Ab initio Simulation Package (VASP 5) code.^[^ ^79^, ^80^ ^]^ It employs the frozen‐core projector‐augmented wave (PAW) method^[^ ^81^, ^82^ ^]^ and a plane‐wave basis set with a 650‐eV cutoff, which was checked for convergence. The electron configurations were Si (2s^2^2p^2^), Ni (4d^8^4s^2^), and Nb (4s^2^4p^6^4d^3^5s^2^). 3D boundary conditions were used. The exchange correlation functional was the Perdew‐Burke‐Ernzerhof (PBE) generalized gradient approximation (GGA).^[^ ^83^ ^]^ All models were generated using the METADISE code.^[^ ^84^ ^]^ The full bulk 12‐atom unit cell for the hexagonal Ni_3_SiNb_2_, and the 96‐atom unit cell for the *fcc* Nb_3_SiNi_2_ were simulated with electronic and ionic convergence criteria of 1 × 10^−9^ eV and 1 × 10^−3^ eV Å^−1^, with the Brillouin zone sampled using Γ‐centered 5 × 5 × 3 and 4 × 4 × 4 k‐point meshes, respectively. The k‐point meshes were checked for convergence. To check the stability of any binary compounds that could arise from the removal of one of the elements in the ternary IMCs, the hexagonal Ni_3_Si, Ni_3_Nb_2_, SiNb_2,_ and *fcc* Nb_3_Si, Nb_3_Ni_2_, SiNi_2_ were simulated using the same settings as for the ternary IMCs. The Phonopy code^[^ ^85^ ^]^ was used to calculate second‐order harmonic interatomic force constants using the finite‐displacement method, with the VASP 5 code used as the force calculator. The phonon dispersions of all the stoichiometric structures were determined using a transformation matrix to the primitive cell, as implemented in the Phonopy code. The phonon dispersions of all bulk models and the atom‐projected phonon density of states (PDOS) curves for the stoichiometric cells were calculated on 16 × 16 × 16 and 30 × 30 × 30 Γ‐centered q‐point grids for the *fcc* and hexagonal structures, respectively.

### Computational Methodology—Energy Minimization Calculations of Surfaces

All surface models were generated using the METADISE code. They were modelled using the slab method, according to which two identical surfaces were created. A vacuum gap of 15 Å perpendicular to the surface was introduced to minimize the interaction between images. The surfaces were simulated with electronic and ionic convergence criteria of 1 × 10^−5^ eV and 1 × 10^−2^ eV Å^−1^, with the Brillouin zone sampled using a Γ‐centered k‐point mesh of 3 × 3 × 1 and 4 × 6 × 1 for the hexagonal (001) and (1$\bar{1}$0) Ni_3_SiNb_2_ surfaces, and 2 × 2 × 1 for the *fcc* Nb_3_SiNi_2_ surfaces, with the third vector perpendicular to the surface plane. For the hexagonal Ni_3_SiNb_2_, the (001) and (1$\bar{1}$0) surfaces (planes) were chosen as representative models of possible etching of the material. The (001) models were a (2 × 2) in‐plane expansion of the full surface unit cell. For the (001), the thickness of the slab was 2, 4, and 6 surface repeat units with 48 (8 Ni_3_SiNb_2_ units), 96 (16 Ni_3_SiNb_2_ units), and 144 (24 Ni_3_SiNb_2_ units) atoms, respectively. A surface with 1 repeat surface unit could not be stabilized for the (001) surface. To remove the dipole of the (001) slab, half of the surface Si atoms were moved from one side to the other of the slab, generating highly symmetric surface models. The (1$\bar{1}$0) models were a (1 × 1) in‐plane expansion of the full surface unit cell. For the (1$\bar{1}$0), the thickness of the slab was 1, 2, and 3 surface repeat units with 12 (2 Ni_3_SiNb_2_ units), 24 (4 Ni_3_SiNb_2_ units), and 36 (6 Ni_3_SiNb_2_ units) atoms, respectively. For the *fcc* Nb_3_SiNi_2_, the (010) and (101) surfaces (planes) were chosen as representative models of possible etching of the material. Both models were a (1 × 1) in‐plane expansion of the primitive surface unit cell. For both surfaces, the thickness of the slab was 1, 2, and 3 surface repeat units with 24 (4 Nb_3_SiNi_2_ units), 48 (8 Nb_3_SiNi_2_ units), and 72 (12 Nb_3_SiNi_2_ units) atoms, respectively. Two different terminations were used for the (010), and to remove the dipole, half of the surface Nb and Si atoms, respectively, were moved from one side to the other of the slab, generating highly symmetric surface models. To remove the dipole of the (101) slab, half of the surface Si atoms were moved from one side to the other of the slab, generating highly symmetric surface models. The surface energy was calculated as γ_*surf*_ = (*E_surf_* − *E_bulk_*)/2*A*, where *E_surf_* and *E_bulk_* are the energies of the surface model and the bulk structure with the same number of units as the surface model, respectively; *A* is the surface area; and 2 accounts for the usage of the slab method.

### Computational Methodology—Bond Analysis

Hybrid density functional theory (DFT) was also performed, using the Heyd‐Scuseria‐Ernzerhof (HSE06) functional^[^ ^86^, ^87^ ^]^ for the geometry optimization of Ni_3_SiNb_2_, computed with a mesh of 5 × 5 × 3 k‐points, and the electronic density of states (eDOS) was computed with a mesh of 8 × 8 × 4 k‐points. The structure was simulated with electronic and ionic convergence criteria of 1 × 10^−5^ eV and 1 × 10^−2^ eV Å^−1^. The Bader analysis was performed by partitioning the hybrid DFT core and valence charge density grids.^[^ ^88^, ^89^ ^]^ The critical points (CP) and the non‐covalent interactions (NCI) of the hybrid DFT charge densities were computed using the Critic2 code.^[^ ^90^, ^91^ ^]^

### Computational Methodology—Temperature‐Dependent Phonon Dispersion

The phonon dispersion relations were determined at finite temperatures from the ab initio molecular dynamics (AIMD) simulations using the temperature‐dependent effective potential (TDEP) method. The TDEP method was explained in detail elsewhere.^[^ ^92^, ^93^ ^]^ The basis of the TDEP method was to obtain the interatomic force constant matrices at finite‐temperatures from the AIMD together with Hooke's law, provided the structure has a valid symmetry. It was necessary to use a large supercell to achieve the force convergence for TDEP. Therefore, a supercell with 324 atoms for hexagonal Ni_3_SiNb_2_ (a 3 × 3 × 3 expansion of the full unit cell) and a supercell with 768 atoms for *fcc* Nb_3_SiNi_2_ (a 2 × 2 × 2 expansion of the full unit cell) were used to run the AIMD simulations. Similarly, we constructed supercells for the binary compounds with atoms 270 for hexagonal Ni_3_Nb_2_, 216 for hexagonal Ni_3_Si, 162 for hexagonal SiNb_2_, 640 for *fcc* Nb_3_Ni_2_, 512 for *fcc* Nb_3_Si, and 384 for *fcc* SiNi_2_, respectively. The lattice parameters for the supercells were adopted from the 0 K optimized structure of the corresponding compounds. The AIMD simulations were carried out using the VASP code with identical pseudopotentials (electron configurations) and exchange‐correlation functionals, as described earlier. However, the Brillouin zone integration was performed using the Γ point of the supercell. The temperature of both ternary compounds was set to 1723 K during the AIMD simulations, as they were found to be stable at this elevated temperature and in agreement with the experiments. However, all the binary compounds were simulated at 300 K except hexagonal SiNb_2_, which was simulated at 600 K. A Nosé‐Hoover thermostat was used to control the temperature. Since a very large simulation box was chosen, the simulations were run about 2000 steps for *fcc* structures and 5000 for hexagonal structures with a time step of 2 fs to obtain the converged forces.

## Conflict of Interest

The authors declare no conflict of interest.

## Supporting information



Supporting Information

## Data Availability

The data that support the findings of this study are available from the corresponding author upon reasonable request.
