# Supplementary material for: A New Family of Ternary Intermetallic Compounds with Dualistic Atomic Ordering – The ZIP Phases
Source: Adv Mater. 2025 Sep 10;38(8):e08168. doi: 10.1002/adma.202308168 (PMC12878816; doi:10.1002/adma.202308168)
Supplement: Supplementary file 1 — Supporting Information [file ADMA-38-e08168-s001.docx]

Supporting Information

A new family of ternary intermetallic compounds with dualistic atomic ordering – The ZIP phases

Matheus A. Tunes*, Sean M. Drewry, Franziska Schmidt, James A. Valdez, Matthew M. Schneider, Caitlin A. Kohnert, Tarik A. Saleh, Saryu Fensin, Stuart A. Maloy, Cláudio G. Schön, Sylvain Dubois, Omri Tabo, Anna Eyal, Amit Keren, Asaf Pesach, Ganesh K. Nayak, Stavros-Richard G. Christopoulos, Marco Molinari, Marcus Hans, Nick Goossens, Shuigen Huang, Jochen M. Schneider, Per O.Å. Persson, Jozef Vleugels, Konstantina Lambrinou*

**S1. Synthesis of ZIP Phases in the Nb-Si-Ni System**

This section provides additional information on the chemical composition (as determined by SEM/EDS analysis) of Nb-Si-Ni alloy samples produced by arc melting and RHP (Table S1). It also provides additional SEM micrographs showing the microstructures of both as-cast and annealed Nb-Si-Ni alloy samples synthesized by means of arc melting (Figure S1). Furthermore, it provides additional SEM micrographs and WDS elemental maps of quasi phase-pure Nb-Si-Ni alloy samples synthesized by reactive hot pressing (RHP) at three different sintering temperatures, i.e., 1523 K, 1623 K, and 1723 K (Figure S2).

**Table S1.** Nominal stoichiometries versus actual compositions of the phases identiﬁed by SEM/EDS and EPMA/WDS in the arc-melted (as-cast & annealed) and RHP Nb-Si-Ni alloy samples, respectively. The experimentally determined phase compositions are compared with the nominal phase compositions, as determined by their respective stoichiometries.

| **Arc-melted Nb-Si-Ni intermetallic alloy samples** | | | | | | | | | |
| --- | --- | --- | --- | --- | --- | --- | --- | --- | --- |
| **Phases** | | **Stoichiometry** | | **Nb [at.%]** | | **Si [at.%]** | | **Ni [at.%]** | |
| H-phase (as-cast) | | Nb_3_SiNi_2_  (*fcc* ZIP phase) | | 46.5±3.2 | | 18.4±4.0 | | 35.1±1.9 | |
| H-phase (annealed) | |  |  | 46.4±3.0 | | 18.6±3.7 | | 35.0±1.5 | |
| **H-phase (nominal)** | |  |  | **50.0** | | **17.0** | | **33.0** | |
| Laves phase (as-cast) | | Ni_3_SiNb_2_  (hexagonal ZIP phase) | | 31.9±2.7 | | 21.8±3.5 | | 46.3±1.8 | |
| Laves phase (annealed) | |  |  | 31.3±2.4 | | 20.1±3.1 | | 48.6±1.2 | |
| **Laves phase (nominal)** | |  |  | **33.0** | | **17.0** | | **50.0** | |
| µ-phase (as-cast) | | Nb_7_Ni_6_^a)^ | | 45.2±3.1 | | 13.5±3.8 | | 41.3±1.9 | |
| µ-phase (annealed) | |  |  | 43.8±3.0 | | 13.5±3.7 | | 42.7±1.5 | |
| **µ-phase (nominal)** | |  |  | **53.8** | | **-** | | **46.2** | |
| T-phase (as-cast) | | Nb_4_NiSi | | 62.8±3.8 | | 25.8±4.7 | | 11.4±2.5 | |
| T-phase (annealed) | |  |  | 62.6±3.8 | | 25.3±4.7 | | 12.0±1.9 | |
| **T-phase (nominal)** | |  |  | **66.0** | | **17.0** | | **17.0** | |
| **RHP Nb-Si-Ni intermetallic alloy samples** | | | | | | | | | |
| **Sintering T [K]** | **Phases** | | **Nb [at.%]** | | **Si [at.%]** | | **Ni [at.%]** | | **O [at.%]** |
| 1523 | H-Nb_3_SiNi_2_ | | 53.86±0.16 | | 12.71±0.03 | | 33.43±0.12 | | - |
|  | L-Ni_3_SiNb_2_ | | 38.6±0.12 | | 17.63±0.03 | | 43.77±0.12 | | - |
|  | µ-Nb_7_Ni_6_ | | 71.99±0.13 | | 17.17±0.03 | | 10.85±0.07 | | - |
| 1623 | H-Nb_3_SiNi_2_ | | 53.57±0.15 | | 13.26±0.03 | | 33.17±0.12 | | - |
|  | L-Ni_3_SiNb_2_ | | 36.25±0.15 | | 17.49±0.04 | | 46.27±0.16 | | - |
|  | µ-Nb_7_Ni_6_ | | - | | - | | - | | - |
| 1723 | H-Nb_3_SiNi_2_ | | 52.8±0.17 | | 13.12±0.04 | | 34.08±0.14 | | - |
|  | L-Ni_3_SiNb_2_ | | 36.32±0.11 | | 17.47±0.03 | | 46.21±0.11 | | - |
|  | µ-Nb_7_Ni_6_ | | 65.96±0.21 | | 8.43±0.04 | | 4.65±0.09 | | 20.96±0.05 |

^a)^Si was reported to dissolve in the binary Nb_7_Ni_6_ IMC, forming µ-phase solid solutions.^[47]^


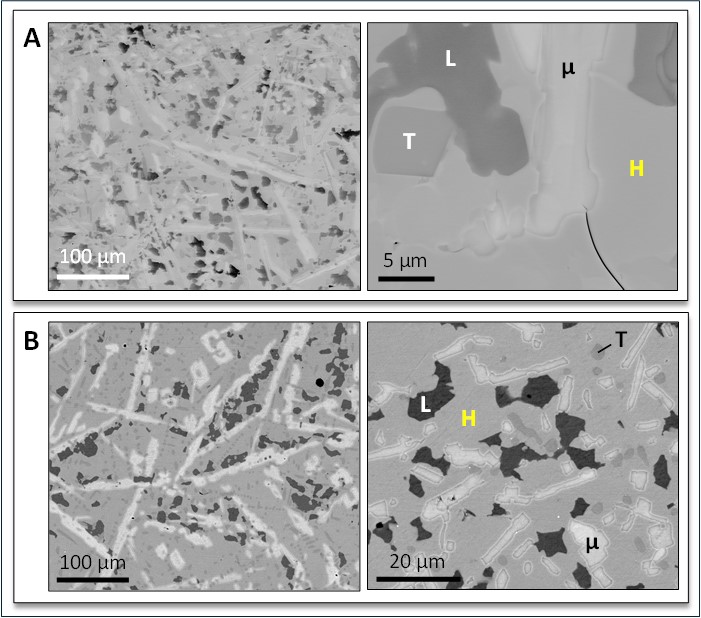


**Figure S1.** SEM imaging of as-cast (A) and annealed (B) arc-melted Nb-Si-Ni alloy samples. Both Nb-Si-Ni alloy samples are phase mixtures comprising the same four distinct phases, i.e., H-Nb3SiNi2, L-Ni3SiNb2, μ-Nb7Ni6 and T-Nb4SiNi.


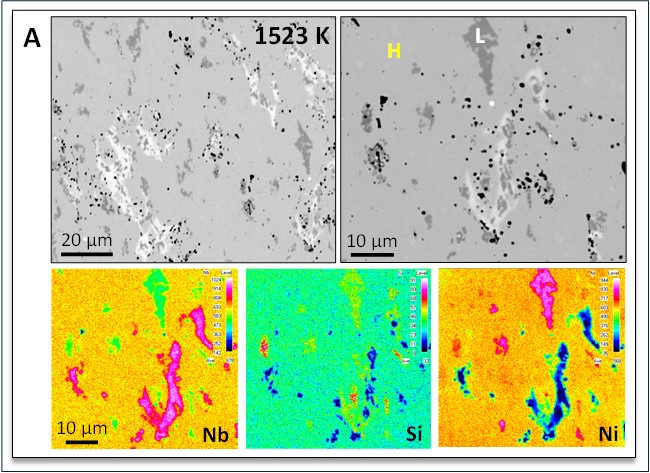


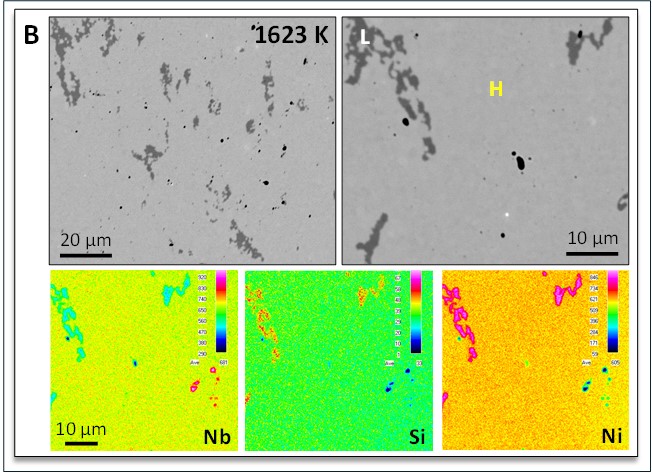


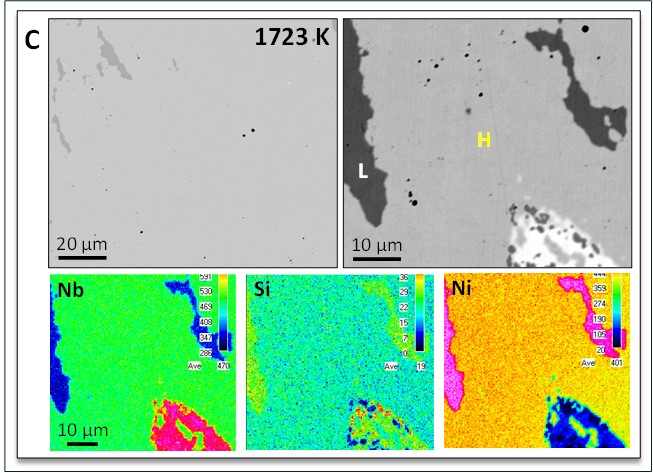


**Figure S2.** EPMA imaging and WDS elemental mapping of RHP Nb-Si-Ni alloy samples sintered at 1523 K (A), 1623 K (B), and 1723 K (C). All samples comprise two main phases, i.e., H-Nb_3_SiNi_2_ and L-Ni_3_SiNb_2_, as well as NbO_2_ and NiO impurities, the presence of which was confirmed by XRD.

One of the quasi phase-pure RHP Nb-Si-Ni alloy samples sintered at 1723 K was milled and the produced powder was analyzed by XRD, followed by full Rietveld refinement of the XRD pattern. The Rietveld-refined powder XRD pattern is shown in Figure S3, whereas Table S2 summarizes in tabular form the results of the Rietveld refinement in terms of phase assembly (including the ‘impurity phases’ NbO_2_, SiO_2_ and Si); lattice parameters; atomic site positions, occupancies and displacements; and temperature factors.


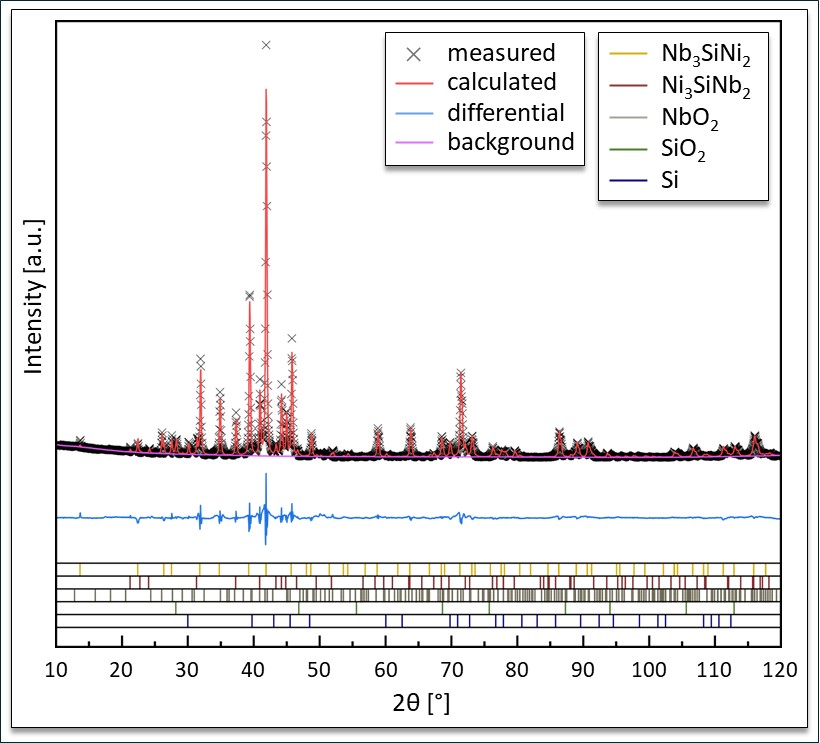


**Figure S3.** Rietveld-refined powder XRD pattern of an RHP Nb-Si-Ni alloy sample sintered at 1723 K. The main phases in this sample were the H-Nb_3_SiNi_2_ and L-Ni_3_SiNb_2_ ternary IMCs; traces of oxidic inclusions (SiO_2_, NbO_2_) and residual silicon (Si) were also detected.

**Table S2.** Results of full Rietveld refinement of the powder XRD pattern of an RHP Nb-Si-Ni sample sintered at 1723 K: lattice parameters of all phases; atomic site positions, occupancies & displacements; and temperature factors B_iso_ (assuming displacement spheroids).

| **Phase** | | **Nb_3_SiNi_2_** | | **Ni_3_SiNb_2_** | | **SiO_2_** | | **NbO_2_** | | **Si** | |
| --- | --- | --- | --- | --- | --- | --- | --- | --- | --- | --- | --- |
| PDF-code | | 01-072-2171 | | 04-008-0835 | | 01-076-2975 | | 04-005-4690 | | 04-014-0211 | |
| Fraction [wt.%] | | 65.2 | | 26.1 | | 5.1 | | 2.6 | | 1.0 | |
| Space group | | $\text{Fd}\bar{\text{3}}\text{m}$ (227) | | *P6_3_/mmc* (194) | | *I4_2_/mnm* (136) | | *I4_1_/a* (88) | | $\text{Fd}\bar{\text{3}}\text{m}$ (227) | |
| *a* [Å] | | 11.1981 | | 4.8139 | | 4.1895 | | 13.654 | | 5.465 | |
| *c* [Å] | |  | | 7.7842 | | 2.688 | | 6.024 | |  | |
| *V* [Å^3^] | | 1404.21 | | 156.22 | | 47.18 | | 1123.06 | | 163.22 | |
| Theoretical density [g/cm^3^] | | 8.060 | | 7.963 | | 4.229 | | 5.909 | | 2.286 | |
| Discrepancy indices | | R_wp_ = 2.84% | | | R_exp_ = 1.81% | | | | *χ^2^* = 2.46 | | |
|  |  | **Structural refinement of *fcc* Nb_3_SiNi_2_** | | | | | | | | | |
|  | Element | Wyckoff site | *x* | | *y* | | *z* | | Occupancy | | B_iso_ |
| Nb | | 48f | 0.1974 | | 0 | | 0 | | 1 | | 0.0067 |
| Si | | 16d | 5/8 | | 5/8 | | 5/8 | | 1 | | 0.003 |
| Ni | | 32e | 0.8316 | | 0.8316 | | 0.8316 | | 1 | | 0.0069 |
| O | | 16c | 1/8 | | 1/8 | | 1/8 | | 0.11 | | 0.01 |
|  |  | **Structural refinement of hexagonal Ni_3_SiNb_2_** | | | | | | | | | |
|  | Element | Wyckoff site | *x* | | *y* | | *z* | | Occupancy | | B_iso_ |
| Nb | | 4f | 1/3 | | 2/3 | | 0.5599 | | 1 | | 0.014 |
| Si 1 | | 2a | 0 | | 0 | | 0 | | 0.67 | | 0 |
| Ni 1 | | 2a | 0 | | 0 | | 0 | | 0.33 | | 0 |
| Si 2 | | 6h | 0.160 | | 0.3394 | | 1/4 | | 0.28 | | 0.02 |
| Ni 2 | | 6h | 0.160 | | 0.3394 | | 1/4 | | 0.72 | | 0.02 |
|  |  | **Structural refinement of SiO_2_** | | | | | | | | | |
| Element | | Wyckoff site | *x* | | *y* | | *z* | | Occupancy | | B_iso_ |
| Si | | 2a | 0 | | 0 | | 0 | | 1 | | 0.0017 |
| O | | 4f | 0.3561 | | 0.3561 | | 0.3561 | | 1 | | 0.0022 |
|  |  | **Structural refinement of NbO_2_** | | | | | | | | | |
|  | Element | Wyckoff site | *x* | | *y* | | *z* | | Occupancy | | B_iso_ |
| Nb 1 | | 16f | 0.1038 | | 0.1075 | | 0.522 | | 1 | | 0.0420 |
| Nb 2 | | 16f | 0.1174 | | 0.1207 | | 0.016 | | 1 | | 0.0368 |
| O 1 | | 16f | 0.95 | | 0.096 | | 1 | | 1 | | 0.0435 |
| O 2 | | 16f | 0.95 | | 0.093 | | 0.48 | | 1 | | 0.0448 |
| O 3 | | 16f | 0.257 | | 0.113 | | 1 | | 1 | | 0.0361 |
| O 4 | | 16f | 0.259 | | 0.101 | | 0.5 | | 1 | | 0.0361 |
|  |  | **Structural refinement of Si** | | | | | | | | | |
|  | Element | Wyckoff site | *x* | | *y* | | *z* | | Occupancy | | B_iso_ |
| Si | | 8a | 1/8 | | 1/8 | | 1/8 | | 1 | | 0.01 |

**S2. Characterization of ZIP Phases in the Nb-Si-Ni System by TEM and APT**

This section presents the findings of the characterization of the (low phase-purity) Nb-Si-Ni alloy sample by means of STEM/EDS/SAED; the analyzed sample was synthesized by arc melting and annealed at 1421 K for 336 h. Figure S4 shows the results of STEM/EDS analysis on a large Nb_3_SiNi_2_ grain found in the annealed arc-melted Nb-Si-Ni alloy sample, whilst Figure S5 provides the results of STEM/SAED characterization of the same grain. These analyses showed that the composition of the analyzed grain was homogeneous and close to the Nb_3_SiNi_2_ stoichiometry; moreover, the crystal structure of Nb_3_SiNi_2_ was face-centered cubic (*fcc*) diamond cubic, space group *Fd*$\bar{3}$*m* (space group, SG, 227).

**
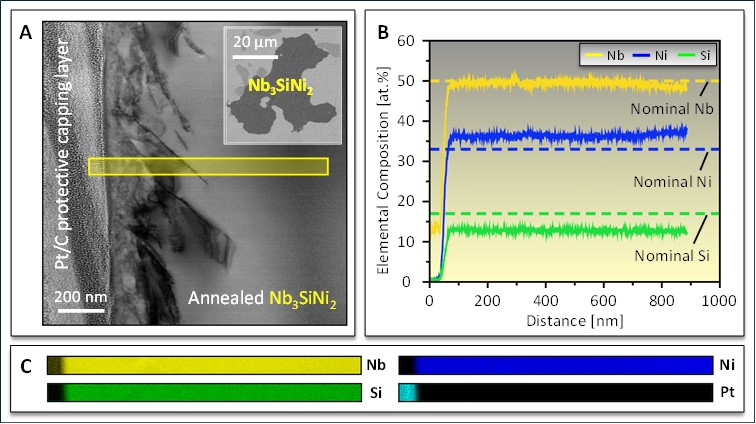
**

**Figure S4.** STEM/EDS analysis of a large Nb_3_SiNi_2_ grain in the annealed arc-melted Ni-Si-Ni alloy sample. (A) BF-STEM micrograph of the Nb_3_SiNi_2_ grain shows shallow (<500 nm) sub-surface damage induced during sample preparation. A combined Pt/C strip was used to protect the area of interest during FIB lift-out. The inset BSE image shows the analyzed Nb_3_SiNi_2_ grain. STEM/EDS analysis of the area of interest (yellow rectangle, Figure S4A) reveals the compositional homogeneity of the grain, as depicted in both elemental profiles (B) and maps (C). The average grain composition was found to deviate only slightly from the Nb_3_SiNi_2_ stoichiometry, i.e., by about 5 at.% excess Ni, and 5 at.% Si deﬁciency.

**
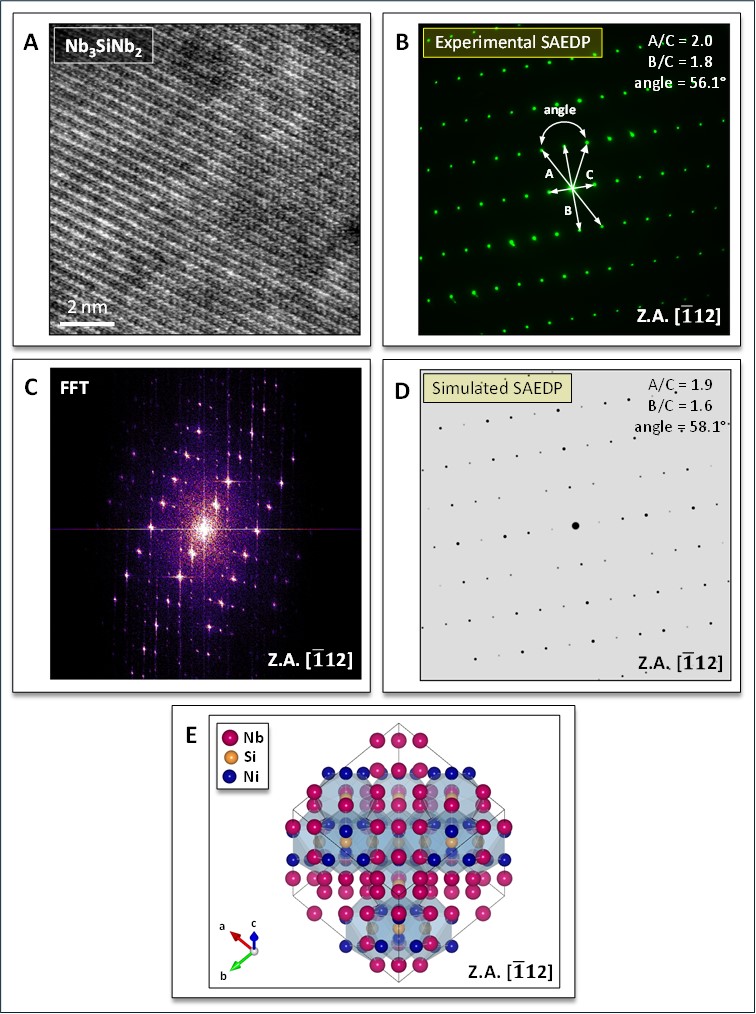
**

**Figure S5.** STEM/SAED characterization of the Nb_3_SiNi_2_ grain in Figure S4A. (A) BF-STEM micrograph of Nb_3_SiNi_2_ viewed along the low-index [$\bar{1}$12] Z.A. (B) Experimental SAEDP and (C) Fast Fourier Transform (FFT) pattern of the [$\bar{1}$12] Z.A. of Nb_3_SiNi_2_. (D) Simulated SAEDP showing the [$\bar{1}$12] Z.A. of Nb_3_SiNi_2_. (E) Schematic representation of the [$\bar{1}$12] Z.A. of Nb_3_SiNi_2_. The simulated SAEDP was produced by SingleCrystal,^[53]^ using experimental data found in the ICSD database.^[48]^ Both experimental and simulated SAEDPs conﬁrm that Nb_3_SiNi_2_ has the diamond cubic (*fcc*) crystal structure (*Fd*$\bar{3}$*m*, SG 227).

**Table S3.** Crystal structure data for the *fcc* Nb_3_SiNi_2_ ZIP phase, the hexagonal Ni_3_SiNb_2_ ZIP phase, and the Ti_3_SiC_2_ MAX phase, together with the Wyckoff parameters used to draw the unit cells of these three phases in Figure 4.

| **Crystal structure** | | **Ti_3_SiC_2_**  **MAX phase** | | **Nb_3_SiNi_2_**  **ZIP phase** | | **Ni_3_SiNb_2_**  **ZIP phase** | |
| --- | --- | --- | --- | --- | --- | --- | --- |
| Space group | | *P6_3_/mmc* (SG 194) | | *Fd*$\bar{3}$*m* (SG 227) | | *P6_3_/mmc* (SG 194) | |
| *a* [Å] | | 3.07 | | 11.22 | | 4.73 | |
| *c* [Å] | | 17.69 | | 11.22 | | 7.49 | |
| Unit cell volume [Å^3^] | | 144 | | 1411 | | 145 | |
| Atoms per unit cell | | 6 | | 96 | | 12 | |
| Theoretical density [g/cm^3^] | | 4.50 | | 7.99 | | 7.94 | |
| **Ti_3_SiC_2_ MAX phase** | | | | | | | |
| **Element** | **Wyckoff position** | | ***x*** | | ***y*** | | ***z*** |
| Ti | 2a | | 0 | | 0 | | 1/2 |
|  | 4f | | 2/3 | | 1/3 | | 0.364919 |
| Si | 2b | | 0 | | 0 | | 3/4 |
| C | 4f | | 1/3 | | 2/3 | | 0.427507 |
| **Nb_3_SiNi_2_ ZIP phase (*fcc* variant)** | | | | | | | |
| **Element** | **Wyckoff position** | | ***x*** | | ***y*** | | ***z*** |
| Nb | 48f | | 1/2 | | 0.202507 | | 1/2 |
| Si | 16d | | 1/8 | | 5/8 | | 1/8 |
| Ni | 32e | | 0.33234 | | 0.83234 | | 0.33234 |
| **Ni_3_SiNb_2_ ZIP phase (hexagonal variant)** | | | | | | | |
| **Element** | **Wyckoff position** | | ***x*** | | ***y*** | | ***z*** |
| Nb | 4f | | 2/3 | | 1/3 | | 0.93993 |
| Si | 2a | | 0 | | 0 | | 0 |
| Ni | 6h | | 0.16888 | | 0.33775 | | 3/4 |

Table S3 compares crystal structure data (i.e., lattice parameters, unit cell volume, atoms per unit cell, theoretical density) of the two ZIP phases in the Nb-Si-Ni system (i.e., *fcc* Nb_3_SiNi_2_ & hexagonal Ni_3_SiNb_2_) with the (hexagonal) Ti_3_SiC_2_ MAX phase and provides the Wyckoff parameters used to draw the unit cells of these three phases in Figure 4. These data were retrieved from the ICSD database;^[48]^ hence, they differ slightly from the results of the Rietveld refinement of the RHP Nb-Si-Ni sample sintered at 1723 K (Table S2).


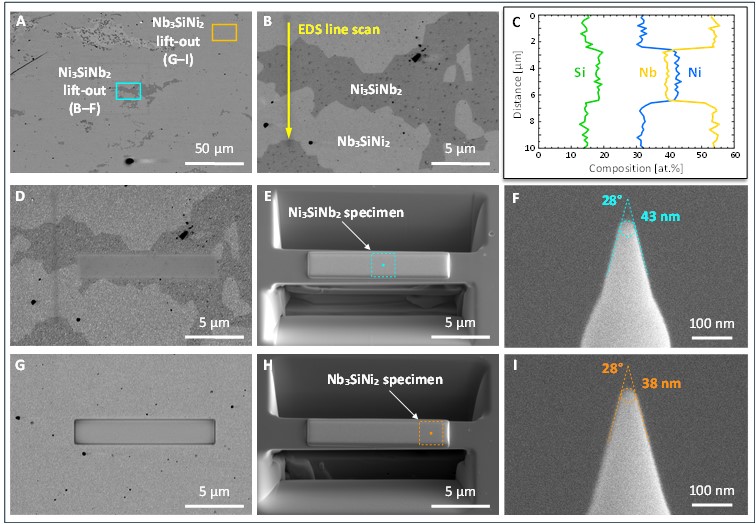


**Figure S6.** APT specimen preparation from an RHP Nb-Si-Ni alloy sample sintered at 1723 K by site-specific FIB lift-out. (A) Overview image. (B) Region of interest showing adjacent grains of Ni_3_SiNb_2_ and Nb_3_SiNi_2_. (C) EDS line-scan showing the Nb, Si and Ni profiles in the area of interest; as shown in Figure S6B, the EDS line-scan starts from a Nb_3_SiNi_2_ area, crosses a Ni_3_SiNb_2_ area, and ends in a Nb_3_SiNi_2_ area. (D) Deposition of a Pt capping layer for surface protection of Ni_3_SiNb_2_ during ion milling, (E) trench milling to form a wedge of material for FIB lift-out, and (F) the final Ni_3_SiNb_2_ atom probe specimen. (G) Deposition of a Pt capping layer for surface protection of Nb_3_SiNi_2_, (E) trench milling to form a wedge of material for FIB lift-out, and (F) the final Nb_3_SiNi_2_ atom probe specimen.

**Figure S7.** Mass spectrum of Nb_3_SiNi_2_. Apart from the principal elements Nb, Ni and Si, the presence of O is evident. The sum of C, Ta and W impurities is <1 at.%.

**Figure S8.** Mass spectrum of Ni_3_SiNb_2_. The sum of Ta and W impurities is <1 at.%.

Figure S6 presents successive steps in the preparation of APT specimens from Nb_3_SiNi_2_ and Ni_3_SiNb_2_ areas in an RHP Nb-Si-Ni sample sintered at 1723 K. Figure S7 and Figure S8 show the APT mass spectra for the Nb_3_SiNi_2_ and the ZIP phase, respectively. Figure S9 shows the APT reconstructions of the Nb, Ni, Si and O atomic positions in two neighboring Ni_3_SiNb_2_ and Nb_3_SiNi_2_ grains, revealing the presence of oxygen (O) in the Nb_3_SiNi_2_ grain and its absence from its neighboring Ni_3_SiNb_2_ grain.


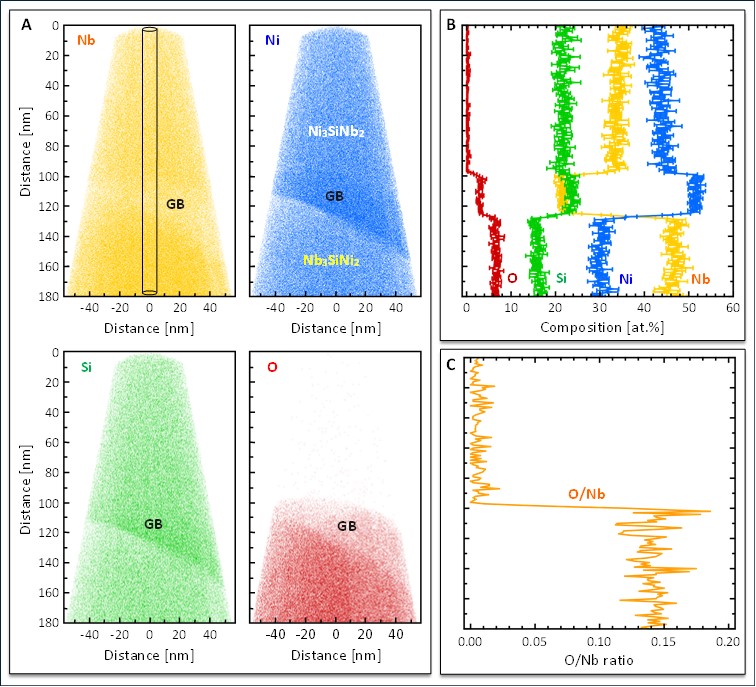


**Figure S9.** Spatially-resolved composition of Ni_3_SiNb_2_ and Nb_3_SiNi_2_ neighboring grains separated by a slanted grain boundary (GB), as determined by APT. (A) Reconstructions of the Nb, Ni, Si and O atomic positions in the adjacent Nb_3_SiNi_2_ and Ni_3_SiNb_2_ grains. Elemental profile (B) and Nb/O ratio (C) along a cylinder (∅ 10 nm) similar to that shown in the map of Nb atomic positions.

Figure S9B shows the composition profile of a cylindrical region (Figure S9A, map of Nb atomic positions), which can be divided into three regions. In the 0-100 nm distance range (Figure S9A), the average composition is 34±1 at.% Nb, 22±1 at.% Si, and 44±1 at.% Ni, whereas the O content is <1 at.%, corresponding to a stoichiometry of Ni_2.0_SiNb_1.6_. The average composition of a GB region located in the 100-130 nm distance range (Figure S9A) is 21±1 at.% Nb, 24±1 at.% Si, 52±1 at.% Ni, and 3 at.% O. In the 130-180 nm distance range (Figure S9A), the average composition is 47±1 at.% Nb, 16±1 at.% Si, 30±1 at.% Ni, and 7±1 at.% O. Neglecting the O impurities, a stoichiometry of Nb_2.9_SiNi_1.9_ is obtained, which is in excellent agreement with the nominal Nb_3_SiNi_2_ stoichiometry. Figure S9C shows that O incorporation in the Ni_3_SiNb_2_ and Nb_3_SiNi_2_ grains results in an average O/Nb ratio of 0.14±0.01. The results of Figure S9 prove beyond doubt that O dissolves in the *fcc* Nb_3_SiNi_2_ ZIP phase, but not in its hexagonal Ni_3_SiNb_2_ counterpart.

**S3. Computational Results**

*S3.1. Structure and Stability of Phases*

Table S4 shows a very minimal contraction for the *fcc* Nb_3_SiNi_2_ ZIP phase together with an underestimation of the lattice parameters for the hexagonal Ni_3_SiNb_2_ ZIP phase. As already mentioned, the lack of imaginary modes at the Γ-point or high-symmetry points in the phonon band structures of Nb_3_SiNi_2_ & Ni_3_SiNb_2_ at 0 K (Figure 9A & Figure 9B) and 1723 K (Figure 9C & Figure 9D) indicates thermodynamic and dynamic stability of both ternary IMCs in the 0-1723 K temperature range.

**Table S4.** Comparison of lattice parameters for the Nb_3_SiNi_2_ and Ni_3_SiNb_2_ ZIP phases and their potential 2D derivatives, calculated using DFT without the inclusion of temperature effects, and using experimental data, where available.

| **Structure** | **Methodology** | **Symmetry** | ***a* [Å]** | ***b* [Å]** | ***c* [Å]** | **α [°]** | **β [°]** | **γ [°]** |
| --- | --- | --- | --- | --- | --- | --- | --- | --- |
| **hexagonal Ni_3_SiNb_2_** | **Computational GGA** | *P6_3_/mmc*  (SG 194) | 4.8634 | 4.8634 | 7.6785 | 90.00 | 90.00 | 120.00 |
|  | **Computational HSE06** | *P6_3_/mmc*  (SG 194) | 4.87516 | 4.87516 | 7.53171 | 90.00 | 90.00 | 120.00 |
|  | **Experimental** | *P6_3_/mmc*  (SG 194) | 4.7279 | 4.7279 | 7.4882 | 90.00 | 90.00 | 120.00 |
| **hexagonal Ni_3_Si** | **Computational GGA** | *P6_3_/mmc*  (SG 194) | 4.8291 | 4.8291 | 6.8714 | 90.00 | 90.00 | 120.00 |
| **hexagonal Ni_3_Nb_2_** | **Computational GGA** | *P6_3_/mmc*  (SG 194) | 4.8314 | 4.8314 | 7.1923 | 90.00 | 90.00 | 120.00 |
| **primitive SiNb_2_** | **Computational GGA** | *P1* (SG 1) | 4.9356 | 4.9387 | 6.7778 | 110.08 | 110.00 | 121.78 |
| ***fcc* Nb_3_SiNi_2_** | **Computational GGA** | *Fd*$\overline{3}$*m*  (SG 227) | 11.2032 | 11.2032 | 11.2032 | 90.00 | 90.00 | 90.00 |
|  | **Experimental** | *Fd*$\overline{3}$*m*  (SG 227) | 11.2200 | 11.2200 | 11.2200 | 90.00 | 90.00 | 90.00 |
| ***fcc***  **Nb_3_Si** | **Computational GGA** | *Fd*$\overline{3}$*m*  (SG 227) | 10.7461 | 10.7461 | 10.7461 | 90.00 | 90.00 | 90.00 |
| ***fcc***  **Nb_3_Ni_2_** | **Computational GGA** | *Fd*$\overline{3}$*m*  (SG 227) | 10.9054 | 10.9054 | 10.9054 | 90.00 | 90.00 | 90.00 |
| ***fcc***  **SiNi_2_** | **Computational GGA** | *Fd*$\overline{3}$*m*  (SG 227) | 8.4171 | 8.4171 | 8.4171 | 90.00 | 90.00 | 90.00 |

Considering the stability of all binary compounds that could theoretically be derived from the ternary IMCs Nb_3_SiNi_2_ (Figure S10A,B,C) and Ni_3_SiNb_2_ (Figure S10D,E,F) without changing the elemental ratios in their nominal chemical compositions, only SiNb_2_ (binary derivative of hexagonal Ni_3_SiNb_2_) shows no imaginary modes at the Γ-point at 0 K (Figure S10E), however, its crystal structure has lost symmetry as compared to the parent hexagonal symmetry of Ni_3_SiNb_2_ (see Table S4).

Two of the binary compounds potentially derived from the stoichiometric *fcc* Nb_3_SiNi_2_ ZIP phase, i.e., Nb_3_Si (Figure S11A) and Nb_3_Ni_2_ (Figure S11B) do not melt at RT, whereas the candidate binary compound SiNi_2_ melts at RT (Figure S11C). Moreover, all derivative binary compounds of stoichiometric *fcc* Nb_3_SiNi_2_ show imaginary modes at RT, except for Nb_3_Si that appears to be stable (Figure S11A). On the other hand, two of the candidate binary compounds potentially derived from the stoichiometric hexagonal Ni_3_SiNb_2_ ZIP phase, i.e., Ni_3_Nb_2_ (Figure S11D) and Ni_3_Si (Figure S11F), melt at RT, as indicated by the very low frequencies in the phonon dispersions. Moreover, the binary compound SiNb_2_ (Figure S11E) does not melt up to 600 K, but shows imaginary modes at the Γ-point, suggesting thermodynamic instability at this temperature.

**
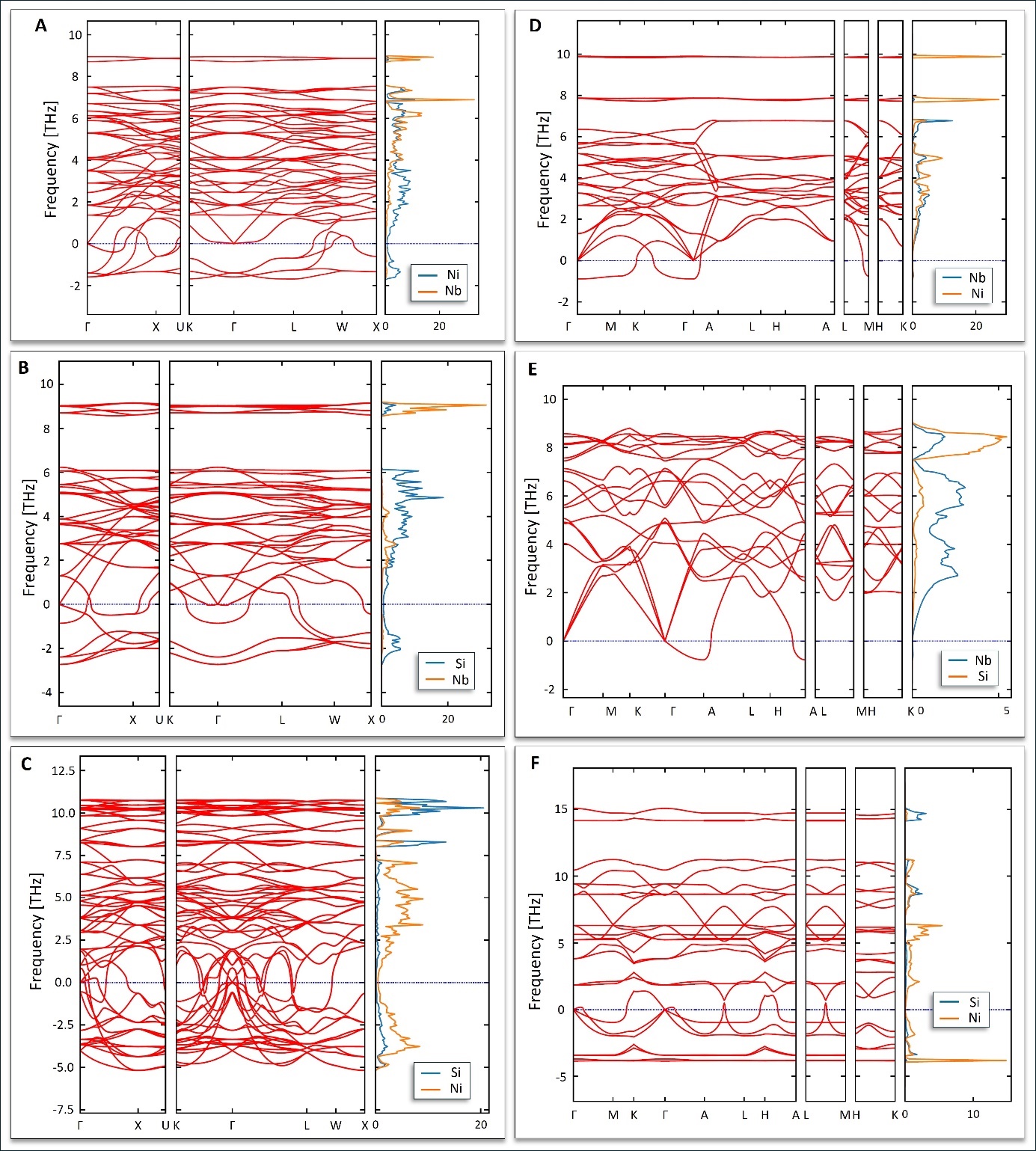
**

**Figure S10:** Predicted phonon dispersion and atom-projected PDOS without the inclusion of temperature effects (i.e., at 0 K) for the derivative binary compounds Nb_3_Ni_2_ (A), Nb_3_Si (B), and SiNi_2_ (C) of stoichiometric *fcc* Nb_3_SiNi_2_. None of the candidate binary compounds of Nb_3_SiNi_2_ appears to be thermodynamically stable at 0 K. Predicted phonon dispersion and atom-projected PDOS at 0 K for the derivative binary compounds Ni_3_Nb_2_ (D), SiNb_2_ (E), and Ni_3_Si (F) of stoichiometric hexagonal Ni_3_SiNb_2_. Only SiNb_2_ does not show any imaginary modes at the Γ-point from all candidate binary compounds of Ni_3_SiNb_2_, which indicates thermodynamic stability. However, the observed imaginary modes at high-symmetry points suggest dynamic instability of SiNb_2_ at 0 K.


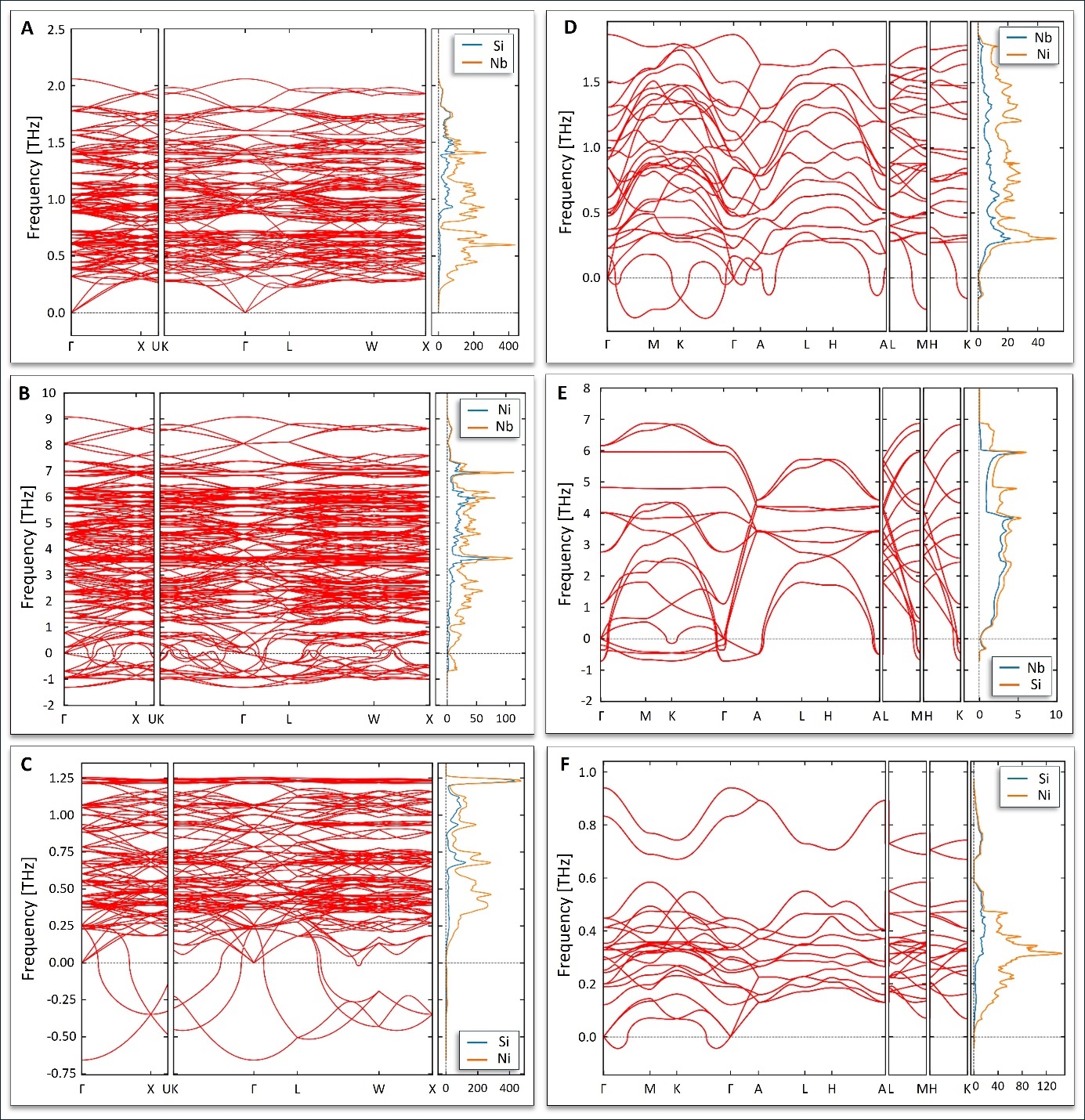


**Figure S11.** Predicted phonon dispersion and atom-projected PDOS derived at different temperatures for the derivative binary compounds Nb_3_Si at 300 K (A), Nb_3_Ni_2_ at 300 K (B), and SiNi_2_ at 300 K (C) of stoichiometric *fcc* Nb_3_SiNi_2_. Only the Nb_3_Si binary compound appears to be thermodynamically stable at RT. Predicted phonon dispersion and atom-projected PDOS derived at different temperatures for the derivative binary compounds Ni_3_Nb_2_ at 300 K (D), SiNb_2_ at 600 K (E), and Ni_3_Si at 300 K (F) of stoichiometric hexagonal Ni_3_SiNb_2_. None of the binary compounds of Ni_3_SiNb_2_ are thermodynamically stable under these conditions; moreover, binary compound SiNb_2_ does not appear to melt up to 600 K.

*S3.2. Bond Analysis*

The electronic density of states (eDOS) displays a metallic behavior according to calculations using both hybrid DFT HSE06 (Figure S12A) and DFT PBE (Figure S12B). The bond analysis has been performed on the crystal structure of the hexagonal Ni_3_SiNb_2_ ZIP phase variant and was minimized using hybrid DFT HSE06. Charge analysis is presented in Table S5, which reports the Bader charges for all atoms in the unit cell.

The 2D projection of the charge density of the plane [110] in hexagonal Ni_3_SiNb_2_ is plotted in Figure S12C as a visualization of the interactions between the constituent atoms. There is no charge density between atoms and, as such, the bonding network is ionic.


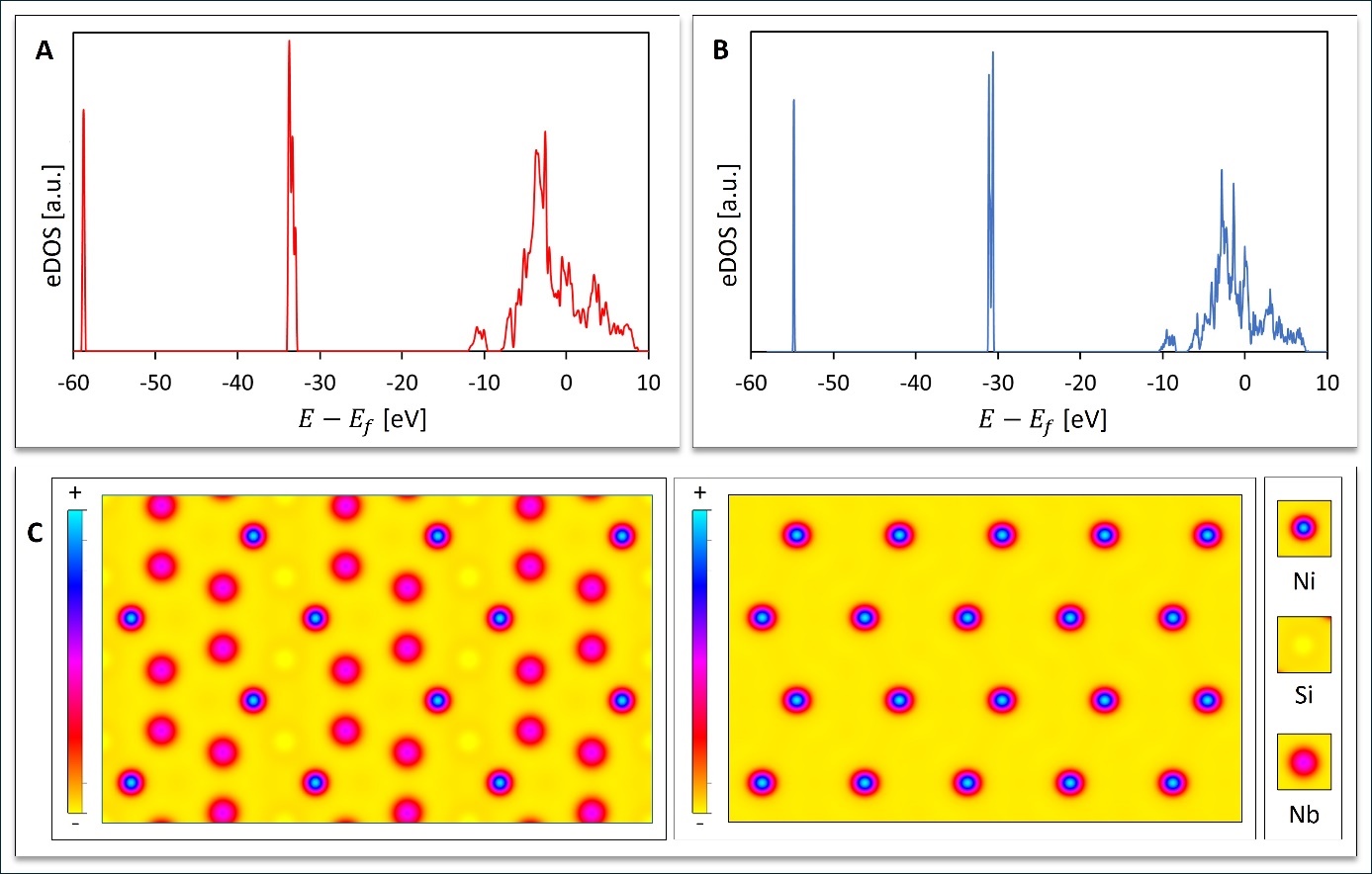


**Figure S12.** The electronic density of states (eDOS) of the hexagonal Ni_3_SiNb_2_ ZIP phase, using HSE06 (A) and PBE (B) functionals. The Fermi energy is at 0 eV. 2D data profiles of the charge density of plane [110] in hexagonal Ni_3_SiNb_2_, taken at two different heights.

Critical points (CP) of a scalar field are points where the gradient of the field vanishes, according to the quantum theory of atoms in molecules (QTAIM). Figure S13 presents the portion of the bond-critical points (BCPs) in the hexagonal Ni_3_SiNb_2_ ZIP phase variant. In this analysis, the BCPs are halfway along the bond lengths between different atoms, which is again indicative of the ionic nature of Ni_3_SiNb_2_.

The non-covalent interaction (NCI) simulations used to probe the low electron density regions do not display any van der Waals-like interactions in hexagonal Ni_3_SiNb_2_ (Figure 9F). Such analysis provides a qualitative visualization based on the electron density and its derivatives, enabling identification of non-covalent interactions based on the peaks that appear in the reduced density gradient at low densities. As shown in Figure 9F, there are no regions of strong interactions between the atoms, indicating ionic bonding.^[94,95]^

A more quantitative analysis of the electronic topology has been performed by analyzing the electron charge density, $\rho\left( \vec{r} \right),$ and its Laplacian, $\nabla^{2}\rho\left( \vec{r} \right),$ at BCPs, which are the points of minimum electron density between two atoms following the electron density gradient and can determine the bonding character (Table S6). As $\rho\left( \vec{r} \right)$ is an approximate measure of the amount of electron density in the bonding region, it can be interpreted as a characteristic of the bond. A hypothetical pure ionic bond would have a $\rho\left( \vec{r} \right)=0$, but predominantly ionic bonds have low values of $\rho\left( \vec{r} \right)$. This, in combination with a positive Laplacian, indicates ionic bonds between all species in hexagonal Ni_3_SiNb_2_.^[96]^

**Table S5.** The Bader charges of all atoms in the unit cell of hexagonal Ni_3_SiNb_2_.

| **Atom** | **X** | **Y** | **Z** | **Bader net atomic charges** |
| --- | --- | --- | --- | --- |
| Nb | 2.437581 | 1.407335 | 7.036751 | 0.94 |
| Nb | -2E-06 | 2.814675 | 0.494954 | 0.94 |
| Nb | -2E-06 | 2.814675 | 3.270899 | 0.99 |
| Nb | 2.437581 | 1.407335 | 4.260806 | 0.99 |
| Si | 0.000000 | 0.000000 | 0.000000 | -0.11 |
| Si | 0.000000 | 0.000000 | 3.765852 | -0.11 |
| Ni | 0.000000 | 1.423299 | 5.648779 | -0.60 |
| Ni | 2.437579 | 2.798711 | 1.882926 | -0.60 |
| Ni | 1.204966 | 3.510361 | 5.648779 | -0.61 |
| Ni | 1.232613 | 0.711649 | 1.882926 | -0.61 |
| Ni | -1.20497 | 3.510361 | 5.648779 | -0.61 |
| Ni | 3.642544 | 0.711649 | 1.882926 | -0.61 |

**Figure S13.** The positions of the BCPs in hexagonal Ni_3_SiNb_2_. The BCPs are indicated as pinkish dots along the bonds between atoms.

**Table S6.** Values of the electron charge density, $\rho\left( \vec{r} \right),$ and its Laplacian, $\nabla^{2}\rho\left( \vec{r} \right)$, for all BCPs in hexagonal Ni_3_SiNb_2_.

| **Bonds** | **Multiplicity** | $\boldsymbol{\rho}\left( \vec{\boldsymbol{r}} \right)$ **[a.u.]** | $\boldsymbol{\nabla}^{\boldsymbol{2}}\boldsymbol{\rho}\left( \vec{\boldsymbol{r}} \right)$ **[a.u.]** |
| --- | --- | --- | --- |
| Si-Ni | 12 | 3.74582133×10^-17^ | 1.72783362×10^-2^ |
| Ni-Ni | 6 | 3.23959580×10^-16^ | 7.34863881×10^-2^ |
| Ni-Ni | 6 | 6.87738426×10^-13^ | 8.57886084×10^-2^ |
| Nb-Nb | 2 | 7.42513401×10^-17^ | 3.20597636×10^-2^ |
| Nb-Ni | 12 | 1.95005025×10^-13^ | 5.12589906×10^-2^ |
| Nb-Ni | 24 | 5.45535306×10^-15^ | 4.66802833×10^-2^ |
| Nb-Nb | 6 | 2.87684444×10^-14^ | 2.69095712×10^-2^ |
| Nb-Si | 12 | 7.30834661×10^-18^ | 6.00538948×10^-2^ |

*S3.3. Surface Thermodynamic Stability – Potential Formation of 2D Derivatives*

The etching of stoichiometric ternary IMCs (ZIP phases) along specific planes is expected to form new surfaces, the stability of which is inherently associated with the stability of 2D derivatives of the ZIP phases, the experimental synthesis of which must still be demonstrated in the lab. As mentioned earlier, the 2D derivatives of the ZIP phases – especially the 2D derivatives of their hexagonal variants – are expected to be analogous (in terms of geometry, ability to be functionalized with various terminations, etc.) to the 2D derivatives of the MAX phases, broadly known as MXenes.

**Table S7.** Surface energies calculated for the Nb_3_SiNi_2_ and Ni_3_SiNb_2_ ZIP phase variants, using DFT without the inclusion of temperature effects.

| **ZIP Phase** | **Surface Miller index** | **Termination** | | **Thickness in number of surface repeat units** | **Surface energy [J/m^2^]** |
| --- | --- | --- | --- | --- | --- |
| **hexagonal Ni_3_SiNb_2_** | (001) | | Si | 2 | 2.49 |
|  |  |  |  | 4 | 2.53 |
|  |  |  |  | 6 | 2.53 |
| **hexagonal Ni_3_SiNb_2_** | (1$\bar{1}$0) | | NbNi | 1 | 2.33 |
|  |  |  |  | 2 | 2.37 |
|  |  |  |  | 3 | 2.38 |
| **hexagonal Ni_3_SiNb_2_** | (1$\bar{1}$0) | | Si | 1 | 2.31 |
|  |  |  |  | 2 | 2.31 |
|  |  |  |  | 3 | 2.31 |
| ***fcc* Nb_3_SiNi_2_** | (010) | | Nb | 1 | 2.49 |
|  |  |  |  | 2 | 2.65 |
|  |  |  |  | 3 | 2.65 |
| ***fcc* Nb_3_SiNi_2_** | (010) | | Si | 1 | 2.47 |
|  |  |  |  | 2 | 2.48 |
|  |  |  |  | 3 | 2.46 |
| ***fcc* Nb_3_SiNi_2_** | (101) | | Si | 1 | 2.54 |
|  |  |  |  | 2 | 2.62 |
|  |  |  |  | 3 | 2.69 |

In this work, surface models of the stoichiometric hexagonal Ni_3_SiNb_2_ and *fcc* Nb_3_SiNi_2_ ZIP phases have been selected. The scoping research done on this important topic is far from exhaustive, as proving the potential synthesis of 2D derivatives of the ZIP phases falls beyond the scope of this work, however, it permitted some interesting observations. All minimized surfaces resemble the initial models, with certain amounts of contraction of the surface atoms inwards and towards the material bulk (Figure S14, Figure S15). Although we have not included any kinetic effects, the surface energies have converged (see Table S7), suggesting that such nanolayers could, in principle, be generated experimentally without any energetic loss when compared to thicker layers. Although marginally, the Si-terminated surfaces were generally the most thermodynamically stable, suggesting that possibly these planes in the hexagonal Ni_3_SiNb_2_ ZIP phase might be the easiest to exfoliate.

**Figure S14.** The structures of the (A-C) (010), (D-F) (010), and (G-I) (101) surfaces in *fcc* Nb_3_SiNi_2_ before (left images in all three cases) and after (right images in all three cases) energy minimization. The sets of structures in (A-C) assume Si terminations, in (D-F) Nb terminations, and in (G-I) Si terminations. (A,D,G) consider only one surface repeat unit; (B,E,H) consider two surface repeat units; and (C,F,I) consider three surface repeat units.

**Figure S15.** The structures of the (A-C) (001), and (D-I) (0$\bar{1}$1) surfaces in hexagonal Ni_3_SiNb_2_ before (left images in all three cases) and after (right images in all three cases) energy minimization. The sets of structures in (A-F) assume Si terminations, and in (G-I) NbNi terminations. (A,D,G) consider only one surface repeat unit; (B,E,H) consider two surface repeat units; and (C,F,I) consider three surface repeat units.

As the bond analysis (see sections 2.2.5 & S3.2) has shown ionic behavior with low charge density around the silicon (Si) atoms, the stability of some Si-terminated surfaces has been calculated. These calculations have shown that the (1$\bar{1}$0) planes are more stable than the (001) planes (2.31 J/m^2^ vs. 2.53 J/m^2^), suggesting that the hexagonal Ni_3_SiNb_2_ ZIP phase might be easier to exfoliate along (1$\bar{1}$0) planes. These findings shed some first light on the fabrication of ZIP phase 2D derivatives, which are envisioned as similar to the MAX phase 2D derivatives (i.e., MXenes) when produced via the selective chemical etching of the hexagonal ZIP phase variants. Before closing this discussion, it is important to note that the assessment of the stability (or not) of binary compounds and/or ‘bare’ surfaces stemming from the exfoliation of the ZIP phases along specific planes is only indicative of the possible formation of their 2D derivatives. As in the case of MXenes, one is obliged to assess the stability of surfaces with various terminations (e.g., -O/-OH/-F) that react with the surface dangling bonds, rendering them stable. However, addressing the stability of surfaces with terminations falls completely outside the scope of this work.

**S4. Characterization of ZIP Phases in Other Ternary Systems**

This section presents the Rietveld refinement results of the XRD patterns of SPS/RHP intermetallic alloy samples produced in the Nb-Si-Co (Figure S16, Table S8), Ta-Si-Ni (Figure S17, Table S9), V-Si-Ni (Figure S18, Table S10), and Nb-Si-Fe (Figure S19, Table S11) ternary systems. The analyzed Nb-Si-Co, Ta-Si-Ni, V-Si-Ni, and Nb-Si-Fe intermetallic alloy samples were sintered at 1723 K, 1623 K, 1523 K, and 1723 K, respectively.

It is worthwhile noting that the previously reported structure of the Nb_4_Co_2_O phase^[97]^ is similar to a slightly expanded *fcc* Nb_3_SiCo_2_ ZIP phase structure, with the interstitial spaces being populated by O atoms, and partial Co/Nb solid solutioning allowed on the 32e (Co-site) and 16d (Si-site) Wyckoff sites. In this sense, the Nb_4_Co_2_O phase can be interpreted as the *fcc* Nb_3_SiCo_2_ ZIP phase with dissolved oxygen (O), which agrees with the higher observed O content of all *fcc* ZIP phase variants (see Figure 5A, Figure 10, Figure S7, and Figure S9) as compared to their hexagonal counterparts. Table S8 summarizes in tabular form the results of the Rietveld refinement of the XRD pattern (Figure S16) of the SPS Nb-Si-Co alloy intermetallic sample sintered at 1723 K in terms of phase assembly, lattice parameters of all constituent phases, and atomic site positions and occupancies in the *fcc* Nb_3_SiCo_2_ and hexagonal Co_3_SiNb_2_ ZIP phases.

Table S9 summarizes in tabular form the results of the Rietveld refinement of the XRD pattern (Figure S17) of the SPS Ta-Si-Ni alloy intermetallic sample sintered at 1623 K in terms of phase assembly, lattice parameters of all constituent phases, and atomic site positions and occupancies in the *fcc* Ta_3_SiNi_2_ and hexagonal Ni_3_SiTa_2_ ZIP phases.

Table S10 summarizes in tabular form the results of the Rietveld refinement of the XRD pattern (Figure S18) of the SPS V-Si-Ni alloy intermetallic sample sintered at 1523 K in terms of phase assembly, lattice parameters of all constituent phases, and atomic site positions and occupancies in the *fcc* V_3_SiNi_2_ ZIP phase.

Table S11 summarizes in tabular form the results of the Rietveld refinement of the XRD pattern (Figure S19) of the RHP Nb-Si-Fe alloy intermetallic sample sintered at 1723 K in terms of phase assembly, lattice parameters of all constituent phases, and atomic site positions and occupancies in the *fcc* Nb_3_SiFe_2_ and hexagonal Fe_3_SiNb_2_ ZIP phases.


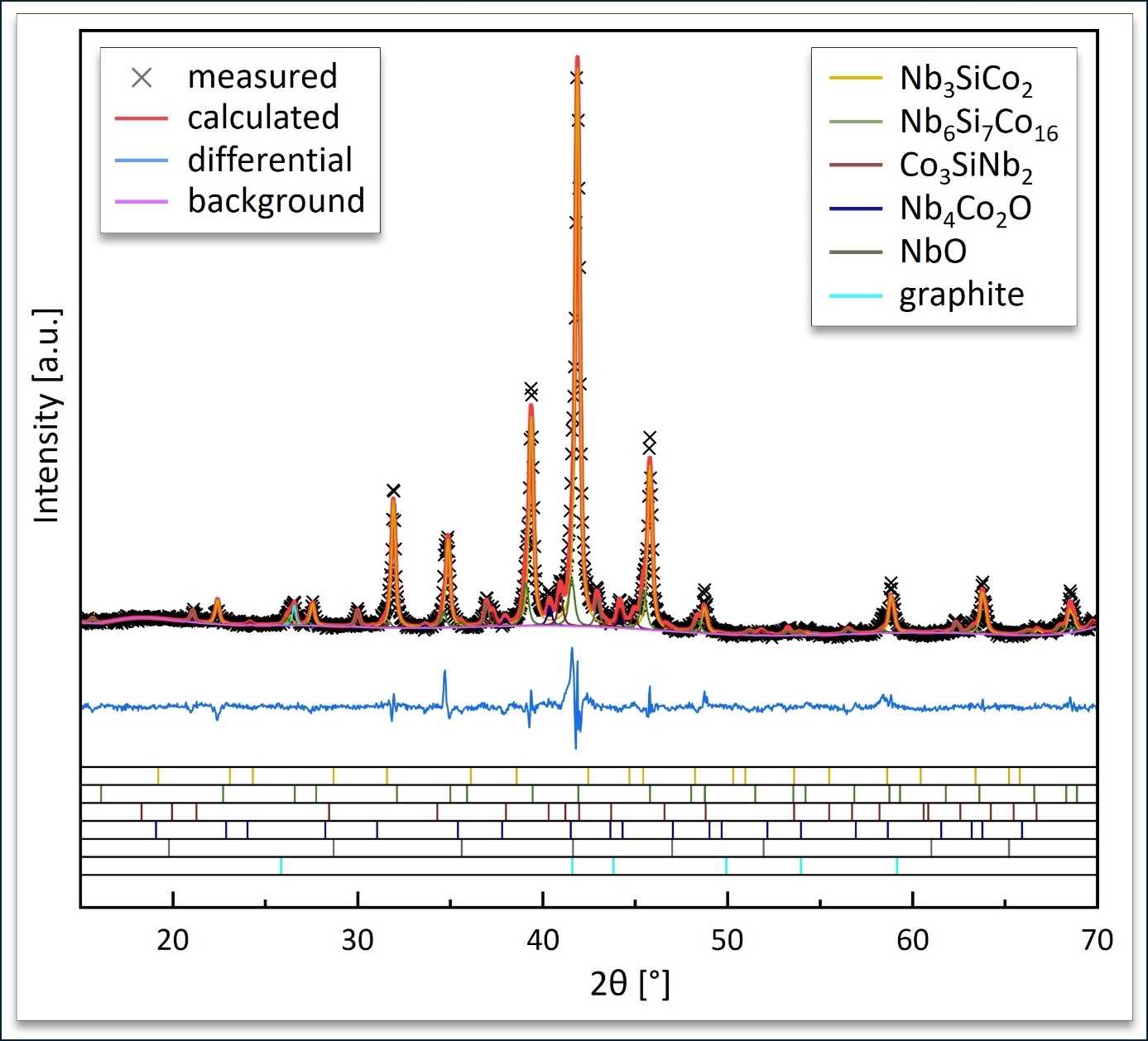


**Figure S16**. Rietveld-refined XRD pattern of the SPS Nb-Si-Co intermetallic alloy sample sintered at 1723 K. The main phases in this sample are the *fcc* Nb_3_SiCo_2_ ZIP phase, the hexagonal Co_3_SiNb_2_ ZIP phase, whilst some parasitic phases (i.e., Nb_6_Si_7_Co_16_, Nb_4_Co_2_O, NbO, and residual graphite) have also been detected.

**Table S8.** Results of Rietveld refinement of the XRD pattern of an SPS Nb-Si-Co sample sintered at 1723 K: lattice parameters of all phases; atomic site positions and occupancies in the *fcc* Nb_3_SiCo_2_ and hexagonal Co_3_SiNb_2_ ZIP phases.

| **Phase** | | **Nb_3_SiCo_2_** | **Nb_6_Si_7_Co_16_** | | **Co_3_SiNb_2_** | | **Graphite** | | **NbO** | | **Nb_4_Co_2_O** | |
| --- | --- | --- | --- | --- | --- | --- | --- | --- | --- | --- | --- | --- |
| PDF-code | | 01-070-2817 | 01-070-2845 | | 04-019-8391 | | 00-056-0159 | | 01-077-0015 | | 04-001-4308 | |
| Fraction [wt.%] | | 56.7 | 26.3 | | 5.9 | | 5.7 | | 3.3 | | 2.1 | |
| Space group | | $\text{Fd}\bar{\text{3}}\text{m}$ (227) | *Fm*$\bar{\text{3}}\text{m}$ (225) | | *P6_3_/mmc* (194) | | *P6_3_/mmc* (194) | | *Pm*$\bar{\text{3}}\text{m}$ (221) | | $\text{Fd}\bar{\text{3}}\text{m}$ (227)) | |
| *a* [Å] | | 11.201 | 11.282 | | 4.820 | | 2.461 | | 4.209 | | 11.601 | |
| *c* [Å] | |  |  | | 7.777 | | 6.703 | |  | |  | |
| *V* [Å^3^] | | 1405.191 | 1436.013 | | 156.472 | | 35.158 | | 74.584 | | 1561.502 | |
| Theoretical density [g/cm^3^] | | 8.029 | 7.489 | | 8.357 | | 2.260 | | 7.273 | | 8.595 | |
| Discrepancy indices | | R_wp_ = 3.16% | | | | | R_exp_ = 1.46% | | | | | |
|  |  | **Structural refinement of *fcc* Nb_3_SiCo_2_** | | | | | | | | | |  |
|  | Element | Wyckoff site | | *x* | | *y* | | *z* | | Occupancy | | |
| Nb | | 48f | | 0.1950 | | 0 | | 0 | | 1 | | |
| Si | | 16d | | 5/8 | | 5/8 | | 5/8 | | 1 | | |
| Co | | 32e | | 0.8350 | | 0.8350 | | 0.8350 | | 1 | | |
|  |  | **Structural refinement of hexagonal Co_3_SiNb_2_** | | | | | | | | | |  |
|  | Element | Wyckoff site | | *x* | | *y* | | *z* | | Occupancy | | |
| Nb | | 4f | | 1/3 | | 2/3 | | 0.5693 | | 1 | | |
| Si 1 | | 2a | | 0 | | 0 | | 0 | | 0.225 | | |
| Co 1 | | 2a | | 0 | | 0 | | 0 | | 0.775 | | |
| Si 2 | | 6h | | 0.1511 | | 0.3022 | | 1/4 | | 0.225 | | |
| Co 2 | | 6h | | 0.1511 | | 0.3022 | | 1/4 | | 0.775 | | |


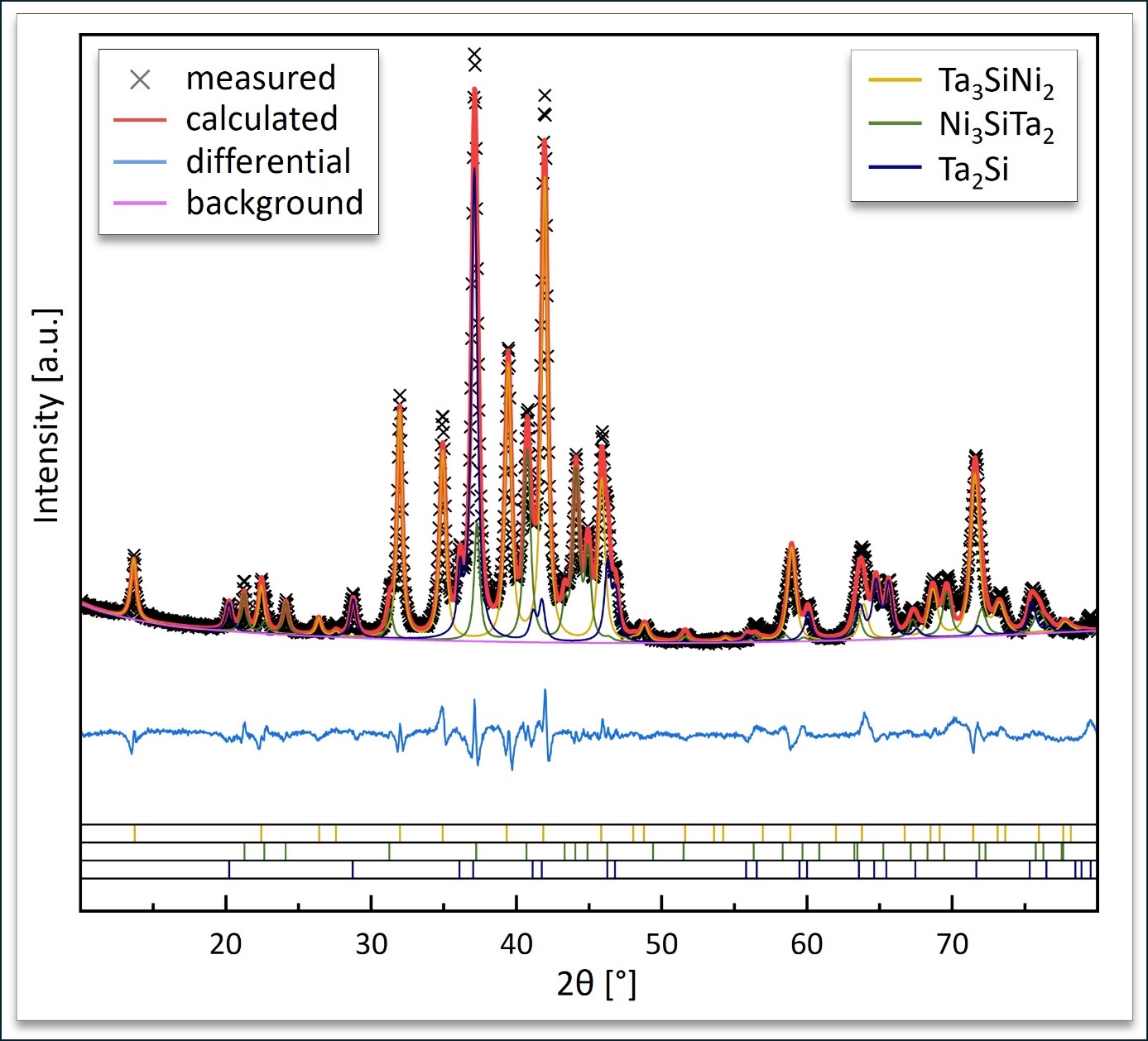


**Figure S17.** Rietveld-refined XRD pattern of the SPS Ta-Si-Ni intermetallic alloy sample sintered at 1623 K. The main phases in this sample are the *fcc* Ta_3_SiNi_2_ ZIP phase, the hexagonal Ni_3_SiTa_2_ ZIP phase, whilst a fraction of the Ta_2_Si parasitic phase has also been detected.

**Table S9.** Results of Rietveld refinement of the XRD pattern of an SPS Ta-Si-Ni sample sintered at 1623 K: lattice parameters of all phases; atomic site positions and occupancies in the *fcc* Ta_3_SiNi_2_ and hexagonal Ni_3_SiTa_2_ ZIP phases.

| **Phase** | | **Ta_3_SiNi_2_** | | **Ni_3_SiTa_2_** | | | | **Ta_2_Si** | |
| --- | --- | --- | --- | --- | --- | --- | --- | --- | --- |
| PDF-code | | 01-089-7168 | | 04-021-8936 | | | | 04-004-7298 | |
| Fraction [wt.%] | | 47.4 | | 25.9 | | | | 26.7 | |
| Space group | | $\text{Fd}\bar{\text{3}}\text{m}$ (227) | | *P6_3_/mmc* (194) | | | | *I4/mcm* (140) | |
| *a* [Å] | | 11.188 | | 4.820 | | | | 6.200 | |
| *c* [Å] | |  | | 7.830 | | | | 4.970 | |
| *V* [Å^3^] | | 1400.267 | | 157.533 | | | | 191.047 | |
| Theoretical density [g/cm^3^] | | 13.060 | | 11.934 | | | | 13.556 | |
| Discrepancy indices | | R_wp_ = 3.72% | | | | R_exp_ = 1.01% | | | |
|  |  | **Structural refinement of *fcc* Ta_3_SiNi_2_** | | | | | | | |
|  | Element | Wyckoff site | *x* | | *y* | | *z* | | Occupancy |
| Ta | | 48f | 0.4293 | | 1/8 | | 1/8 | | 1 |
| Si | | 16d | 0 | | 0 | | 0 | | 1 |
| Ni | | 32e | 0.2000 | | 0.2000 | | 0.2000 | | 1 |
|  |  | **Structural refinement of hexagonal Ni_3_SiTa_2_** | | | | | | | |
|  | Element | Wyckoff site | *x* | | *y* | | *z* | | Occupancy |
| Ta | | 4f | 1/3 | | 2/3 | | 0.5611 | | 1 |
| Si 1 | | 2a | 0 | | 0 | | 0 | | 0.25 |
| Ni 1 | | 2a | 0 | | 0 | | 0 | | 0.75 |
| Si 2 | | 6h | 0.1566 | | 0.3132 | | 1/4 | | 0.25 |
| Ni 2 | | 6h | 0.1566 | | 0.3132 | | 1/4 | | 0.75 |


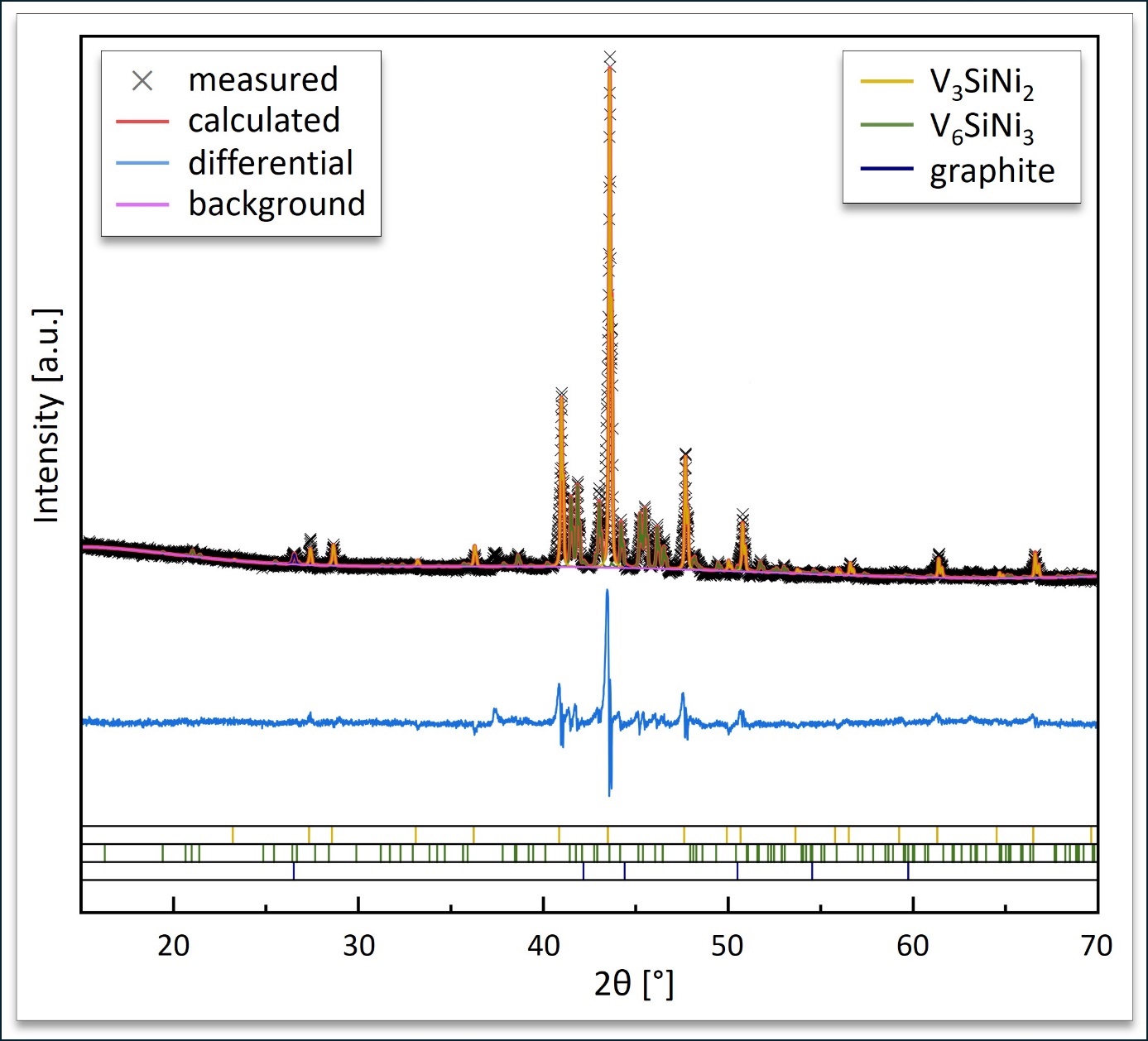


**Figure S18.** Rietveld-refined XRD pattern of the SPS V-Si-Ni intermetallic alloy sample sintered at 1523 K. The main phase in this sample is the *fcc* V_3_SiNi_2_ ZIP phase, with V_6_SiNi_3_ as a secondary phase.

**Table S10.** Results of Rietveld refinement of the XRD pattern of an SPS V-Si-Ni sample sintered at 1523 K: lattice parameters of all phases; atomic site positions and occupancies in the *fcc* V_3_SiNi_2_ ZIP phase.

| **Phase** | | **V_3_SiNi_2_** | | **V_6_SiNi_3_** | | | | **Graphite** | |
| --- | --- | --- | --- | --- | --- | --- | --- | --- | --- |
| PDF-code | | 04-008-0837 | | 04-022-9294 | | | | 00-056-0159 | |
| Fraction [wt.%] | | 58.3 | | 38.0 | | | | 3.7 | |
| Space group | | $\text{Fd}\bar{\text{3}}\text{m}$ (227) | | *R*$\bar{\text{3}}$ (148) | | | | *P6_3_/mmc* (194) | |
| *a* [Å] | | 10.773 | | 10.826 | | | | 2.465 | |
| *c* [Å] | |  | | 19.255 | | | | 6.706 | |
| *V* [Å^3^] | | 1250.288 | | 2556.730 | | | | 35.300 | |
| Theoretical density [g/cm^3^] | | 6.339 | | 6.888 | | | | 2.260 | |
| Discrepancy indices | | R_wp_ = 5.47% | | | | R_exp_ = 2.29% | | | |
|  |  | **Structural refinement of *fcc* V_3_SiNi_2_** | | | | | | | |
|  | Element | Wyckoff site | *x* | | *y* | | *z* | | Occupancy |
| V | | 48f | 0.1935 | | 0 | | 0 | | 1 |
| Si | | 16d | 5/8 | | 5/8 | | 5/8 | | 1 |
| Ni | | 32e | 0.8323 | | 0.8323 | | 0.8323 | | 1 |


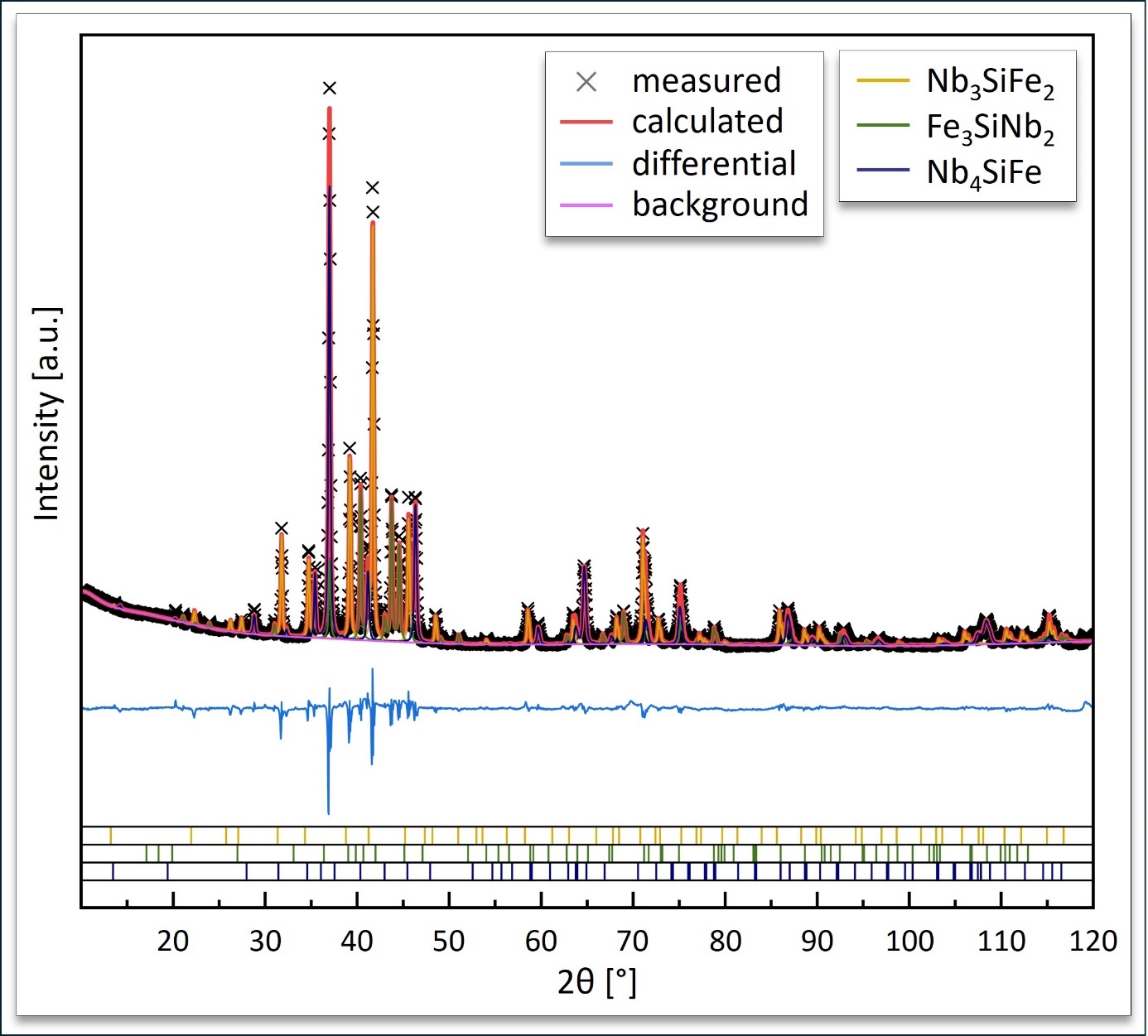


**Figure S19.** Rietveld-refined XRD pattern of the RHP Nb-Si-Fe intermetallic alloy sample sintered at 1723 K. The main phases in this sample are the *fcc* Nb_3_SiNb_2_ ZIP phase and the hexagonal Fe_3_SiNb_2_ ZIP phase, whilst a fraction of the Nb_4_SiFe parasitic phase has also been detected.

**Table S11.** Results of Rietveld refinement of the XRD pattern of an RHP Nb-Si-Fe sample sintered at 1723 K: lattice parameters of all phases; atomic site positions and occupancies in the *fcc* Nb_3_SiFe_2_ and hexagonal Fe_3_SiNb_2_ ZIP phases.

| **Phase** | | **Nb_3_SiFe_2_** | | **Fe_3_SiNb_2_** | | | | **Nb_4_SiFe** | |
| --- | --- | --- | --- | --- | --- | --- | --- | --- | --- |
| PDF-code | | / | | / | | | | 01-071-4455 | |
| Fraction [wt.%] | | 40.5 | | 27.4 | | | | 32.1 | |
| Space group | | $\text{Fd}\bar{\text{3}}\text{m}$ (227) | | *P6_3_/mmc* (194) | | | | *P4/mcc* (124) | |
| *a* [Å] | | 11.217 | | 4.841 | | | | 6.173 | |
| *c* [Å] | |  | | 7.881 | | | | 5.051 | |
| *V* [Å^3^] | | 1411.222 | | 159.925 | | | | 192.472 | |
| Theoretical density [g/cm^3^] | | 7.878 | | 7.978 | | | | 7.860 | |
| Discrepancy indices | | R_wp_ = 2.80% | | | | R_exp_ = 0.54% | | | |
|  |  | **Structural refinement of *fcc* Nb_3_SiFe_2_** | | | | | | | |
|  | Element | Wyckoff site | *x* | | *y* | | *z* | | Occupancy |
| Nb | | 48f | 0.1964 | | 0 | | 0 | | 1 |
| Si | | 16d | 5/8 | | 5/8 | | 5/8 | | 1 |
| Fe | | 32e | 0.8301 | | 0.8301 | | 0.8301 | | 1 |
|  |  | **Structural refinement of hexagonal Fe_3_SiNb_2_** | | | | | | | |
|  | Element | Wyckoff site | *x* | | *y* | | *Z* | | Occupancy |
| Nb | | 4f | 1/3 | | 2/3 | | 0.5643 | | 1 |
| Si 1 | | 2a | 0 | | 0 | | 0 | | 0.225 |
| Fe 1 | | 2a | 0 | | 0 | | 0 | | 0.775 |
| Si 2 | | 6h | 0.1700 | | 0.3400 | | 1/4 | | 0.225 |
| Fe 2 | | 6h | 0.1700 | | 0.3400 | | 1/4 | | 0.775 |

**S5. Electronegativity & Atomic Radius Values of ZIP Phase-Forming Elements**

This work discovered that ZIP phase formation appears to follow specific trends in the electronegativity, *χ*, and atomic radius values of the elements comprising the ZIP phase ternary IMCs. Table S12 gives electronegativity and atomic radius values for previously reported *fcc* ZIP phase variants (i.e., Nb_3_SiNi_2_, Nb_3_SiCo_2_, Ta_3_SiNi_2_, Mn_3_SiNi_2_, V_3_SiNi_2_, Cr_3_SiNi_2_, Na_3_AuIn_2_, Na_3_AgIn_2_, and Mg_3_GaNi_2_), as well as for the predicted and synthesized in this work *fcc* Nb_3_SiFe_2_ ZIP phase.

**Table S12.** Electronegativity, *χ*, and atomic radii values of Z/I/P-elements encountered in experimentally synthesized ternary IMCs, herein regarded as *fcc* ZIP phase variants.

| **ZIP phase** | **Z-element** | **I-element** | **P-element** |
| --- | --- | --- | --- |
| **Nb_3_SiNi_2_** | **Nb** | **Si** | **Ni** |
| Electronegativity | 1.60 | 1.90 | 1.91 |
| Atomic radius (pm) | 143 | 118 | 125 |
| **Nb_3_SiCo_2_** | **Nb** | **Si** | **Co** |
| Electronegativity | 1.60 | 1.90 | 1.88 |
| Atomic radius (pm) | 143 | 118 | 125 |
| **Ta_3_SiNi_2_** | **Ta** | **Si** | **Ni** |
| Electronegativity | 1.50 | 1.90 | 1.91 |
| Atomic radius (pm) | 143 | 118 | 125 |
| **Mn_3_SiNi_2_** | **Mn** | **Si** | **Ni** |
| Electronegativity | 1.55 | 1.90 | 1.91 |
| Atomic radius (pm) | 137 | 118 | 125 |
| **V_3_SiNi_2_** | **V** | **Si** | **Ni** |
| Electronegativity | 1.63 | 1.90 | 1.91 |
| Atomic radius (pm) | 131 | 118 | 125 |
| **Cr_3_SiNi_2_** | **Cr** | **Si** | **Ni** |
| Electronegativity | 1.66 | 1.90 | 1.91 |
| Atomic radius (pm) | 125 | 118 | 125 |
| **Na_3_AuIn_2_** | **Na** | **Au** | **In** |
| Electronegativity | 0.93 | 2.40 | 1.78 |
| Atomic radius (pm) | 186 | 144 | 163 |
| **Na_3_AgIn_2_** | **Na** | **Ag** | **In** |
| Electronegativity | 0.93 | 1.93 | 1.78 |
| Atomic radius (pm) | 186 | 145 | 163 |
| **Mg_3_GaNi_2_** | **Mg** | **Ga** | **Ni** |
| Electronegativity | 1.31 | 1.81 | 1.91 |
| Atomic radius (pm) | 160 | 122 | 125 |
| **Nb_3_SiFe_2_** | **Nb** | **Si** | **Fe** |
| Electronegativity | 1.60 | 1.90 | 1.83 |
| Atomic radius (pm) | 143 | 118 | 124 |

**References of Supporting Information:**

[94] A. Otero-de-la-Roza, E.R. Johnson, J. Contreras-García, Revealing non-covalent interactions in solids: NCI plots revised, *Physical Chemistry Chemical Physics* 35 (**2012**) 12165-12172.

[95] E.R. Johnson, S. Keinan, P. Mori-Sánchez, J. Contreras-García, A.J. Cohen, W. Yang, Revealing noncovalent interactions, *Journal of the American Chemical Society* 132 (**2010**) 6498-6506.

[96] S.J. Prathapa, J. Held, S. van Smaalen, Topological properties of chemical bonds from static and dynamic electron densities, *Journal of Inorganic and General Chemistry* 639 (**2013**) 2047-2056.

[97] H. Holleck, F. Thümmler, Ternäre Komplex-carbide, -nitride und-oxide mit teilweise aufgefüllter Ti_2_Ni-Struktur, *Monatshefte für Chemie* 98 **(1967)** 133–134.
